# *Salmonella enterica* induces biogeography-specific changes in the gut microbiome of pigs

**DOI:** 10.3389/fvets.2023.1186554

**Published:** 2023-09-14

**Authors:** Joao Carlos Gomes-Neto, Natasha Pavlovikj, Nate Korth, Samantha A. Naberhaus, Bailey Arruda, Andrew K. Benson, Amanda J. Kreuder

**Affiliations:** ^1^Department of Food Science and Technology, University of Nebraska-Lincoln, Lincoln, NE, United States; ^2^Nebraska Food for Health Center, University of Nebraska-Lincoln, Lincoln, NE, United States; ^3^Holland Computing Center, University of Nebraska-Lincoln, Lincoln, NE, United States; ^4^Department of Veterinary Microbiology and Preventive Medicine, Iowa State University, Ames, IA, United States; ^5^Department of Veterinary Diagnostic and Production Animal Medicine, College of Veterinary Medicine, Iowa State University, Ames, IA, United States

**Keywords:** *Salmonella* Typhimurium, *Salmonella* 4,[5],12:i:- (Monophasic), *Prevotella*, gut microbiome, swine

## Abstract

Swine are a major reservoir of an array of zoonotic *Salmonella enterica* subsp. *enterica* lineage I serovars including Derby, Typhimurium, and 4,[5],12:i:- (a.k.a. Monophasic Typhimurium). In this study, we assessed the gastrointestinal (GI) microbiome composition of pigs in different intestinal compartments and the feces following infection with specific zoonotic serovars of *S. enterica* (*S*. Derby, *S*. Monophasic, and *S.* Typhimurium). 16S rRNA based microbiome analysis was performed to assess for GI microbiome changes in terms of diversity (alpha and beta), community structure and volatility, and specific taxa alterations across GI biogeography (small and large intestine, feces) and days post-infection (DPI) 2, 4, and 28; these results were compared to disease phenotypes measured as histopathological changes. As previously reported, only *S*. Monophasic and *S.* Typhimurium induced morphological alterations that marked an inflammatory milieu restricted to the large intestine in this experimental model. *S.* Typhimurium alone induced significant changes at the alpha- (Simpson’s and Shannon’s indexes) and beta-diversity levels, specifically at the peak of inflammation in the large intestine and feces. Increased community dispersion and volatility in colonic apex and fecal microbiomes were also noted for *S.* Typhimurium. All three *Salmonella* serovars altered community structure as measured by co-occurrence networks; this was most prominent at DPI 2 and 4 in colonic apex samples. At the genus taxonomic level, a diverse array of putative short-chain fatty acid (SCFA) producing bacteria were altered and often decreased during the peak of inflammation at DPI 2 and 4 within colonic apex and fecal samples. Among all putative SCFA producing bacteria, *Prevotella* showed a broad pattern of negative correlation with disease scores at the peak of inflammation. In addition, *Prevotella 9* was found to be significantly reduced in all *Salmonella* infected groups compared to the control at DPI 4 in the colonic apex. In conclusion, this work further elucidates that distinct swine-related zoonotic serovars of *S. enterica* can induce both shared (high resilience) and unique (altered resistance) alterations in gut microbiome biogeography, which helps inform future investigations of dietary modifications aimed at increasing colonization resistance against *Salmonella* through GI microbiome alterations.

## 1. Introduction

According to the Centers for Disease Control and Prevention (CDC), approximately 50 million people acquire foodborne-associated illnesses in the United States annually (1). *Salmonella* is a global pathogen, with foodborne salmonellosis leading to over a million infections, thousands of hospitalizations, and hundreds of deaths yearly in the United States alone (2). Livestock-related foods and products are the predominant zoonotic source of human salmonellosis (3). *Salmonella* is primarily divided into two species, namely *S. enterica* and *S. bongori*, with the vast majority of zoonotic serovars and human clinical isolates belonging to *S. enterica* subsp. *enterica* lineage I (referred to as *S. enterica* lineage I) (4). Swine are a major reservoir of an array of zoonotic *S. enterica* lineage I serovars including Derby, 4,[5],12:i:- (a.k.a. Monophasic Typhimurium), and Typhimurium (3, 5–7). All three of those serovars are capable of residing in the gastrointestinal (GI) tract of swine and can spread across the food chain and cause human outbreaks (3, 5, 6, 8). *Salmonella* Derby was initially isolated from humans upon consumption of contaminated pork pies (9), and is now expected to be found in all major centers of pig production worldwide (5, 6); it has also recently been associated with infant outbreaks in China (10). On the other hand, the population of *S.* Typhimurium can be stratified into two divergent sub-lineages: Typhimurium and 4,[5],12:i:- (Monophasic) (11, 12). *Salmonella* Typhimurium isolates are typically known as host generalists and can reside in the GI tract of cattle, poultry, and swine, all of which can serve as a source of human outbreaks (3, 13), while *S*. Monophasic is an emergent zoonotic pathovar that can be isolated from cattle and poultry, but is most commonly found in the GI tract of swine (8, 13, 14). *S*. Monophasic derives from *S.* Typhimurium but uniquely contains an integrative and conjugative element (ICE) called *Salmonella* genomic island (SGI)-3/4 which phenotypically confers resistance to heavy-metals such as copper, arsenate, and silver and is inserted at a location which disrupts normal phase variation (11, 15–18). Copper resistance phenotypes of *S*. Monophasic under both aerobic and anaerobic conditions suggest SGI-3/4 may contribute to survival across natural environments (e.g., water reservoirs and food facilities) and may preferentially lead to a fitness advantage in swine potentially due to the utilization of dietary copper (11, 19). Additionally, there has been an increasing number of reports describing the global dissemination of multi-drug resistant (MDR) *S*. Monophasic isolates, posing further threat to public health (20–24).

Over time, many attempts have been made to mitigate *Salmonella* (largely focused in recent years on *S.* Typhimurium) in the swine industry by developing farm-based strategies to decrease prevalence (e.g., dietary changes and vaccination), improving diagnostics and surveillance, and increasing hygiene practices and regulations at food production facilities (3, 7, 25–30). Dietary modifications including the use of antimicrobials, probiotics, and prebiotics have been deployed to increase colonization resistance against *Salmonella*, partially through GI microbiome alterations (25, 31, 32). Additionally, it has been recently demonstrated that the GI microbiome composition is associated with variation in carriage of both *S*. Monophasic and *S.* Typhimurium (32–34), with *Prevotella* sp. being found to be negatively correlated with carriage and shedding (33–35). At the steady-state the swine GI tract is expected to have unique core microbiome composition at distinct anatomical sites (36), with *Prevotella* as a keystone taxon in weaned pigs until finishing stages (36, 37); this suggests microbiome-based signatures of colonization resistance to *Salmonella* are also likely to be distinct across different anatomical sites due to varying tissue tropism. Considering *S*. Monophasic and *S.* Typhimurium infection and inflammation can often be compartmentalized to specific segments of the large intestine (e.g., cecum and apex colonic tissues) (8), further studies are needed to assess the biogeography of the GI microbiome upon infection of pigs with distinct zoonotic *S. enterica* serovars to harness predictable colonization resistance signatures practically deployable to modern swine production systems. It is also important to note that *S.* Typhimurium, in general, can exploit the host inflammatory milieu to gain trans-intestinal epithelial access and persist in swine (32, 38). Colonization resistance to *Salmonella* is a complex trait that involves resistance (ability to resist a perturbation) and resilience (ability to recover from a perturbation) capacities of the GI microbiota (39).

Therefore, in this study we further explored a previously published experimental infection model in swine to assess how specific isolates of known zoonotic serovars of *S. enterica* (Derby, Monophasic, and Typhimurium) alter the GI microbiome biogeography overtime (day post-infection – DPI). Specifically, we performed 16S rRNA amplicon sequencing to map bacterial taxa that could be differentiated between infected and sham-inoculated groups across small and large intestinal samples as well as feces while measuring and analyzing: (1) alpha- and beta-diversity; (2) community structure and volatility; (3) relative abundance across taxa; and (4) correlation between specific taxa (relative abundance) and histopathology scores. Overall, our results showed (1) only *S*. Monophasic and Typhimurium could elicit overt transient inflammation (DPI 2 and 4) at the large intestine coupled with GI microbiome perturbations; (2) alpha- and beta-diversity differences were most distinct for the Monophasic and Typhimurium groups (unique signature) at DPI 2 and 4 in apex of the spiral colon and fecal contents; and (3) at the taxonomic level serovar unique and shared changes were induced demonstrating on average a transient inflammatory bottleneck (low resistance) that directly impacted the proportion and phenotypic correlations with putative short-chain fatty acid (SCFA) producing bacteria.

## 2. Materials and methods

### 2.1. Animal studies

A complete description of the animal studies from which the samples used in this study were taken is provided in Naberhaus et al. (8). While metagenomic samples were collected and sequencing data generated for all three of the previously described experimental studies, only data from trial 2 was utilized for this analysis as it provided a longitudinal and biogeographical analysis of multiple *Salmonella* serovars across multiple animals. In this study, all pigs (mixed sex) were 5 weeks of age at the beginning of the trial; this age was chosen based on retrospective epidemiological data accumulated in the past decade by the Iowa State University (ISU) Veterinary Diagnostic Laboratory (VDL). By pooled and individual PCR testing on fecal samples, all pigs tested negative for *Salmonella* prior to inoculation; all pigs also tested negative for porcine reproductive and respiratory syndrome virus (PRRSV) and porcine epidemic diarrhea virus (PEDV) prior to the initiation of the study. Pigs were individually identified and allocated to the following treatments: (1) 12 pigs sham-inoculated with sterile Mueller Hinton (MH) broth served as negative control; (2) 20 pigs received oral inoculation with *S*. Derby; (3) 20 pigs received oral inoculation with *S*. 4,[5],12:i:- (*S*. Monophasic); and (4) 20 pigs received oral inoculation with *S.* Typhimurium. To avoid cross-contamination, upon treatment allocation, each group was housed separately (four pigs per pen across all treatments) for the duration of the study. Inoculations were done utilizing a combination of 8 mL oral gavage and 2 mL swabbed directly in the back of the mouth ensuring tonsil exposure for a total of 10 mL of 1 × 10^8^ CFU/mL *Salmonella*. Pigs were fed *ad libitum* except for 12 h prior to inoculation and were euthanized using barbiturate overdose. All studies involving animals were approved by the Iowa State University Institutional Animal Care and Use Committee (IACUC) prior to initiation (11-16-8,391-S).

### 2.2. Sample collection

Prior to any other sample collection (i.e., – temperature, fecal sample for culture), fecal swabs for 16S rRNA based microbiome analysis were collected from the rectum of all pigs at DPI 0 and 2, and all pigs alive at DPI 4, 7, 14, 21 and 28. These samples were swirled in sterile Dubelco’s phosphate buffered saline (1X DPBS, Corning, Ref 21-030-CM) and immediately frozen in liquid nitrogen after collection and maintained at −80°C until sample processing. Gastrointestinal tract contents were collected from the small intestine (ileum) and large intestine (apex of the spiral colon) at the time of necropsy (DPI 2, 4 or 28). On DPI 2 and 4, five pigs per *Salmonella*-infected group and three control pigs were selected for euthanasia and necropsy based on the severity of clinical signs (i.e., *Salmonella*-infected pigs demonstrating the most severe clinical signs based on a combination of rectal temperature and fecal score). The remaining pigs after DPI 4 (10 per experimental group; 6 in control group) were allowed to complete the study and were euthanized for sample collection at DPI 28. At the time of euthanasia, a necropsy was performed to evaluate gross lesions. Tissue samples were collected and placed in 10% formalin for blinded histopathologic evaluation of the jejunum, ileum, cecum, mid-spiral colon, apex of the spiral colon (colonic apex), and rectum. Ileal and colonic apex contents collected at the time of necropsy were flash frozen in liquid nitrogen and maintained at −80°C until sample processing. Scrapings of the ileal mucosa and apex of the spiral colon mucosa were also taken, flash frozen in liquid nitrogen, and stored at −80°C.

### 2.3. Histopathology scoring system

A detailed description of the histopathology scoring system for each site examined can be found in Naberhaus et al. (8). In brief, histologic scores were assigned based on the presence of neutrophils, ulceration, and crypt depth (large intestine only); additional points were assigned if submucosal inflammation or crypt abscesses were present. For the purposes of correlating disease (i.e.- histologic score) with the metagenomics data, a cumulative histopathology score combining data recorded for all four of the large intestinal sites (cecum, mid-spiral colon, apex of the spiral colon, and the rectum) and small intestinal sites (proximal, mid, and distal jejunum; ileum) was developed. Of note, the colonic apex histopathological score was also used to check for associations with taxa of interest enriched at the apex contents.

### 2.4. DNA preparation and sequencing

DNA extraction was performed using the MagMAX™ Pathogen RNA/DNA kit (Thermo Fisher Scientific, Waltham, MA, United States) and a Kingfisher Flex instrument (Thermo Fisher Scientific) following the instructions of the manufacturer. Samples were then submitted to the Iowa State University DNA Facility for library preparation and 16S metagenomics sequencing. Extracted total DNA was amplified using primers (515F and 806R) specific to the V4 regions of the bacterial 16S rRNA gene and library preparation was performed as previously described (40–43). Following library preparation samples were run on 150 paired-end cycles on an Illumina MiSeq instrument. A total of 721 samples were submitted and sequenced on three lanes with a maximum number of samples per lane of 240. Negative controls consisting of sterile swabs in DPBS as well as a negative extraction control were included; positive controls consisting of Zymobiomics Microbial Community standards (catalog #D6305 and D6300; Zymo Research Corporation, Irvine, CA) were also included on all plates to ensure consistency across plates in sequencing.

### 2.5. 16S rRNA data analysis

The multiplexed data generated from the three MiSeq lanes was analyzed using QIIME2 (44), version 2021.11. The demultiplexed paired-end reads from each lane were denoised and reads with a quality score below the default Q20 value were trimmed and filtered out with DADA2 (45). To speed-up the computational analyses, this step was performed separately on each run of the three lanes. Then, the table and sequences from the separate DADA2 runs were merged with *qiime feature-table merge* and *qiime feature-table merge-seqs*. The data was then filtered to only include samples collected from trial 2. The updated feature table was later used to pick a sampling depth for the diversity analyses. Taxonomic analyses and assigning taxonomic units to each of the representative sequences were performed with *qiime feature-classifier classify-sklearn* and the SILVA reference database (*silva-138.1-ssu-nr99*). Mitochondria and chloroplast sequence were further removed using *qiime taxa filter-table*. Finally, a phylogenetic tree, as well as various diversity metrics (alpha and beta) and similarity analyses were performed using QIIME2.

### 2.6. Statistical analysis

Taxonomic outputs from QIIME2 were processed for quality control all the way to statistical modeling using R version 4.0.5. The tidyverse library (version 1.3.1) was used for data exploration, analysis, and plotting. All samples kept in the analysis passed the following combined criteria: “percentage of input passed filter” > 75 & “percentage of input merged” > 60 & “percentage of input non-chimeric” > 60. Thereafter, only taxon classified as “Bacteria” and that had genus level information were included in the analysis. Neither taxon classified as “Archaea” nor ones classified as uncultured at the genus level were included in the statistical analysis of microbiota composition. Individual taxon-based proportions were calculated per animal while accounting for DPI, treatment, and sample type. A one-way analysis of variance (ANOVA) was used to assess the effect of treatment on histopathology scores, alpha-diversity metrics (Shannon and Simpson’s D), beta-diversity decomposition and volatility analysis (PC1 or PC2), and individual taxon-based relative abundance (proportion). All ANOVA models were done using the aov() function in R. If the treatment effect was significant (*p* < 0.05) in the ANOVA model, then a pairwise *T*-test was used to assess differencse between groups (*p* < 0.05), unless stated otherwise. All pairwise *T*-tests were done using the pairwise.t.test() function in R without a *p*-value adjustment and by using a pooled standard deviation as default. Both Shannon and Simpson’s D indexes of alpha-diversity were calculated with the diversity() function in R from the vegan library (version 2.6.2). Beta-diversity was calculated using the vegdist function (method = “bray,” binary = “FALSE,” na.rm. = “TRUE”) function from the vegan library (version 2.6.2) while using a Bray-Curtis’s distance matrix of non-binary data and by removing all missing values of the analysis. For the principal coordinate analysis (PCoA) a classical multidimensional scaling model was used to reduce the data (Bray-Curtis’s distance matrix) to two dimensions (2 principal coordinates – PCs), using the cmdscale function (*k* = 2) in R from the stats library (version 4.0.5). A PERMANOVA and analysis of similarity (ANOSIM) were used to calculate the effect of treatment of beta-diversity (Bray-Curtis distances), using the adonis2(permutations = 999, method = “bray”) and anosim(method = “bray”) functions in R, respectively, from the vegan library (version 2.6.2). Pearson correlations between log_2_ transformed taxa proportion (both from colonic apex and feces) and large intestinal/colonic apex histopathology scores were calculated using the cor_test() function in R using default parameters, as part of the rstatix library (version 0.7.2). LEfSe analysis for identification of differentiating taxa was done with the Galaxy platform with a Wilcoxon *p*-value adjustment to 0.1, due to the small sample sizes across treatments, and upon removal of taxon or features with zero counts across all samples (46, 47). Ultimately, the most differentiating taxa were selected based on (1) overall relative proportion above 2%; (2) LEfSe results; and (3) central taxa on co-occurrence network analysis. Also, individualized, or average co-occurrence patterns (community composition) of microbiome changes were created using either taxon at different levels of resolution (e.g., family, genus), or the most differentiating taxa, respectively. Average based patterns were created using a log_2_ transformation of the mean proportion for each taxon across treatments and DPI. Across all major taxa identified, proportions were calculated by individual animal/sample, but for each animal/sample, proportions were summed up to eliminate taxonomic redundancy and inaccuracy when doing the final calculations (e.g., all taxon containing a “g__Prevotella” in the name were combined to *Prevotella* for genus level calculations). For all R functions used, if not stated, the default parameters were used for calculations.

### 2.7. Co-occurrence network analysis

Network analysis and data visualization were performed using the NetCoMi package within the R (v4.2.2) statistical framework (48). Each network was constructed for each treatment, by employing a centered log-ratio transformation and Spearman-based correlations between the 50 most abundant microbial taxa. Data interpretation was summarized based on each node representing a bacterial taxon, and the size of each node is scaled according to its centrality. Edges represented associations between taxa, with positive associations colored in green, and negative in red; the thickness of each edge corresponds to the strength of the association. Edges representing a value less than 0.5 were not shown. Taxa were colored based on clusters calculated in network construction.

### 2.8. Computational platforms

All 16S rRNA microbiome bioinformatic analyses were performed on Crane, one of the Linux high-performance computing clusters at the University of Nebraska Holland Computing Center (HCC).^1^

## 3. Results

### 3.1. Overview of experimental approach and disease phenotype across serovars

**Figure 1:**
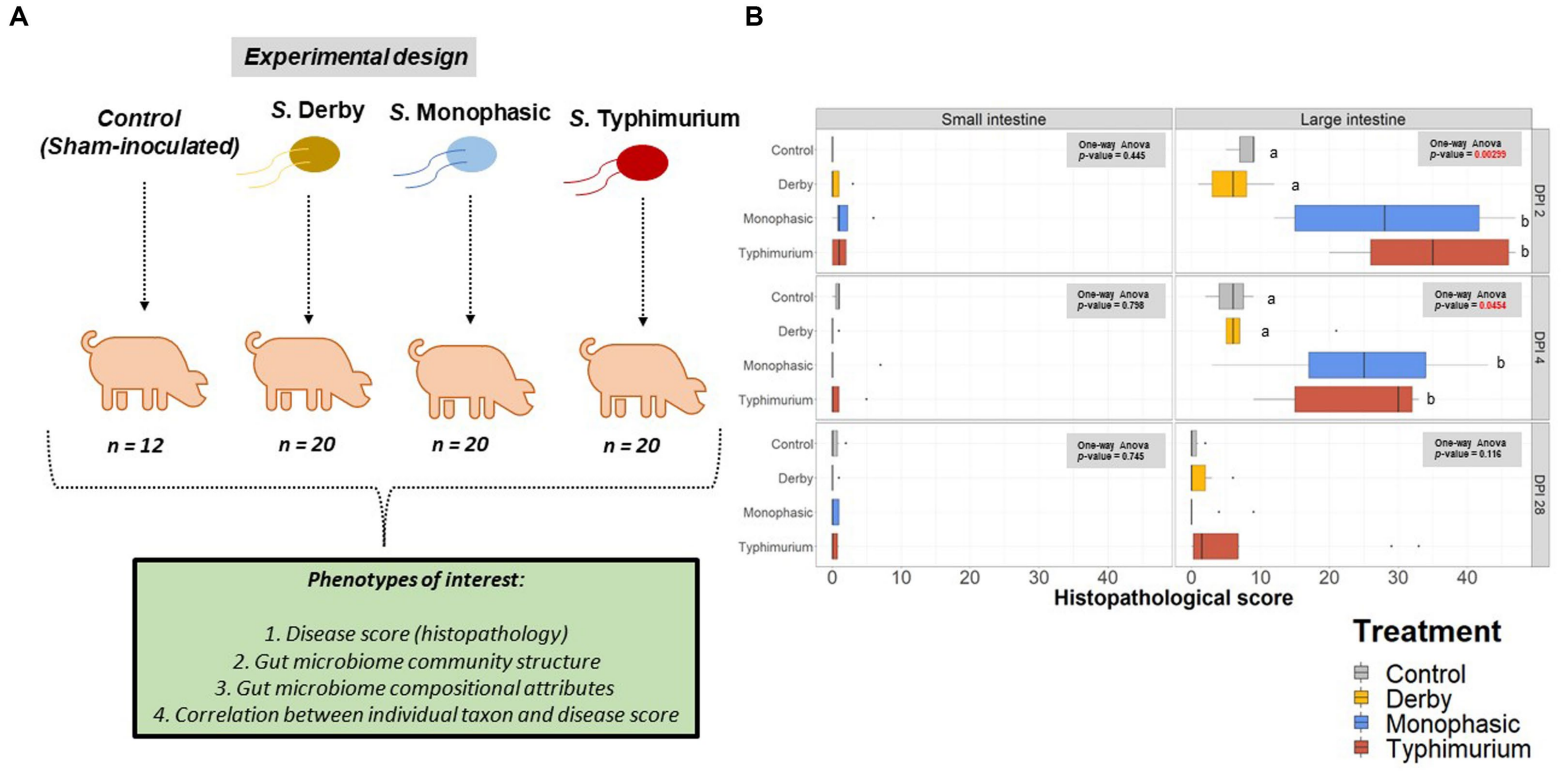
Experimental design and histopathology scores. **(A)** Experimental design workflow including the number of animals per treatment and phenotypes of interest, including histopathology (disease score), GI microbiome composition, taxon relative abundances, and correlation between individual taxon and histopathology scores. **(B)** Histopathology scores across the small intestine (ileum) and the large intestine at DPI 2, 4, and 28. A one-way ANOVA was used to measure the effect of treatment (control vs. *Salmonella* infected groups) (*p* < 0.05). When the treatment effect was significant based on the one-way ANOVA analysis (*p*-values marked in red), then pairwise comparisons were done using a two-sided *T*-test (*p* < 0.05). Different superscript letters indicate significant differences between treatments. The number of animals used per treatment can be found in the Supplementary Figure S1.

Using samples collected from our previous work assessing the pathogenicity of *S*. Derby, *S*. Monophasic, and *S.* Typhimurium (8) in swine, 16S rRNA based microbiome analysis was performed to evaluate the impact of different swine-associated zoonotic serovars of *S. enterica* lineage I on the GI microbiome biogeography. Figure 1A illustrates the experimental design and objectives of the study. Supplementary Figures S1A–C depicts the number of animals analyzed per treatment and DPI after microbiome data curation. As demonstrated in Figure 1B, only *S*. Monophasic and *S.* Typhimurium induced significant overt inflammation, restricted to the large intestine, during DPI 2 and 4 post-infection (*p* < 0.05); no inflammatory lesions were seen with *S.* Derby infection or in the small intestines for *S*. Monophasic and *S.* Typhimurium, as previously established (8). No significant differences in histopathology scores were found between *S*. Monophasic and *S.* Typhimurium in the large intestine (*p* ≥ 0.05). When the colonic apex histopathology was examined separately, no significant differences were found due to small sample size and variability, although on average *S*. Monophasic and *S.* Typhimurium infected animals presented higher scores (Supplementary Figure S2). Based on our previous report (8), shedding of *Salmonella* in this study peaked by DPI 2–4 simultaneous with peak inflammation and typically subsided 7–10 DPI. Overall, both *S*. Monophasic and *S.* Typhimurium had comparable disease kinetics restricted to the large intestine.

### 3.2. Overview of 16S rRNA amplicon sequencing results

The first MiSeq lane produced 15,135,350 paired-end reads, the second 15,825,865 paired-end reads, and the third 11,631,035 paired-end reads, where the forward and reverse reads and the respective barcodes were stored in separate files. After filtering, denoising and merging, the number of resulting reads was 7,983,113, 8,284,340, and 5,884,840 for Lanes 1, 2, and 3, respectively. After merging the individual runs and filtering the data to only include samples from trial 2, the total number of samples was 629 and the total number of representative sequences (features or ASVs) was 5,774, with a total frequency of 18,738,198. The minimum length of the features (sequences) was 152 bp, and the maximum length was 278 bp with 252.32 being the mean length. After removing mitochondria and chloroplast sequences, the total number of final samples used in our analyses was 626 and the total number of representative sequences (features or ASVs) was 5,733, with a total frequency of 18,729,244. The minimum length of the features (sequences) was 152 bp, and the maximum length was 255 bp with 252.38 being the mean length. Due to poor sequence quality (see section 2.6), analysis of ileal and colonic apex scrapings was not able to be performed. To compare diversity across the ileal, colonic apex, and fecal contents at the same timepoint for each animal, analysis was primarily focused on samples collected on DPI 2, 4, and 28 except a cross-sectional temporal analysis for fecal samples on DPI 0, 2, 4, 7, 14, 21, and 28.

### 3.3. Alpha- and beta-diversity changes are acutely captured at the site of inflammation and feces

**Figure 2:**
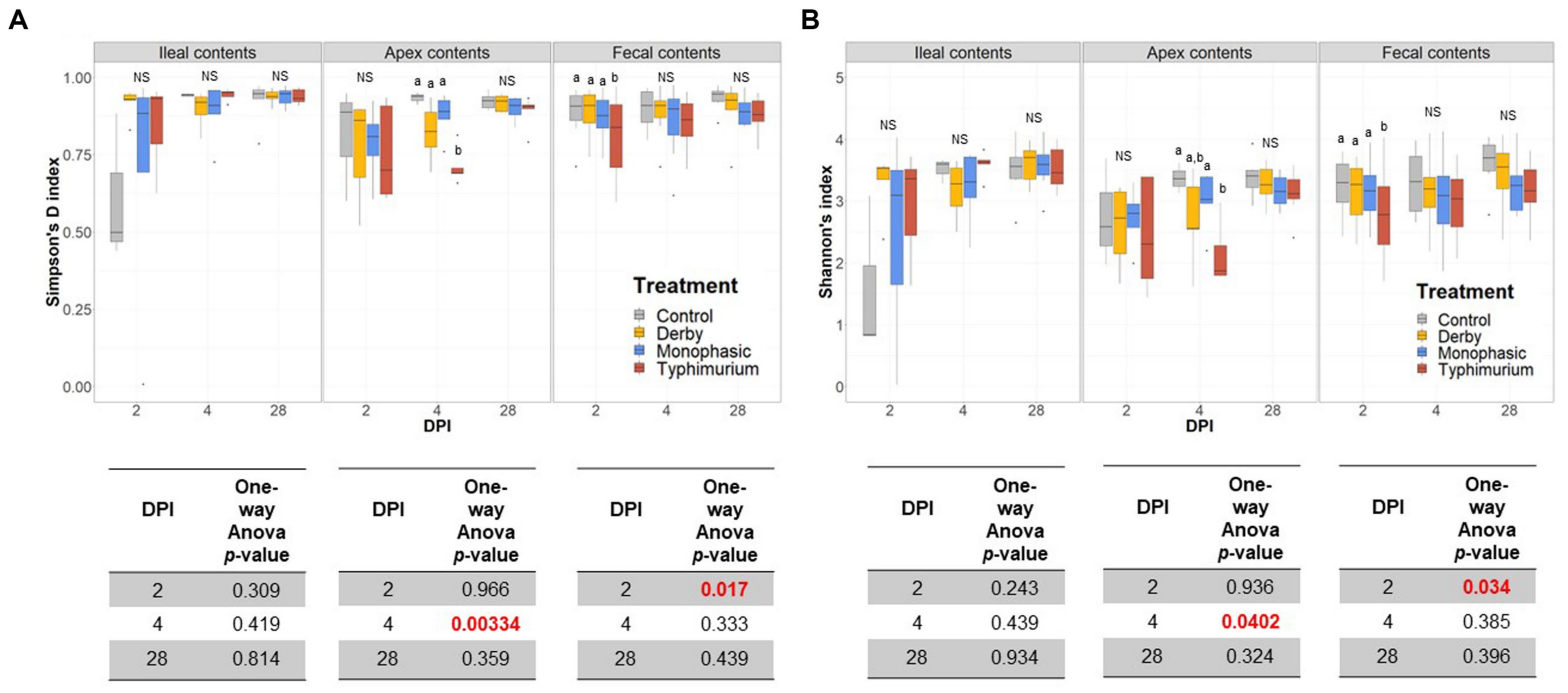
Alpha-diversity analysis of the GI microbiome composition across control vs. *Salmonella* infected groups. **(A,B)** Simpson’s D and Shannon indexes of alpha-diversity across treatments and sample types including ileal, colonic apex, and fecal contents of pigs at DPI 2, 4, and 28, respectively. For both metrics of alpha-diversity, a one-way ANOVA analysis was used to measure the effect of treatment (*p* < 0.05). When the treatment effect was significant based on the one-way ANOVA analysis (*p*-values marked in red), then pairwise comparisons were done using a two-sided *T*-test (*p* < 0.05). Different superscript letters indicate significant differences between treatments. The number of animals used per treatment can be found in the Supplementary Figure S1.

**Figure 3:**
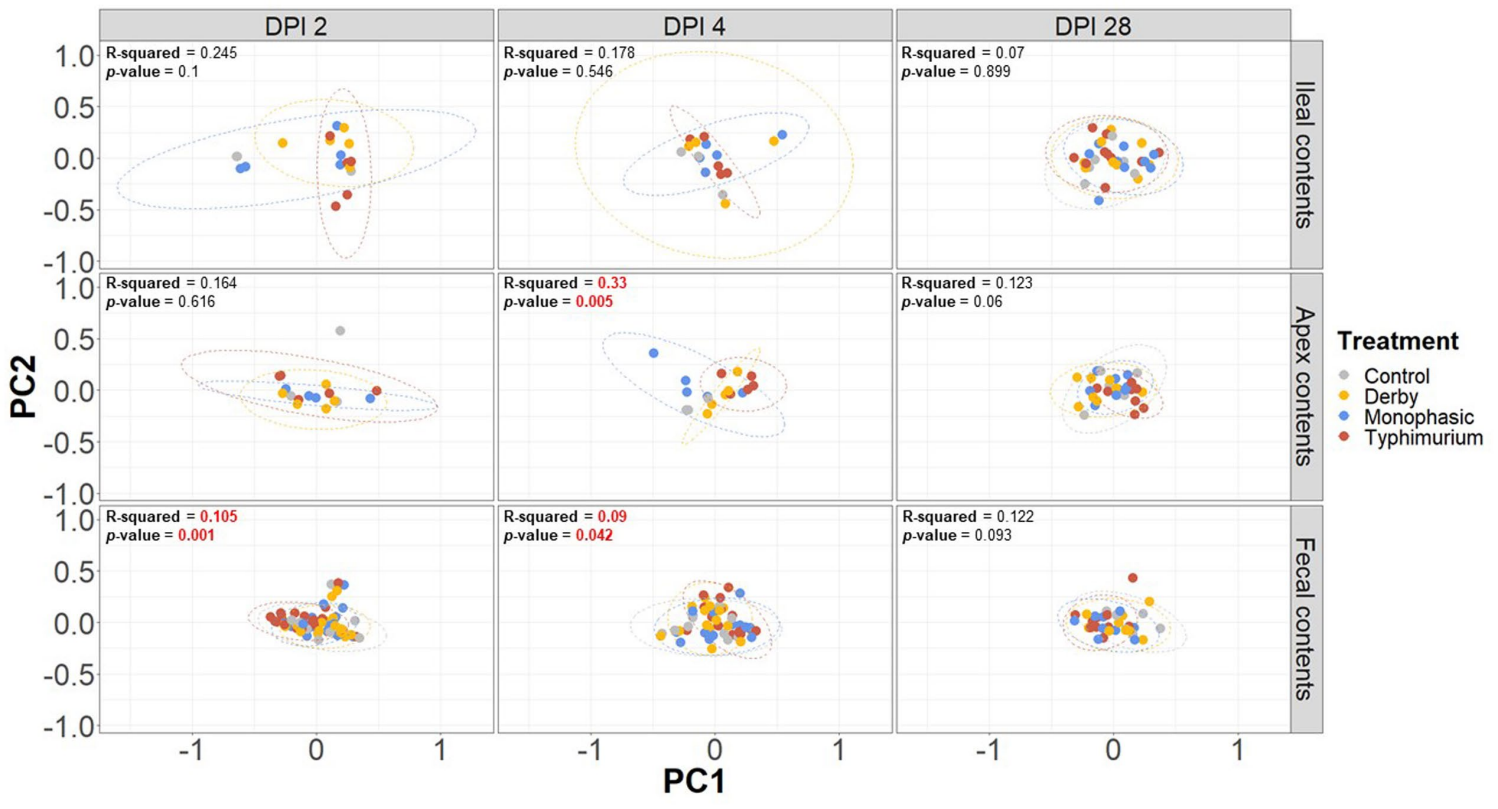
Beta-diversity analysis of the GI microbiome composition across control vs. *Salmonella* infected groups. The Bray-Curtis distance matrix was used to calculate the beta-diversity between treatments. Two principal coordinates are shown across DPI 2, 4, and 28, and all sample types (ileal, colonic apex, and fecal contents). A PERMANOVA model was used to assess the treatment effect on beta-diversity. *p*-values and R-squared statistics are shown in each plot. Significant differences were considered based *p* < 0.05 (results marked in red). The number of animals used per treatment can be found in the Supplementary Figure S1.

Biogeographic differences in alpha-diversity were analyzed by utilizing the Simpson’s D and Shannon’s indexes across the ileal, colonic apex, and fecal contents for DPI 2, 4, and 28. Both Simpson’s and Shannon’s indexes highlighted that on average, only *S.* Typhimurium infection significantly lowered alpha-diversity in fecal samples at DPI 2 and in the large intestine (colonic apex) at DPI 4 (peak of inflammation) (Figures 2A,B) (*p* < 0.05). Nonetheless, changes in alpha-diversity were transient since there was no effect of treatment at DPI 28 for both indexes (*p* ≥ 0.05). Of note, it appears that on average, the *S.* Typhimurium infected group alpha-diversity is reestablished in colonic apex samples by DPI 28 (Figures 2A,B). A more sequential cross-sectional analysis of alpha-diversity in fecal contents also revealed a transient decrease in the *S.* Typhimurium infected group by DPI 21 (*p* < 0.05) (Supplementary Figures S3, S4). Interestingly, both Shannon’s and Simpson’s D indexes suggest that after the peak of inflammation (DPI 4), both *S*. Monophasic and *S.* Typhimurium groups tended to remain on average lower in diversity compared to DPI 0 in fecal samples (Supplementary Figures S3, S4). At the beta-diversity level (Bray-Curtis’s distance), community dispersion mirrored the alpha-diversity results with significant changes captured at DPI 4 for colonic apex and fecal contents (Figure 3) (*p* < 0.05). Of note, significant changes in beta-diversity at DPI 2 were only found for fecal contents (*p* < 0.05), while no significant changes were found in ileal contents. However, it is important to highlight that community dispersion was more clearly observed between treatments at the peak of inflammation in the colonic apex (*p*-value = 0.007 and R-squared = 0.33), based on PERMANOVA results (Figure 3 – DPI 4 for colonic apex samples). A higher R-squared value reflects a stronger biological effect of treatment since it explains more of the total variability in the data.

In addition to PERMANOVA-based modeling, an ANOSIM was used to assess community structure similarities, and *S.* Typhimurium-induced changes were significantly different from other treatments both at DPI 4 for colonic apex (R = 0.29, *p* = 0.004) and DPI 2 for fecal (R = 0.119, *p* = 0.001) samples, mirroring the PERMANOVA results (*p* < 0.05) (Supplementary Figures S5–S13). In accordance with the PERMANOVA results, the ANOSIM modeling strategy confirmed the absence of significant changes in community structure between treatments for ileal contents (*p* ≥ 0.05). Further decomposition of beta-diversity, as analyzed by PC1 or PC2 separately, also showed no significant changes in community dispersal for ileal samples (Supplementary Figures S14A–F) and confirmed the presence of significant alterations in the microbial community structure of *S.* Typhimurium infected animals at DPI 4 for colonic apex and DPI 2 for feces (*p* < 0.05) (Supplementary Figures S15, S16A–F). Last, a cross-sectional analysis of beta-diversity across fecal samples demonstrated significant differences in community structure for *S.* Typhimurium groups at DPI 7 and 21 (*p* < 0.05) (Supplementary Figure S17) in addition to DPI 2 and 4 as previously shown. In accordance, ANOSIM confirmed the significant changes identified in community structure at DPI 7 and 21 for fecal samples in the *S.* Typhimurium infected group (*p* < 0.05) (Supplementary Figures S18–S21). Therefore, the changes in alpha- and beta-diversity analyses illustrate a significant effect in community structure can occur during the inflammatory bottleneck in the GI microbiome suggesting a low resistance capacity, which uniquely distinguished the *S.* Typhimurium infected group.

### 3.4. Co-occurrence network and volatility analysis revealed serovar-specific alterations in community structure

**Figure 4:**
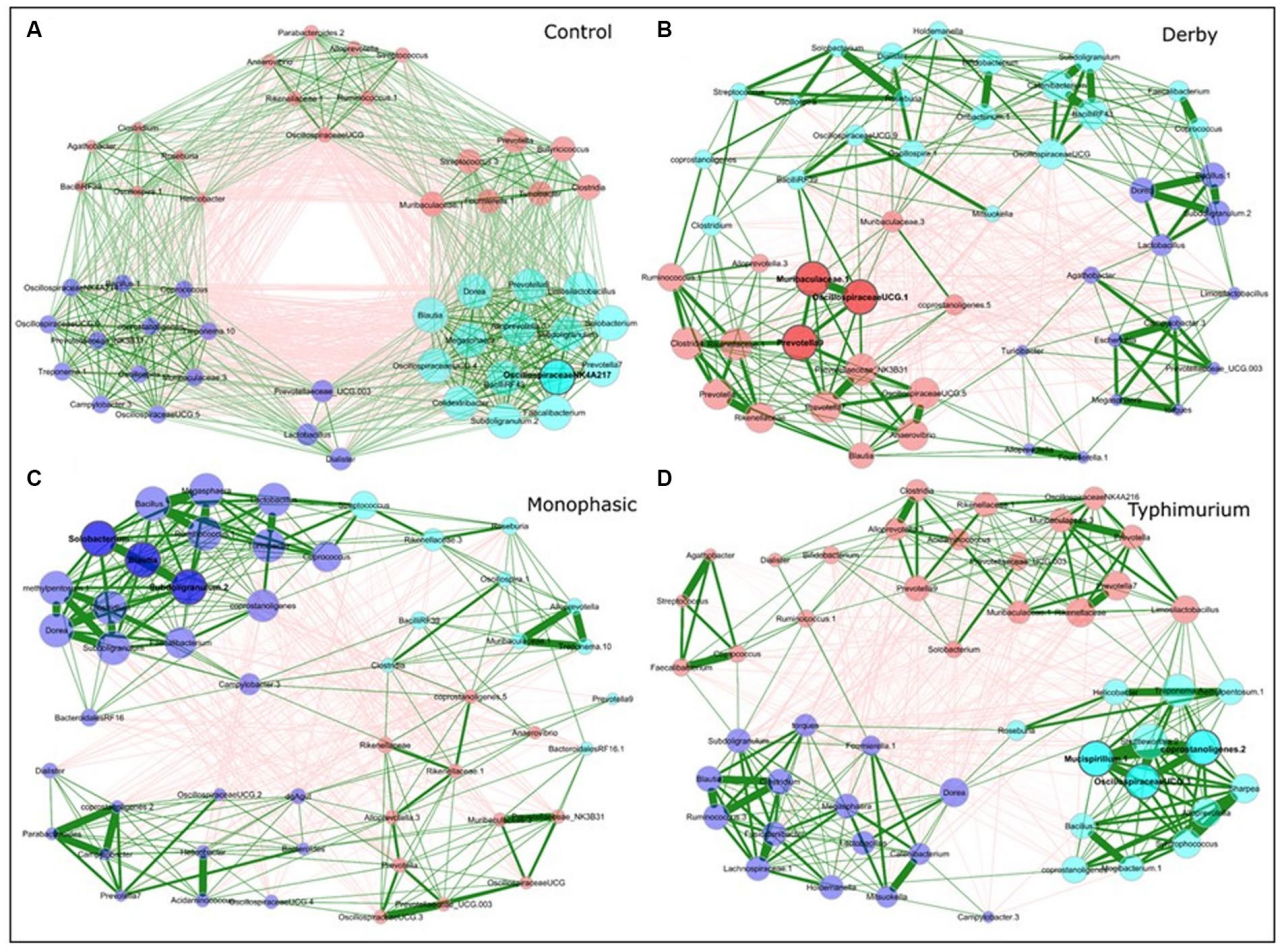
Co-occurrence network analysis for DPI 4 colonic apex microbiome across treatments (**A** - Control; **B** - Derby; **C** - Monophasic; and **D** - Typhimurium). Each node represents a bacterial taxon, and the size of each node is scaled according to its centrality. Positive Spearman-based associations between taxa are depicted as green edges (the thicker the line, the stronger the association), whereas red edges are indicative of an anti-correlation between taxa. Edges representing a value less than 0.5 are not shown. Taxa are colored based on clusters calculated in the network construction. The number of animals used per treatment can be found in the Supplementary Figure S1.

Besides examining the community structure at the beta-diversity level, co-occurrence networks were used to assess topology, interactions, central taxa, and associations within the community at DPI 2, 4, and 28, both for colonic apex and fecal samples. As minimal changes were observed in the small intestine, from here on, most of the analysis was conducted using colonic apex as the primary site of inflammation caused by *S*. Monophasic and *S.* Typhimurium, or fecal samples to examine biogeographical differences. In comparison with infected groups, control animals displayed a more organized community structure at DPI 2 and 4 for apex samples (Supplementary Figure S22 and Figures 4A–D, respectively). Additionally, the topological alteration in community architecture was more altered for *S*. Monophasic and *S.* Typhimurium during inflammation (DPI 2 and DPI 4) in comparison to DPI 28 in apex samples, but not in fecal samples (Figures 4A–D and Supplementary Figures S22–S26). Although the sample size for colonic apex samples was smaller than that of feces, it appears that community architecture was distinguishable by biogeography. A hallmark of network alteration at both central taxa and associations was that putative short chain fatty acid (SCFA) producers (e.g., *Blautia*, *Faecalibacterium*, *Alloprevotella*, *Prevotella*, *Megasphaera*, *Dorea*, etc) were more likely to be tightly clustered in the control animals during the inflammatory bottleneck (DPI 2 and 4) (Figure 4A – cyan colored group), with that signature being more distinguishable at the site of inflammation (colonic apex). This suggests these taxa mark the dysbiosis associated with infection in all serovars tested, but is accentuated in the case of *S*. Monophasic and *S.* Typhimurium due to a higher degree of inflammation. In the case of *S*. Monophasic and *S.* Typhimurium, the co-occurrence network topological alterations are mirrored by a potentially higher volatility of the microbiota at the colonic apex, when examining the PC1 pattern and variability at DPI 2, 4, and 28 (Supplementary Figure S27). Temporal cross-sectional fecal sample analysis revealed higher volatility for the microbiome of *S.* Typhimurium infected animals (variable evenness and shift in the distribution of PC1 using the Bray Curtis’s distance matrix) (Supplementary Figure S28). Altogether these results point to a dysbiosis effect in the infected animals with unique and shared signatures in community structure.

### 3.5. Direct changes in putative SCFA producers mark the inflammatory bottleneck

At first glance, taxonomic examination of individual animals across treatments and DPI 2, 4, and 28 demonstrated a predominance of the phyla Firmicutes and Bacteroidetes regardless of biogeography (Supplementary Figure S29). Upon a closer assessment at varying hierarchical levels of taxonomic resolution (Supplementary Figures S29–S34), and despite the expected individual variation, both colonic apex and fecal samples were broadly enriched with genera *Megasphaera*, *Prevotella*, *Alloprevotella*, *Clostridia*, and family *Lachnospiraceae*. Further statistical analysis based on relative abundances revealed taxa of the family *Prevotellaceae* as keystone and predominantly affected by the inflammatory bottleneck with variation between colonic apex and fecal samples when comparing to the control group and among serovars (Supplementary Figures S35–S53). Specifically, *S.* Typhimurium infected animals had on average a significantly lower proportion of the family *Prevotellaceae* (feces at DPI 2 and colonic apex at DPI 4; Supplementary Figure S35), genus *Prevotella* (feces at DPI 2; Supplementary Figure S36), *Prevotellaceae NK3B31* (feces at DPI 2; Supplementary Figure S39), *Prevotellaceae UCG 003* (feces at DPI 2 and 4), and *Fusicatenibacter* (apex at DPI 2; Supplementary Figure S46); whereas, *Megasphaera* was increased in the colonic apex at DPI 4 and feces at DPI 2 (*p* < 0.05) (Supplementary Figure S47). In the case of *S*. Monophasic, the proportion of the family *Prevotellaceae* was increased in the colonic apex at DPI 4 (Supplementary Figure S35) and was associated with a higher proportion of the genus *Alloprevotella* and *Prevotellaceae UCG 003* (*p* < 0.05) (Supplementary Figures S37, S41S1, respectively). Like *S.* Typhimurium, the genus *Fusicatenibacter* was significantly lower in proportion for *S*. Monophasic and *S*. Derby in the colonic apex at DPI 2 (*p* < 0.05) (Supplementary Figure S46).

**Figure 5:**
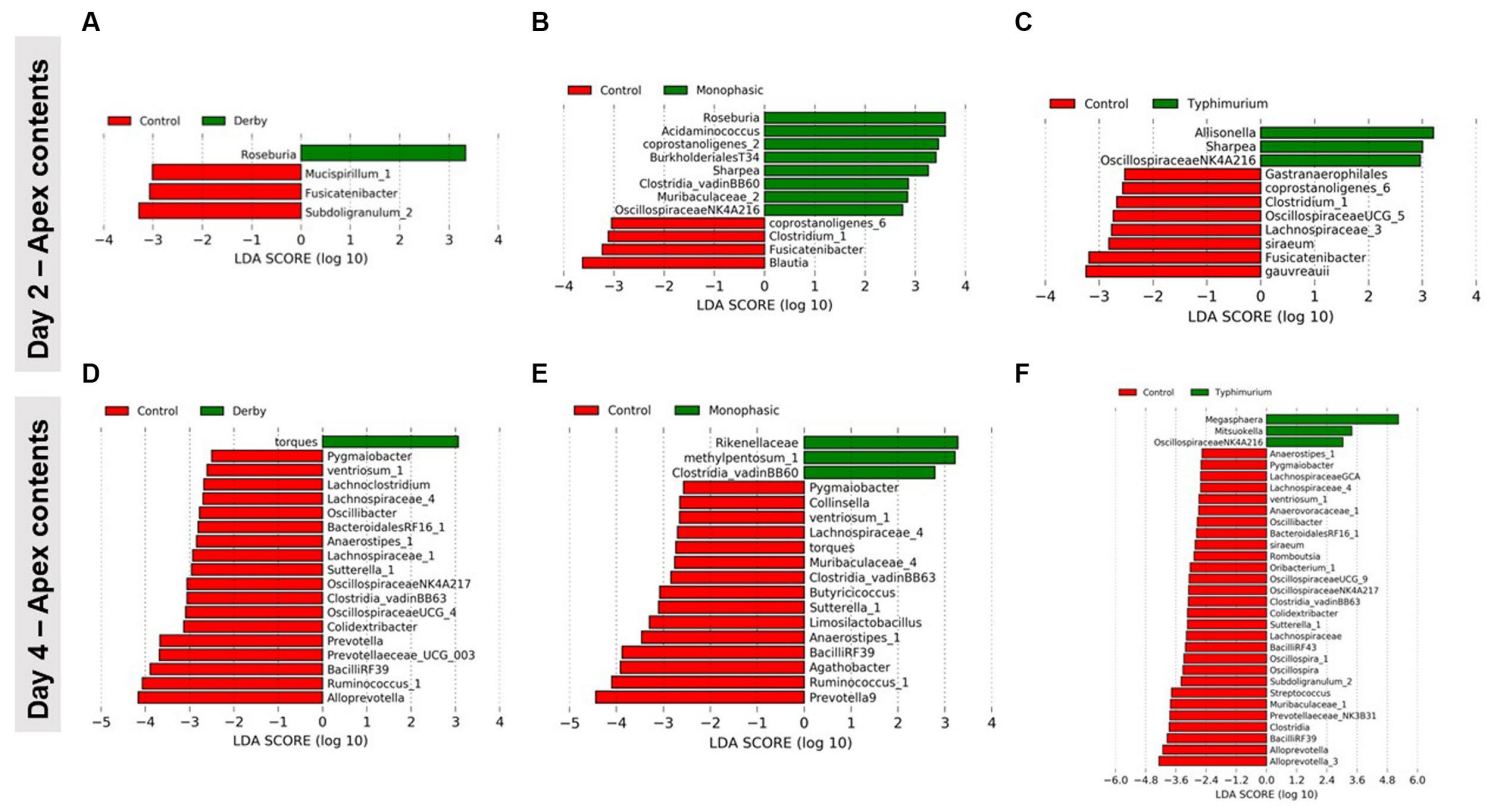
LEfSe analysis results demonstrating the most differentiating taxa between each *Salmonella*-infected group and control animals. This analysis was done for colonic apex (apex) contents only at DPI 2 **(A–C)** and 4 **(D–F)**, during the peak of the inflammatory bottleneck in the GI microbiota community structure and composition. The number of animals used per treatment can be found in the Supplementary Figure S1.

**Figure 6:**
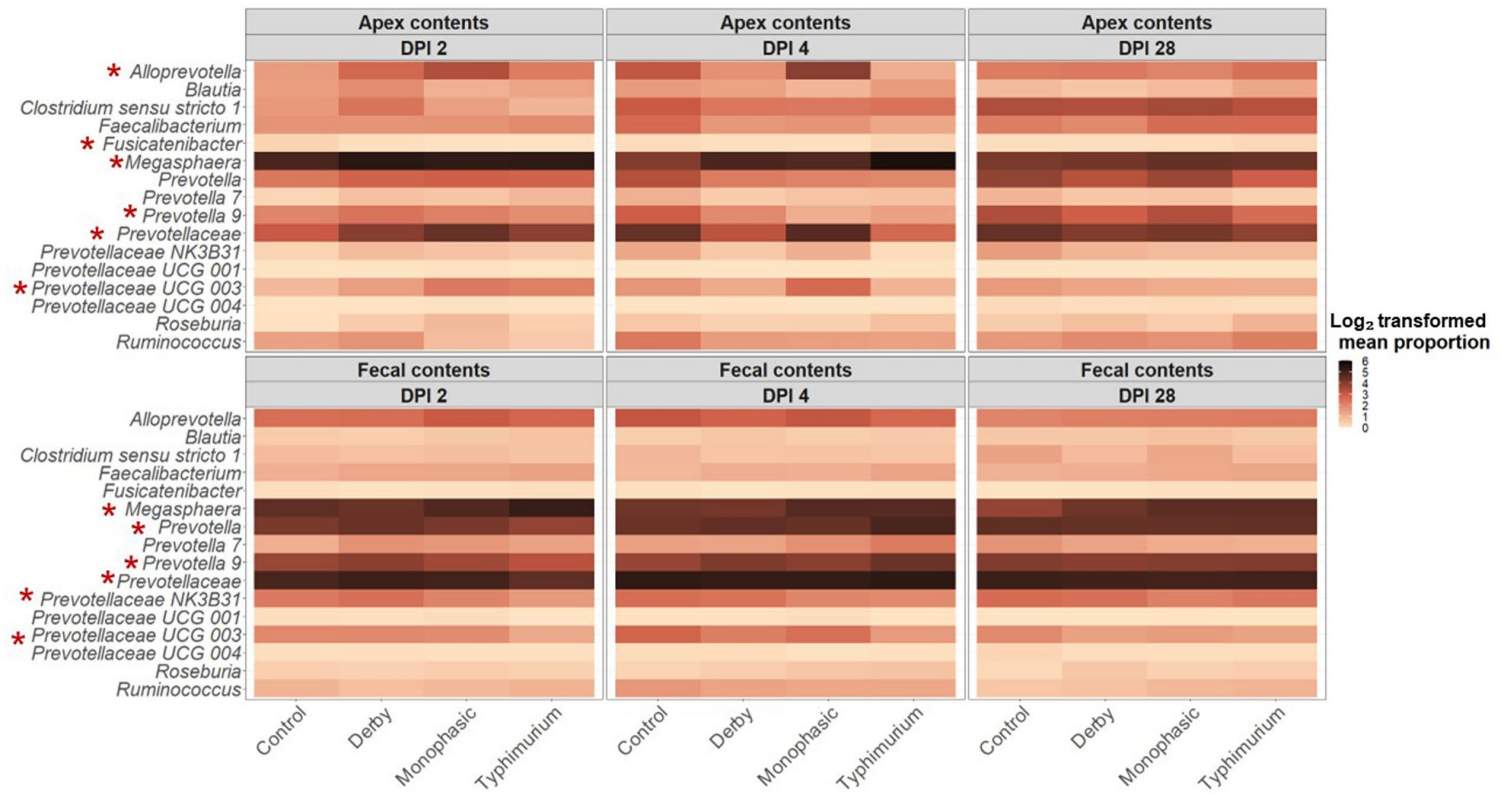
Temporal and biogeographical pattern of microbiome taxa composition across treatments (Control, Derby, Monophasic, and Typhimurium). Log_2_-transformed mean proportion of most differentiating putative SCFA bacterial producers across treatments. The stronger the color, the more abundant a given taxon is, on average, across treatments over time. Red asterisks mark taxa that were significantly different in proportion across treatments for either DPI 2 or 4, according to sample type (colonic apex vs. feces). The number of animals used per treatment can be found in the Supplementary Figure S1.

**Figure 7:**
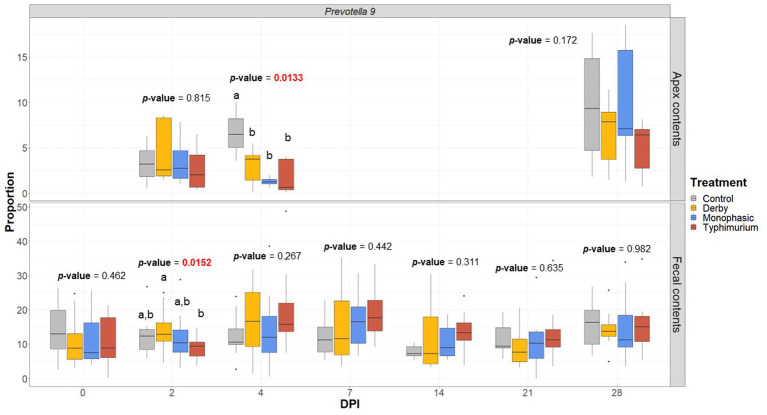
*Prevotella 9* proportion across treatments (Control, Derby, Monophasic, and Typhimurium) and DPI for both colonic apex (apex) and fecal contents. Statistical analysis was done using an ANOVA followed by a pairwise *T*-test (*p* < 0.05). Only animals that had microbiome samples passed through the bioinformatic cut-off for quality control were included in this analysis. Different superscript letters indicate significant differences between treatments. Sampling distribution across treatments and DPI can be found in Supplementary Figure S1.

LEfSe analysis further corroborated the ANOVA results for differences in proportions, and although results should be cautiously interpreted because of the small sample size, and suggested a decrease in the *Clostridia* and *Lachnospiraceae* in apex contents at DPI 2 and 4 for the infected animals (Figure 5). Figure 6 depicts the mean distribution of the most altered putative SCFA producers associated with the inflammatory bottleneck when comparing DPI and biogeography. Unique variants of the genus *Prevotella* showed a serovar-specific variation distinguishable across colonic apex and feces (e.g., *Prevotella 9* and *Prevotellacea NK3B31* with higher abundances in feces). *Prevotella 9* was found in significantly lower proportion in all infected groups at DPI 4 but only in the colonic apex (Figure 7). Other swine microbiome keystone taxa such as *Lactobacillus*, *Streptococcus*, *Campylobacter hyointestinalis*, and *Mitsuokella* were also analyzed (Supplementary Figures S50–S53). Of note, the genus *Lactobacillus* was significantly higher in *S.* Typhimurium and *Streptococcus* was higher in proportion in *S*. Derby infected animals at multiple timepoints but only in fecal samples (Supplementary Figures S50, S51) (*p* < 0.05). For ileal contents there were no significant changes in taxa proportions across treatments (*p* ≥ 0.05) except for *Alloprevotella* at DPI 4 (Supplementary Figures S54–S73). Collectively, these findings suggest that the stronger inflammatory bottleneck present in *S*. Monophasic and *S.* Typhimurium infected animals led to shared and unique changes either in the family *Prevotellaceae* or its representatives, with varying biogeographical signatures underlying community-based resistance to infection. Of note, a high resilience can be observed across all those taxa for colonic apex and fecal samples when examining the end-point sampling, although the difference in sample size must be carefully considered in the interpretation.

### 3.6. Sample biogeography impacts the interpretation of taxa abundances and associations with disease scores

**Figure 8:**
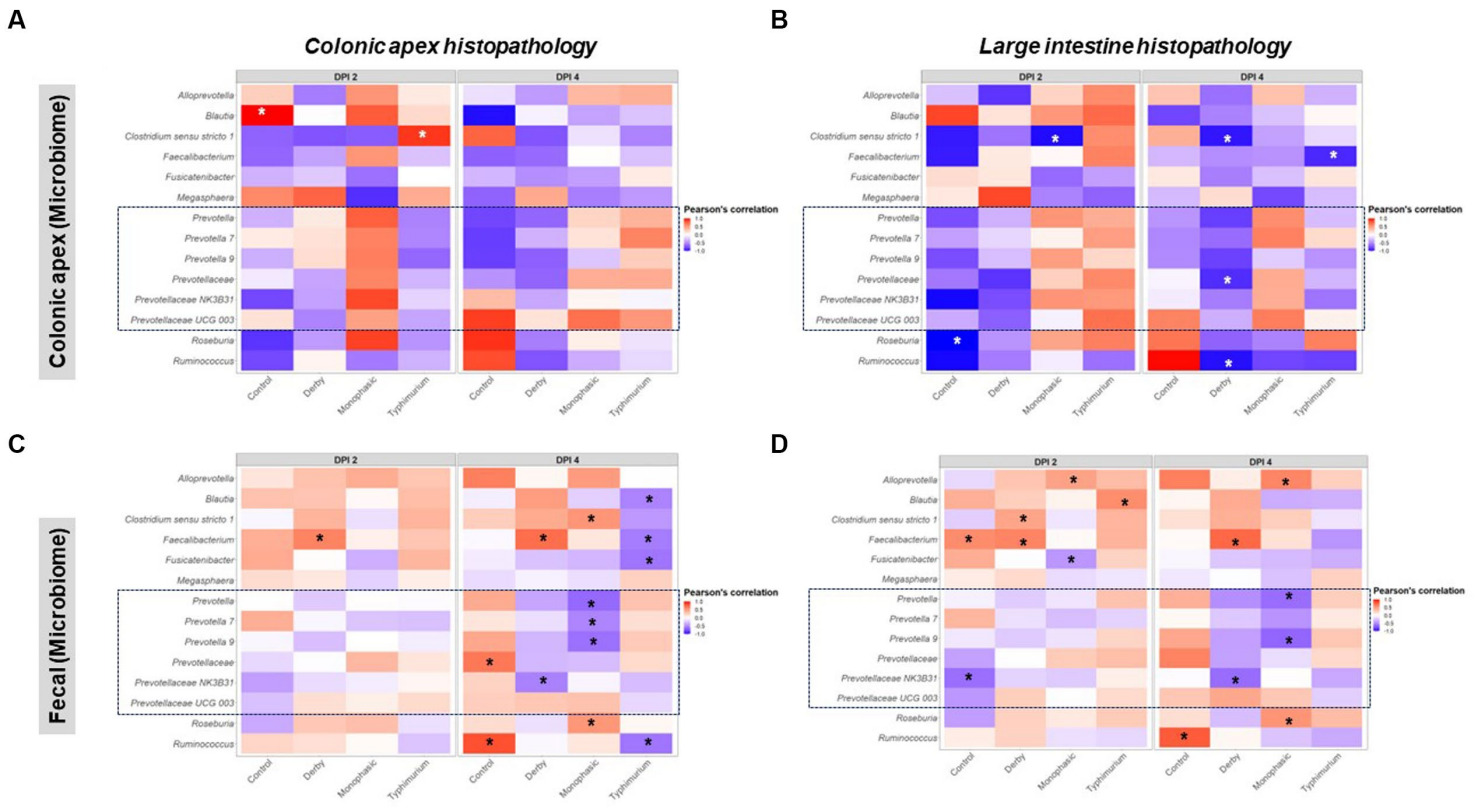
Pearson’s correlation distribution between putative SCFA taxa and disease phenotype as measured by histopathological scores in the large intestine. Plots **(A,B)** depict correlations between SCFA taxa found in the colonic apex (apex) samples with histopathological scores measured either at the apex or as an amalgamation of sections of the large intestine. Similarly, plots **(C,D)** represent the same correlations but now using taxonomic distributions found in the fecal contents. All correlations were calculated using the log_2_ transformation of the total proportion calculated per taxon. The number of animals per treatment can be found in the Supplementary Figure S1. Asterisks are only present in taxa that passed the statistical significance cut-off (*p* < 0.05).

As putative SCFA producing bacteria were a mark of *Salmonella*-induced transient dysbiosis and low community-based resistance, the next step in the analysis was to correlate their proportion with histopathological scores, which was used as a proxy for the severity of niche-specific inflammation. Histopathological alterations were assessed either for the colonic apex alone or cumulatively for the large intestine as previously reported (8) and disease associations were drawn from taxa proportion both at the colonic apex and feces to identify unique and shared signatures with putative SCFA producing bacteria. Overall, stronger Pearson’s correlation values were found when comparing colonic apex vs. fecal microbiome as data input at the peak of inflammation (DPI 2 and 4) (Figures 8A–D). For the colonic apex microbiome, significant anti-correlations with disease scores were only found when using the cumulative large intestine score at DPI 4 (Figures 8A,B), whereas negative correlations were more readily detectable with fecal microbiome for both *S*. Monophasic and *S.* Typhimurium (*p* < 0.05) (Figures 8C,D). Data interpretation specifically for colonic apex is limited by the small sample size at DPI 2 and 4. However, at DPI 4, for both *S*. Monophasic and *S.* Typhimurium, there was an overall pattern of negative correlation between non-*Prevotella* putative SCFA producers more consistently quantified at the site of inflammation (Figures 8A,B) rather than with fecal microbiota samples (Figures 8C,D). In the case of members of the *Prevotellaceae* family, specifically at DPI 4, negative correlations with disease scores could only be found for *S*. Monophasic infected animals when using fecal microbiome (Figures 8C,D). For *S.* Typhimurium, the association with *Prevotellaceae* members varied across biogeography and scoring system (Figures 8A–D). In the case of *S*. Derby, *Prevotellaceae* taxa were often found to be anti-correlated with the disease phenotype, albeit significant values were limited (*p* < 0.05). Overall, putative SCFA producing bacteria were confirmed to be a hallmark of the *Salmonella*-induced dysbiosis (inflammatory bottleneck), but with variable patterns depending on biogeography and disease scoring system. Finally, despite direct inoculation, induction of clinical disease, and culture of *Salmonella* from feces and intestinal contents, changes in the prevalence of *Enterobacterales* were not noted (Supplementary Figure S74) and overall abundance was low.

## 4. Discussion

Despite significant investments in diagnostics, surveillance, vaccination, use of antibiotics and antibiotic alternatives (e.g., feed additives), salmonellosis remains a significant concern for both swine and human medicine, especially with the emergence of multi-drug resistant isolates among the serovars tested here (22, 24, 28, 49–52). In regards to dietary based strategies, given that colonization resistance to multiple serovars may not happen through a single mechanism driven by one microorganism, there remains to be understood which bacterial species, and lineages within a species, would most effectively work as host-adapted probiotics to decrease the prevalence of *Salmonella* at the farm level (32). The main objective of this study was to map at the 16S rRNA level of resolution the specific kinetics of GI microbiome taxa changes across the small and large intestine at acute and recovery stages of inflammation across three major *Salmonella* serovars (Derby, Monophasic, and Typhimurium) known to reside in GI tract of pigs. By assessing GI microbiome changes across different anatomic sites, serovars, and multiple DPI, we were able to identify specific dysbiotic signatures related to low resistance to the inflammatory perturbation caused by *Salmonella* infection. Specifically, we identified both shared and serovar specific signatures previously demonstrated to be associated with SCFA production which were most clearly observed at the site of inflammation (i.e., – large intestine) and feces.

As with other mammals, the swine microbiome has biogeographically distinct compositional features. In a meta-analysis, Holman et al. (36) demonstrated that at the Phylum level, the gastric and small intestinal microbiome were mostly comprised of Firmicutes, whereas at the large intestinal level, it was a mixed composition of predominantly Bacteroidetes and Firmicutes particularly enriched with *Prevotella* (36). The same authors also concluded that a fraction of the microbiota is shared across a minimum of 90% of the GI samples, including *Clostridium*, *Blautia*, *Lactobacillus*, *Prevotella*, *Ruminococcus*, *Roseburia*, and others (36). More recent work has corroborated some of these findings in highlighting the *Clostridiales* and *Prevotella* as part of the core microbiome of piglets aged 21–35 days (53, 54); this is consistent with our findings on DPI 0 (pre-inoculation). However, recent work by Luo et al. (37) demonstrated high variability in the proportion of *Prevotellaceae*/*Prevotella* post-weaning, potentially creating multiple enterotypes in the swine population (37). Variability in enterotypes could contribute to the variability of community-based resistance to *Salmonella*-induced inflammation as suggested in our study. This pre- and post-weaning window of time is particularly important in production systems since it is predicted to be an increased time of susceptibility to *Salmonella* colonization as *S.* Typhimurium has been shown to exploit the host inflammatory milieu during this window to gain trans-intestinal epithelial access and persist in swine (8, 32, 38, 55, 56). Based on murine models, it has been further demonstrated that *S.* Typhimurium elicits host inflammation, which by consequence alters the oxygen micro-environment (increased aerobiosis) near the epithelium, and shrinks the population and diversity of oxygen-sensitive bacterial such as *Clostridiales*, and likely other SCFA producers that rely on anaerobic metabolism, therefore facilitating pathogen blooming (38, 39, 57). Under physiological conditions, SCFAs such as butyrate can be used as an energy source for epithelial cells via aerobic metabolism. In a dysbiotic micro-environment (e.g., site of inflammation such as colonic apex), the lower concentrations of SCFAs are predicted to lead to a shift in metabolism by epithelial cells, which now consume more glucose as their main energy source, and thereby increasing the oxygen and nitric oxide concentration resulting in *Enterobacterales* expansion (e.g., *S.* Typhimurium) (39, 58, 59). The decomposition of this complex trait, namely colonization resistance, suggests that the presence of both SCFA producers and beneficial oxygen consumers could alter the shedding and persistence of *S.* Typhimurium by increasing community-based resistance and resilience over time. It has been recently demonstrated that there is an anti-correlation between *Prevotella* (a keystone taxon in the GI tract of pigs) relative abundance and fecal shedding of both *S*. Monophasic and *S.* Typhimurium in pigs (33, 34). *Prevotella* have been associated with increased SCFA production although the exact SCFA produced by species that inhabit the swine GI tract has not been well characterized (34). Although lacking the mechanistic basis, our work further suggests that the enrichment of the GI microbiome with bacteria that can alter SCFA production may have a pleiotropic effect in diminishing intestinal pathology.

As previously reported, the mean amount of *Salmonella* shed in the feces of the animals in our study peaked at approximately 3.0 log_10_ CFU/mL on DPI 2 to 4 for all serovars, after which shedding steadily decreased and was not detectable in all but two *S.* Typhimurium-infected animals by DPI 28 (8). Despite direct inoculation, culture of viable organisms from multiple sites, and the demonstrated ability to induce clinical disease in swine for two of the three serovars utilized, detection of *Salmonella* itself was not noted using the 16S methodology employed here in either the fecal samples or the intestinal samples collected at necropsy. The V4 region of 16S rRNA has been previously shown to have lower resolution between genera (60), therefore it is likely that reads associated with *Salmonella* were assigned to other members the *Enterobacteriacae*, however, significant changes within this taxon were also not noted for any DPI or sample type. This indicates that subtle changes in the microbiome through introduction of pathogenic organisms can have significant impacts on the health of animals and disease carriage status. While clinical disease was only present in the *S.* Typhimurium and *S*. Monophasic groups and not the *S*. Derby group, our study further suggests that *Prevotella* may have a pleiotropic impact in colonization resistance against *S*. Derby, *S*. Monophasic, and *S.* Typhimurium, albeit varying *Prevotellaceae* types (ASV) had distinguishable biogeographical effect in both *S*. Monophasic and *S.* Typhimurium. From both our study and previous studies, it remains unclear which specific *Prevotella* species might have distinguishable colonization resistance properties; this limitation is again in part due to the inherent issues with 16S rRNA analysis, which does not provide enough resolution for accurate species-level classification (35, 61). Considering that *S*. Monophasic and *S.* Typhimurium infection and inflammation can be compartmentalized to specific segments of the large intestine (e.g., cecum and apex colonic tissues) (8), further studies are needed to assess the composition of the microbial communities in those specific anatomical sites, including a comparison of luminal to mucosal-associated communities (which was attempted here but failed likely due to low yield and contamination with host DNA), use of deeper sequencing techniques (i.e., – shotgun sequencing), and the isolation and whole-genome sequencing of autochthonous *Prevotella* species with demonstrable *in vivo* probiotic properties (35). As our study was limited in statistical power and did not measure SCFA concentrations, subsequent efforts could be made to have more robust absolute quantification of potentially protective microorganisms and metabolites at both the site of infection as well as in the feces.

Provided specific species of *Prevotella* sp. are found to have consistent anti-carriage properties against *S*. Monophasic and *S.* Typhimurium, bacterial genetics/genomics combined with *in vivo* animal experimentation could allow for probing of unique metabolic traits that could be harnessed to enrich for such species using dietary changes on farms (32, 35). Among the known species, *Prevotella copri* and *P. stercorea* are the most likely candidate species to have a protective effect on *Salmonella* infection in swine (35). As a proof-of-concept, Trachsel et al. (33) recently demonstrated that the incorporation of resistant potato starch in the diet of swine to enrich for species of interest (e.g., *Prevotella* sp.) at the site of infection could aid in reducing the carriage of multi-drug resistant *S*. Monophasic; direct feeding of fatty acids associated with disease resistance did not produce a positive effect (33). Additional studies have corroborated the hypothesis that the utilization of resistant starch can directly alter the swine gut microbiome composition through the enrichment of SCFA bacteria such as *Prevotella-, Lachnospiraceae*- and *Ruminococcus*-associated phylotypes (62, 63). The utilization of highly fermentable fibers may even have a broader effect on gut health since it is beneficial against another GI swine pathogen, *Brachyspira hyodysenteriae* (64). Alternatively, corn bran may be a fiber source capable of modulating the swine GI microbiome by inducing SCFA microbes which would be hypothesized to have positive effects in terms of reducing *S. enterica* shedding in pigs (65). Of note, the potential expansion of *Alloprevotella* in *S*. Monophasic vs. *Megasphaera* in *S.* Typhimurium infected pigs observed here may be associated with the utilization of lactate by these microbes (66, 67), which is a host derived metabolite in *S.* Typhimurium infection in a murine model (68, 69). Directly feeding fatty acids to swine as a preventive measure can be challenging due to variable effects of different molecules, identification of effective biological concentrations without altering diet palatability, varying GI absorption and excretion, potential unpredictable effects on other beneficial microbial community members, and influence of the host immune system (70). Thus, feeding strategies with ingredients such as resistant potato starch that directly enrich for species of interest (e.g., *Prevotella* sp.) at the site of inflammation (33), or the direct incorporation of commercially viable probiotics with the desired properties, are more likely to be effective in enhancing colonization resistance and decreasing carriage of *Salmonella* in swine farms. Depending on the mix of probiotics (e.g., *Bacillus amyloliquefaciens* G10, *Levilactobacillus brevis* M10, and *Limosilactobacillus reuteri* RTR), there can be a reduction in the relative abundance of SCFA producers in the GI tract of pigs (66), which would be hypothesized to decrease colonization resistance against the serovars tested here. Additionally, it is not known how *Prevotella* strain variation can influence the assembly of pig large intestine microbiome, and by consequence impact the variance in colonization resistance against *Salmonella*. Such genetic diversity has been reported to exist in *P. copri* isolated from humans and is predicted to have been impacted by diet with the lowering in fiber content being a potential detrimental factor for engraftment of the GI microbiome (71). Also, it has been recently reported that distinct *P. copri* isolates may better utilize certain dietary polysaccharides which can become a basis for the studying of new symbiotic approaches (72). A cautionary note is that not all *Prevotella* may have beneficial effects but might under specific host genetic susceptibility behave as a pathobiont (73). Other potential confounding factors such as the swine GI mycobiome may also influence the ability of *Prevotella* to decrease the carriage of *Salmonella* as recent studies from Summers et al. point to the existence of an autochthonous beneficial fungi in the swine large intestine, *Kazachstania slooffiae*, that is positively correlated with *Prevotella* sp. abundance (53, 54, 74, 75). Therefore, another component of this complex trait, namely colonization resistance against *Salmonella* in pigs, might be partially mediated by a mutualistic interaction between bacteria and yeast in the gastrointestinal tract.

Despite including multiple serovars in this study in an attempt to assess the effect of different serovars on microbiome composition, it is known that the population of *S*. Derby, *S*. Monophasic, and *S.* Typhimurium are genetically diverse both at the sequence type (ST) and cgMLST levels (4, 11, 12, 16, 50, 76). Each serovar has a high degree of clonality at the ST level (very few dominant STs distributed worldwide), and isolates were chosen from the dominant ST groups to best enable comparisons (Typhimurium, ST19; Monophasic, ST34, Derby, ST40) (8); in the future additional experimental approaches should be employed to account for phylogenetic diversity and variation in zoonotic potential (4, 12). Use of a combination of STs of a given serovar to assess the colonization resistance properties of the GI microbiome or a specific cocktail of candidate probiotics would more robustly determine their efficacy. The more STs and cgMLSTs that are included in each serovar-specific cocktail to be tested, the more likely the probiotic or interventional strategy is to be generalizable at the farm level. Additionally, variation in serovar-specific antimicrobial resistant (AMR) patterns within population structures (ST and cgMLST) could be included in assessing microbiome-based interventions that could potentially be applied to AMR lineages.

In summary, this study demonstrates the importance of studying the biogeographic changes of the GI microbiome to harness information regarding specific properties of the microbiome that can aid in colonization resistance against zoonotic serovars of *S. enterica* lineage I that reside in swine. It also underscores the fact that fecal monitoring of the GI microbiome diversity or taxa may not always be predictive of colonization resistance. Lastly, our work highlights a dysbiotic signature of *Salmonella*-infected pigs primarily altering the composition and proportion of putative SCFA producers that was linked to the inflammatory bottleneck created by the pathogen. Of such, *Prevotella* was identified as a keystone taxa in the swine GI microbiome that appears to be a biomarker with potential to have an impact on the disease phenotype perhaps as a central taxon in enhancing community-based resistance to infection. Therefore, our work, in conjunction with others, points toward a niche-specific effect related to microbiome changes at the site of infection; this information can be used to inform the future development of probiotic, prebiotic, or symbiotic strategies for scalable controlling of *Salmonella* in farms at both a narrow (serovar-specific) or broad spectrum level.

## Data availability statement

The datasets presented in this study can be found in online repositories. The names of the repository/repositories and accession number(s) can be found at: https://www.ncbi.nlm.nih.gov/bioproject/944612.

## Ethics statement

The animal studies were approved by Iowa State University Institutional Animal Care and Use Committee. The studies were conducted in accordance with the local legislation and institutional requirements. Written informed consent was not obtained from the owners for the participation of their animals in this study because the animals were research animals owned by the university therefore informed consent was not applicable.

## Author contributions

AK and BA conceived and designed the project. SN was responsible for performing the experimental studies with assistance from AK, BA, and others (see “Acknowledgements”). SN prepared samples for metagenomic analysis and BA performed the histopathology assessments. JCG-N, NP, and AK conducted all metagenomic data analysis and generated all data visualizations. NP carried out all bioinformatics work. JCG-N, NP, and AK wrote the initial manuscript. NK and AB helped with data interpretation and final manuscript preparation. NK led the network and LEfSe analysis including figure preparation and data interpretation. All authors contributed to the article and approved the submitted version.

## Funding

This work was supported in part by funding provided by the National Pork Board (grant #16-215: Investigation of pathogenicity, competitive fitness, and novel methods for rapid diagnosis of *S*. 4,[5],12:i:-).

## Conflict of interest

The authors declare that the research was conducted in the absence of any commercial or financial relationships that could be construed as a potential conflict of interest.

## Publisher’s note

All claims expressed in this article are solely those of the authors and do not necessarily represent those of their affiliated organizations, or those of the publisher, the editors and the reviewers. Any product that may be evaluated in this article, or claim that may be made by its manufacturer, is not guaranteed or endorsed by the publisher.

## Footnotes

^1^ https://hcc.unl.edu/

## References

[1] CDC. (2021). Food safety. Available at: https://www.cdc.gov/foodsafety/foodborne-germs.html.

[2] CDC. (2021). *Salmonella*. Available at: https://www.cdc.gov/salmonella/.

[3] Ferrari RG Rosario DKA Cunha-Neto A Mano SB Figueiredo EES Conte-Junior CA. Worldwide epidemiology of *Salmonella* serovars in animal-based foods: a meta-analysis. Appl Environ Microbiol. (2019) 85:e00591–19. doi: 10.1128/AEM.00591-19, PMID: 31053586 PMC6606869

[4] Achtman M Wain J Weill F-X Nair S Zhou Z Sangal V. Multilocus sequence typing as a replacement for serotyping in *Salmonella enterica*. PLoS Pathog. (2012) 8:e1002776. doi: 10.1371/journal.ppat.1002776, PMID: 22737074 PMC3380943

[5] Hauser E Hebner F Tietze E Helmuth R Junker E Prager R. Diversity of *Salmonella enterica* serovar Derby isolated from pig, pork and humans in Germany. Int J Food Microbiol. (2011) 151:141–9. doi: 10.1016/j.ijfoodmicro.2011.08.020, PMID: 21917347

[6] Kerouanton A Rose V Weill F-X Granier SA Denis M. Genetic diversity and antimicrobial resistance profiles of *Salmonella enterica* serotype Derby isolated from pigs, pork, and humans in France. Foodborne Pathog Dis. (2013) 10:977–84. doi: 10.1089/fpd.2013.1537, PMID: 23944749

[7] Sanchez J Dohoo IR Christensen J Rajic A. Factors influencing the prevalence of *Salmonella* spp. in swine farms: a meta-analysis approach. Prev Vet Med. (2007) 81:148–77. doi: 10.1016/j.prevetmed.2007.04.005, PMID: 17498826

[8] Naberhaus SA Krull AC Arruda BL Arruda P Sahin O Schwartz KJ. Pathogenicity and competitive fitness of *Salmonella enterica* serovar 4,[5],12:i:- compared to *Salmonella* Typhimurium and *Salmonella* Derby in swine. Front Vet Sci. (2020) 6:502. doi: 10.3389/fvets.2019.00502, PMID: 32083096 PMC7002397

[9] Peckham CF Savage WG. An outbreak of pork pie poisoning at Derby. J Hyg. (1923) 22:69–76. doi: 10.1017/S0022172400008068, PMID: 20474799 PMC2167419

[10] Cui S Li J Sun Z Hu C Jin S Li F. Characterization of *Salmonella enterica* isolates from infants and toddlers in Wuhan, China. J Antimicrob Chemother. (2008) 63:87–94. doi: 10.1093/jac/dkn452 18984647

[11] Gomes-Neto JC Pavlovikj N Cano C Abdalhamid B Al-Ghalith GA Loy JD. Heuristic and hierarchical-based population mining of *Salmonella enterica* lineage I pan-genomes as a platform to enhance food safety. Front Sustain Food Syst. (2021) 5:725791. doi: 10.3389/fsufs.2021.725791

[12] Alikhan N-F Zhou Z Sergeant MJ Achtman M. A genomic overview of the population structure of *Salmonella*. PLoS Genet. (2018) 14:e1007261. doi: 10.1371/journal.pgen.1007261, PMID: 29621240 PMC5886390

[13] Sun H Wan Y Du P Bai L. The epidemiology of monophasic *Salmonella* Typhimurium. Foodborne Pathog Dis. (2020) 17:87–97. doi: 10.1089/fpd.2019.2676 31532231

[14] Moreno Switt AI Soyer Y Warnick LD Wiedmann M. Emergence, distribution, and molecular and phenotypic characteristics of *Salmonella enterica* serotype 4,5,12:i:-. Foodborne Pathog Dis. (2009) 6:407–15. doi: 10.1089/fpd.2008.0213, PMID: 19292687 PMC3186709

[15] Arai N Sekizuka T Tamamura Y Kusumoto M Hinenoya A Yamasaki S. *Salmonella* genomic island 3 is an integrative and conjugative element and contributes to copper and arsenic tolerance of *Salmonella enterica*. Antimicrob Agents Chemother. (2019) 63:e00429–19. doi: 10.1128/AAC.00429-19, PMID: 31209002 PMC6709492

[16] Branchu P Charity OJ Bawn M Thilliez G Dallman TJ Petrovska L. SGI-4 in monophasic *Salmonella* Typhimurium ST34 is a novel ICE that enhances resistance to copper. Front Microbiol. (2019) 10:1118. doi: 10.3389/fmicb.2019.01118, PMID: 31178839 PMC6543542

[17] Clark CG Landgraff C Robertson J Pollari F Parker S Nadon C. Distribution of heavy metal resistance elements in Canadian *Salmonella* 4,[5],12:i:- populations and association with the monophasic genotypes and phenotype. PLoS One. (2020) 15:e0236436. doi: 10.1371/journal.pone.0236436, PMID: 32716946 PMC7384650

[18] Mourão J Novais C Machado J Peixe L Antunes P. Metal tolerance in emerging clinically relevant multidrug-resistant *Salmonella enterica* serotype 4,[5],12:i:− clones circulating in Europe. Int J Antimicrob Agents. (2015) 45:610–6. doi: 10.1016/j.ijantimicag.2015.01.013 25816978

[19] Bearson BL Trachsel JM Shippy DC Sivasankaran SK Kerr BJ Loving CL. The role of *Salmonella* genomic island 4 in metal tolerance of *Salmonella enterica* serovar I 4,[5],12:i:- pork outbreak isolate USDA15WA-1. Genes. (2020) 11:1291. doi: 10.3390/genes11111291, PMID: 33142960 PMC7716197

[20] He J Sun F Sun D Wang Z Jin S Pan Z. Multidrug resistance and prevalence of quinolone resistance genes of *Salmonella enterica* serotypes 4,[5],12:i:- in China. Int J Food Microbiol. (2020) 330:108692. doi: 10.1016/j.ijfoodmicro.2020.108692, PMID: 32521291

[21] Ingle DJ Ambrose RL Baines SL Duchene S Gonçalves da Silva A Lee DYJ. Evolutionary dynamics of multidrug resistant *Salmonella enterica* serovar 4,[5],12:i:- in Australia. Nat Commun. (2021) 12:4786. doi: 10.1038/s41467-021-25073-w, PMID: 34373455 PMC8352879

[22] Long L You L Wang D Wang M Wang J Bai G. Highly prevalent MDR, frequently carrying virulence genes and antimicrobial resistance genes in *Salmonella enterica* serovar 4,[5],12:i:- isolates from Guizhou Province, China. PLoS One. (2022) 17:e0266443. doi: 10.1371/journal.pone.0266443, PMID: 35588421 PMC9119451

[23] Pires J Huisman JS Bonhoeffer S Van Boeckel TP. Multidrug resistance dynamics in *Salmonella* in food animals in the United States: an analysis of genomes from public databases. Microbiol Spectr. (2021) 9:e00495–21. doi: 10.1128/Spectrum.00495-21, PMID: 34704804 PMC8549754

[24] Qin X Yang M Cai H Liu Y Gorris L Aslam MZ. Antibiotic resistance of *Salmonella* Typhimurium monophasic variant 1,4,[5],12:i:-in China: a systematic review and meta-analysis. Antibiotics. (2022) 11:532. doi: 10.3390/antibiotics11040532, PMID: 35453283 PMC9031511

[25] Berge AC Wierup M. Nutritional strategies to combat *Salmonella* in mono-gastric food animal production. Animal. (2012) 6:557–64. doi: 10.1017/S1751731111002217, PMID: 22436270

[26] de la Cruz ML Conrado I Nault A Perez A Dominguez L Alvarez J. Vaccination as a control strategy against *Salmonella* infection in pigs: a systematic review and meta-analysis of the literature. Res Vet Sci. (2017) 114:86–94. doi: 10.1016/j.rvsc.2017.03.005, PMID: 28340428

[27] Young I Wilhelm BJ Cahill S Nakagawa R Desmarchelier P Rajić A. A rapid systematic review and meta-analysis of the efficacy of slaughter and processing interventions to control nontyphoidal *Salmonella* in beef and pork. J Food Prot. (2016) 79:2196–210. doi: 10.4315/0362-028X.JFP-16-203, PMID: 28104927 PMC5238939

[28] Bearson SMD. *Salmonella* in swine: prevalence, multidrug resistance, and vaccination strategies. Annu Rev Anim Biosci. (2022) 10:373–93. doi: 10.1146/annurev-animal-013120-043304, PMID: 34699256

[29] Davies PR. Intensive swine production and pork safety. Foodborne Pathog Dis. (2011) 8:189–201. doi: 10.1089/fpd.2010.0717, PMID: 21117987

[30] Ojha S Kostrzynska M. Approaches for reducing *Salmonella* in pork production. J Food Prot. (2007) 70:2676–94. doi: 10.4315/0362-028X-70.11.2676 18044456

[31] Helke KL McCrackin MA Galloway AM Poole AZ Salgado CD Marriott BP. Effects of antimicrobial use in agricultural animals on drug-resistant foodborne salmonellosis in humans: a systematic literature review. Crit Rev Food Sci Nutr. (2017) 57:472–88. doi: 10.1080/10408398.2016.1230088, PMID: 27602884

[32] Kim HB Isaacson RE. *Salmonella* in swine: microbiota interactions. Annu Rev Anim Biosci. (2017) 5:43–63. doi: 10.1146/annurev-animal-022516-022834 27860494

[33] Trachsel JM Bearson BL Kerr BJ Shippy DC Byrne KA Loving CL. Short chain fatty acids and bacterial taxa associated with reduced *Salmonella enterica* serovar I 4,[5],12:i:- shedding in swine fed a diet supplemented with resistant potato starch. Microbiol Spectr. (2022) 10:e02202–21. doi: 10.1128/spectrum.02202-21, PMID: 35532355 PMC9241843

[34] Bearson SMD Allen HK Bearson BL Looft T Brunelle BW Kich JD. Profiling the gastrointestinal microbiota in response to *Salmonella*: low versus high *Salmonella* shedding in the natural porcine host. Infect Genet Evol. (2013) 16:330–40. doi: 10.1016/j.meegid.2013.03.022 23535116

[35] Amat S Lantz H Munyaka PM Willing BP. Prevotella in pigs: the positive and negative associations with production and health. Microorganisms. (2020) 8:1584. doi: 10.3390/microorganisms8101584, PMID: 33066697 PMC7602465

[36] Holman DB Brunelle BW Trachsel J Allen HK. Meta-analysis to define a core microbiota in the swine gut. mSystems. (2017) 2:e00004–17. doi: 10.1128/mSystems.00004-17 28567446 PMC5443231

[37] Luo Y Ren W Smidt H Wright A-DG Yu B Schyns G. Dynamic distribution of gut microbiota in pigs at different growth stages: composition and contribution. Microbiol Spectr. (2022) 10:e00688:e0068821. doi: 10.1128/spectrum.00688-21, PMID: 35583332 PMC9241710

[38] Bescucci DM Moote PE Ortega Polo R Uwiera RRE Inglis GD. *Salmonella enterica* serovar Typhimurium temporally modulates the enteric microbiota and host responses to overcome colonization resistance in swine. Appl Environ Microbiol. (2020) 86:e01569–20. doi: 10.1128/AEM.01569-20, PMID: 32859592 PMC7580545

[39] Olsan EE Byndloss MX Faber F Rivera-Chávez F Tsolis RM Bäumler AJ. Colonization resistance: the deconvolution of a complex trait. J Biol Chem. (2017) 292:8577–81. doi: 10.1074/jbc.R116.752295, PMID: 28389556 PMC5448087

[40] Apprill A McNally S Parsons R Weber L. Minor revision to V4 region SSU rRNA 806R gene primer greatly increases detection of SAR11 bacterioplankton. Aquat Microb Ecol. (2015) 75:129–37. doi: 10.3354/ame01753

[41] Caporaso JG Lauber CL Walters WA Berg-Lyons D Lozupone CA Turnbaugh PJ. Global patterns of 16S rRNA diversity at a depth of millions of sequences per sample. Proc Natl Acad Sci U S A. (2011) 108:4516–22. doi: 10.1073/pnas.1000080107, PMID: 20534432 PMC3063599

[42] Parada AE Needham DM Fuhrman JA. Every base matters: assessing small subunit rRNA primers for marine microbiomes with mock communities, time series and global field samples: primers for marine microbiome studies. Environ Microbiol. (2016) 18:1403–14. doi: 10.1111/1462-2920.13023, PMID: 26271760

[43] Matsuki T Watanabe K Fujimoto J Takada T Tanaka R. Use of 16S rRNA gene-targeted group-specific primers for real-time PCR analysis of predominant bacteria in human feces. Appl Environ Microbiol. (2004) 70:7220–8. doi: 10.1128/AEM.70.12.7220-7228.2004, PMID: 15574920 PMC535136

[44] Bolyen E Rideout JR Dillon MR Bokulich NA Abnet CC Al-Ghalith GA. Reproducible, interactive, scalable and extensible microbiome data science using QIIME 2. Nat Biotechnol. (2019) 37:852–7. doi: 10.1038/s41587-019-0209-9, PMID: 31341288 PMC7015180

[45] Callahan BJ McMurdie PJ Rosen MJ Han AW Johnson AJA Holmes SP. DADA2: high-resolution sample inference from Illumina amplicon data. Nat Methods. (2016) 13:581–3. doi: 10.1038/nmeth.3869, PMID: 27214047 PMC4927377

[46] Segata N Izard J Waldron L Gevers D Miropolsky L Garrett WS. Metagenomic biomarker discovery and explanation. Genome Biol. (2011) 12:R60. doi: 10.1186/gb-2011-12-6-r60, PMID: 21702898 PMC3218848

[47] The Galaxy Community Afgan E Nekrutenko A Grüning BA Blankenberg D Goecks J. The Galaxy platform for accessible, reproducible and collaborative biomedical analyses: 2022 update. Nucleic Acids Res. (2022) 50:W345–51. doi: 10.1093/nar/gkac247, PMID: 35446428 PMC9252830

[48] Peschel S Müller CL von Mutius E Boulesteix A-L Depner M. NetCoMi: network construction and comparison for microbiome data in R. Brief Bioinform. (2021) 22:bbaa290. doi: 10.1093/bib/bbaa290, PMID: 33264391 PMC8293835

[49] Sun R-Y Ke B-X Fang L-X Guo W-Y Li X-P Yu Y. Global clonal spread of mcr-3-carrying MDR ST34 *Salmonella enterica* serotype Typhimurium and monophasic 1,4,[5],12:i:− variants from clinical isolates. J Antimicrob Chemother. (2020) 75:1756–65. doi: 10.1093/jac/dkaa115 32274508

[50] Bawn M Alikhan N-F Thilliez G Kirkwood M Wheeler NE Petrovska L. Evolution of *Salmonella enterica* serotype Typhimurium driven by anthropogenic selection and niche adaptation. PLoS Genet. (2020) 16:e1008850. doi: 10.1371/journal.pgen.1008850, PMID: 32511244 PMC7302871

[51] Gomes-Neves E Antunes P Manageiro V Gärtner F Caniça M da Costa JMC. Clinically relevant multidrug resistant *Salmonella enterica* in swine and meat handlers at the abattoir. Vet Microbiol. (2014) 168:229–33. doi: 10.1016/j.vetmic.2013.10.017, PMID: 24239169

[52] Possebon FS Tiba Casas MR Nero LA Yamatogi RS Araújo JP Jr De AN PJP. Prevalence, antibiotic resistance, PFGE and MLST characterization of *Salmonella* in swine mesenteric lymph nodes. Prev Vet Med. (2020) 179:105024. doi: 10.1016/j.prevetmed.2020.105024, PMID: 32417637

[53] Arfken AM Frey JF Summers KL. Temporal dynamics of the gut bacteriome and mycobiome in the weanling pig. Microorganisms. (2020) 8:868. doi: 10.3390/microorganisms8060868, PMID: 32526857 PMC7356342

[54] Summers KL Frey JF Ramsay TG Arfken AM. The piglet mycobiome during the weaning transition: a pilot study. J Anim Sci. (2019) 97:2889–900. doi: 10.1093/jas/skz182, PMID: 31136650 PMC6606507

[55] Patterson SK Kim HB Borewicz K Isaacson RE. Towards an understanding of *Salmonella enterica* serovar Typhimurium persistence in swine. Anim Health Res Rev. (2016) 17:159–68. doi: 10.1017/S1466252316000165, PMID: 28155802

[56] Argüello H Estellé J Leonard FC Crispie F Cotter PD O’Sullivan O. Influence of the intestinal microbiota on colonization resistance to *Salmonella* and the shedding pattern of naturally exposed pigs. mSystems. (2019) 4:e00021–19. doi: 10.1128/mSystems.00021-19 31020042 PMC6478965

[57] Rivera-Chávez F Lopez CA Bäumler AJ. Oxygen as a driver of gut dysbiosis. Free Radic Biol Med. (2017) 105:93–101. doi: 10.1016/j.freeradbiomed.2016.09.022, PMID: 27677568

[58] Rivera-Chávez F Zhang LF Faber F Lopez CA Byndloss MX Olsan EE. Depletion of butyrate-producing Clostridia from the gut microbiota drives an aerobic luminal expansion of *Salmonella*. Cell Host Microbe. (2016) 19:443–54. doi: 10.1016/j.chom.2016.03.004, PMID: 27078066 PMC4832419

[59] Litvak Y Mon KKZ Nguyen H Chanthavixay G Liou M Velazquez EM. Commensal Enterobacteriaceae protect against *Salmonella* colonization through oxygen competition. Cell Host Microbe. (2019) 25:128–139.e5. doi: 10.1016/j.chom.2018.12.003, PMID: 30629913 PMC12036633

[60] Bukin Y Galachyants Y Morozov I Bukin SV Zakharenko AS Zemskaya TI. The effect of 16S rRNA region choice on bacterial community metabarcoding results. Sci Data. (2019) 6:190007. doi: 10.1038/sdata.2019.7, PMID: 30720800 PMC6362892

[61] Wang X Howe S Wei X Deng F Tsai T Chai J. Comprehensive cultivation of the swine gut microbiome reveals high bacterial diversity and guides bacterial isolation in pigs. mSystems. (2021) 6:e00477–21. doi: 10.1128/mSystems.00477-21 34282935 PMC8407297

[62] Umu ÖCO Frank JA Fangel JU Oostindjer M da Silva CS Bolhuis EJ. Resistant starch diet induces change in the swine microbiome and a predominance of beneficial bacterial populations. Microbiome. (2015) 3:16. doi: 10.1186/s40168-015-0078-5, PMID: 25905018 PMC4405844

[63] Tiwari UP Mandal RK Neupane KR Mishra B Jha R. Starchy and fibrous feedstuffs differ in their in vitro digestibility and fermentation characteristics and differently modulate gut microbiota of swine. J Anim Sci Biotechnol. (2022) 13:53. doi: 10.1186/s40104-022-00699-y, PMID: 35501888 PMC9063073

[64] Helm ET Gabler NK Burrough ER. Highly fermentable fiber alters fecal microbiota and mitigates swine dysentery induced by *Brachyspira hyodysenteriae*. Animals. (2021) 11:396. doi: 10.3390/ani11020396, PMID: 33557345 PMC7915590

[65] Liu P Zhao J Wang W Guo P Lu W Wang C. Dietary corn bran altered the diversity of microbial communities and cytokine production in weaned pigs. Front Microbiol. (2018) 9:2090. doi: 10.3389/fmicb.2018.02090 30233555 PMC6131307

[66] Oh JK Vasquez R Kim SH Hwang I-C Song JH Park JH. Multispecies probiotics alter fecal short-chain fatty acids and lactate levels in weaned pigs by modulating gut microbiota. J Anim Sci Technol. (2021) 63:1142–58. doi: 10.5187/jast.2021.e94, PMID: 34796353 PMC8564300

[67] Prabhu R Altman E Eiteman MA. Lactate and acrylate metabolism by *Megasphaera elsdenii* under batch and steady-state conditions. Appl Environ Microbiol. (2012) 78:8564–70. doi: 10.1128/AEM.02443-12, PMID: 23023753 PMC3502912

[68] Gillis CC Winter MG Chanin RB Zhu W Spiga L Winter SE. Host-derived metabolites modulate transcription of *Salmonella* genes involved in l -lactate utilization during gut colonization. Infect Immun. (2019) 87:e00773–18. doi: 10.1128/IAI.00773-18, PMID: 30617205 PMC6434127

[69] Gillis CC Hughes ER Spiga L Winter MG Zhu W Furtado de Carvalho T. Dysbiosis-associated change in host metabolism generates lactate to support *Salmonella* growth. Cell Host Microbe. (2018) 23:54–64.e6. doi: 10.1016/j.chom.2017.11.006, PMID: 29276172 PMC5764812

[70] Lauridsen C. Effects of dietary fatty acids on gut health and function of pigs pre- and post-weaning. J Anim Sci. (2020) 98:skaa086. doi: 10.1093/jas/skaa086, PMID: 32215565 PMC7323257

[71] Tett A Huang KD Asnicar F Fehlner-Peach H Pasolli E Karcher N. The *Prevotella copri* complex comprises four distinct clades underrepresented in westernized populations. Cell Host Microbe. (2019) 26:666–679.e7. doi: 10.1016/j.chom.2019.08.018, PMID: 31607556 PMC6854460

[72] Fehlner-Peach H Magnabosco C Raghavan V Scher JU Tett A Cox LM. Distinct polysaccharide utilization profiles of human intestinal *Prevotella copri* isolates. Cell Host Microbe. (2019) 26:680–690.e5. doi: 10.1016/j.chom.2019.10.013, PMID: 31726030 PMC7039456

[73] Nii T Maeda Y Motooka D Naito M Matsumoto Y Ogawa T. Genomic repertoires linked with pathogenic potency of arthritogenic *Prevotella copri* isolated from the gut of patients with rheumatoid arthritis. Ann Rheum Dis. (2023) 82:621–9. doi: 10.1136/ard-2022-222881, PMID: 36627170 PMC10176341

[74] Arfken AM Frey JF Ramsay TG Summers KL. Yeasts of burden: exploring the mycobiome–bacteriome of the piglet GI tract. Front Microbiol. (2019) 10:2286. doi: 10.3389/fmicb.2019.02286, PMID: 31649634 PMC6792466

[75] Summers KL Foster Frey J Arfken AM. Characterization of Kazachstania slooffiae, a proposed commensal in the porcine gut. J Fungi. (2021) 7:146. doi: 10.3390/jof7020146, PMID: 33671322 PMC7922399

[76] Zhou Z Alikhan N-F Mohamed K Fan Y the Agama Study Group Achtman M. The EnteroBase user’s guide, with case studies on *Salmonella* transmissions, *Yersinia pestis* phylogeny, and Escherichia core genomic diversity. Genome Res. (2020) 30:138–52. doi: 10.1101/gr.251678.119, PMID: 31809257 PMC6961584

