# Supplementary material for: Salmonella enterica induces biogeography-specific changes in the gut microbiome of pigs
Source: Front Vet Sci. 2023 Sep 14;10:1186554. doi: 10.3389/fvets.2023.1186554 (PMC10537282; doi:10.3389/fvets.2023.1186554)
Supplement: Supplementary file 1 [file Data_Sheet_1.PDF]

## SUPPLEMENTARY MATERIAL

A

| Ileal contents | DPI 2 | DPI 4 | DPI 28 |
|---|---|---|---|
| Control | 3 | 3 | 6 |
| Derby | 5 | 4 | 9 |
| Monophasic | 5 | 5 | 10 |
| Typhimurium | 5 | 5 | 10 |

B

| Apex contents | DPI 2 | DPI 4 | DPI 28 |
|---|---|---|---|
| Control | 3 | 3 | 6 |
| Derby | 5 | 5 | 9 |
| Monophasic | 4 | 5 | 9 |
| Typhimurium | 5 | 5 | 10 |

C

| Fecal contents | DPI 0 | DPI 2 | DPI 4 | DPI 7 | DPI 14 | DPI 21 | DPI 28 |
|---|---|---|---|---|---|---|---|
| Control | 12 | 12 | 9 | 6 | 6 | 6 | 6 |
| Derby | 19 | 19 | 14 | 9 | 9 | 9 | 9 |
| Monophasic | 20 | 20 | 15 | 10 | 10 | 10 | 9 |
| Typhimurium | 20 | 20 | 15 | 10 | 10 | 9 | 10 |

**Supplementary Figure 1** | Distribution of animals (samples) collected across DPI for ileum (A), colonic apex (apex) (B), and fecal (C) contents across treatments (Control, Derby, Monophasic, and Typhimurium). Only samples that passed the bioinformatics quality control (as described in the methods section) were reported here and included in the biostatistical analysis.

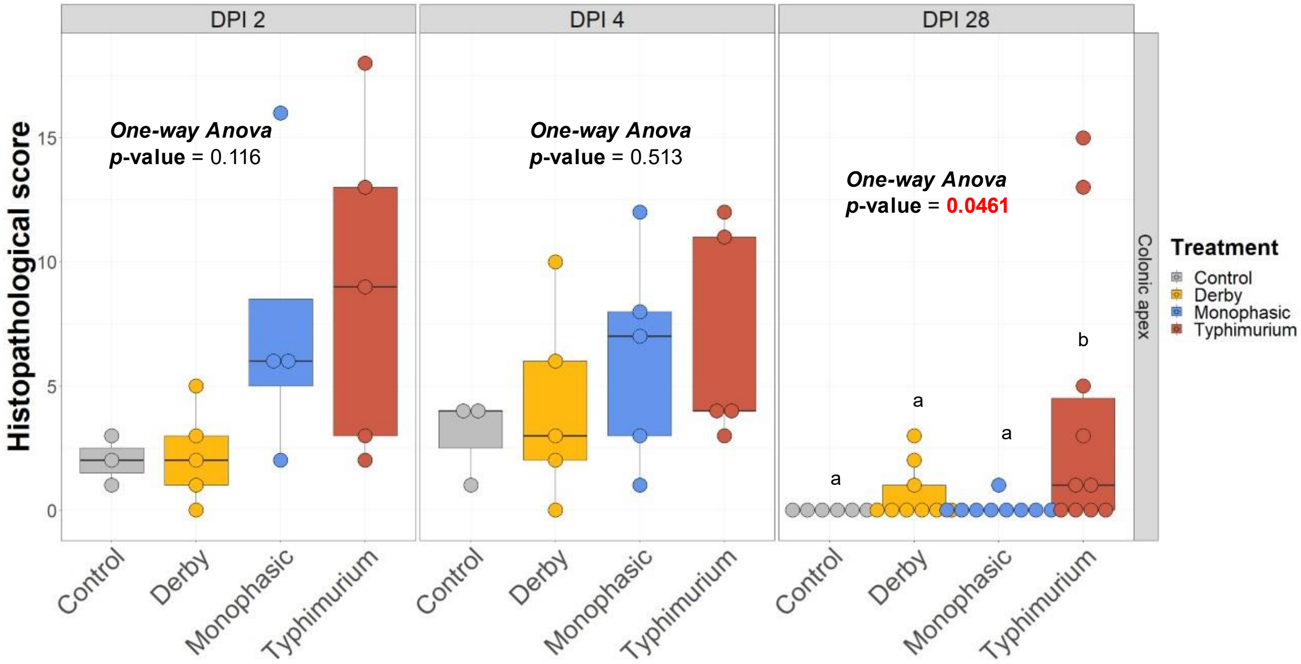


**Supplementary Figure 2 |** Colonic apex histopathological scores across treatments (Control, Derby, Monophasic, and Typhimurium) and DPI 2, 4, and 28. Statistical analysis was done using an ANOVA followed by a pairwise T-test ($p < 0.05$). Different superscript letters indicate significant differences between treatments. Only animals that had microbiome samples passed through the bioinformatic cut-off for quality control were included in this analysis.

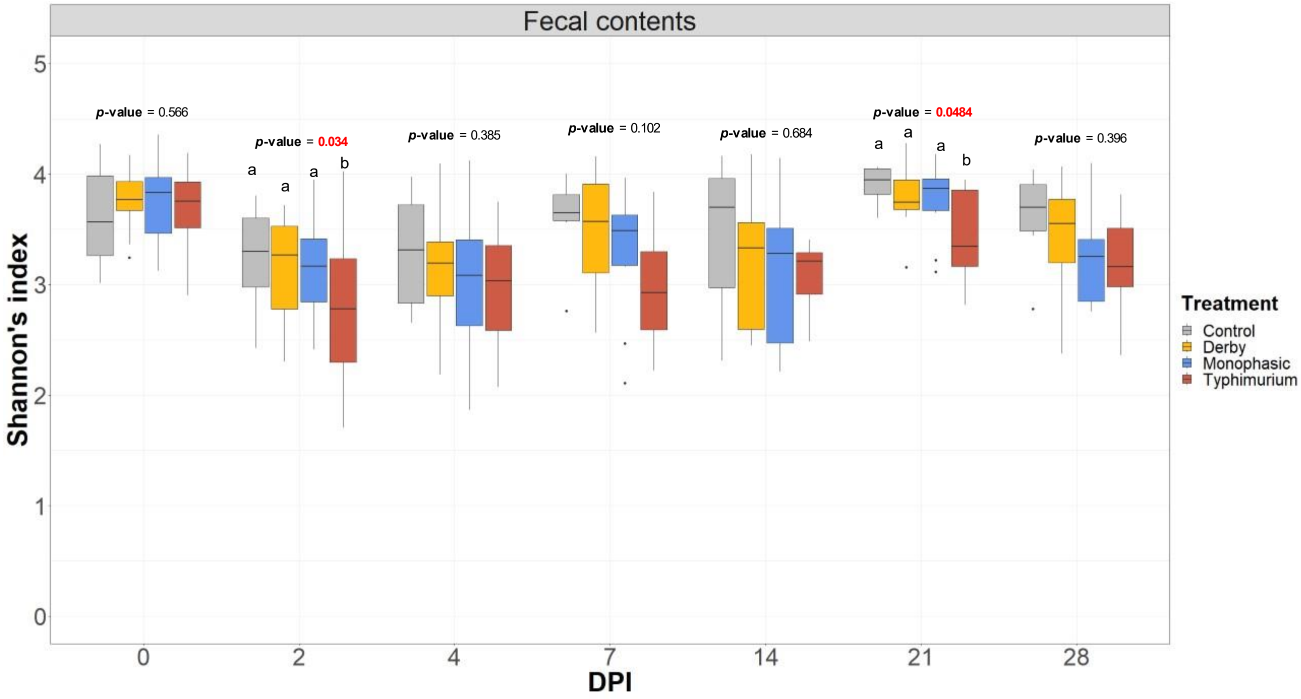


**Supplementary Figure 3 |** Alpha-diversity analysis (Shannon's index) across treatments (Control, Derby, Monophasic, and Typhimurium) and DPI 2, 4, and 28, for fecal samples only. Statistical analysis was done using an ANOVA followed by a pairwise T-test ($p < 0.05$). Different superscript letters indicate significant differences between treatments. Only animals that had microbiome samples passed through the bioinformatic cut-off for quality control were included in this analysis.

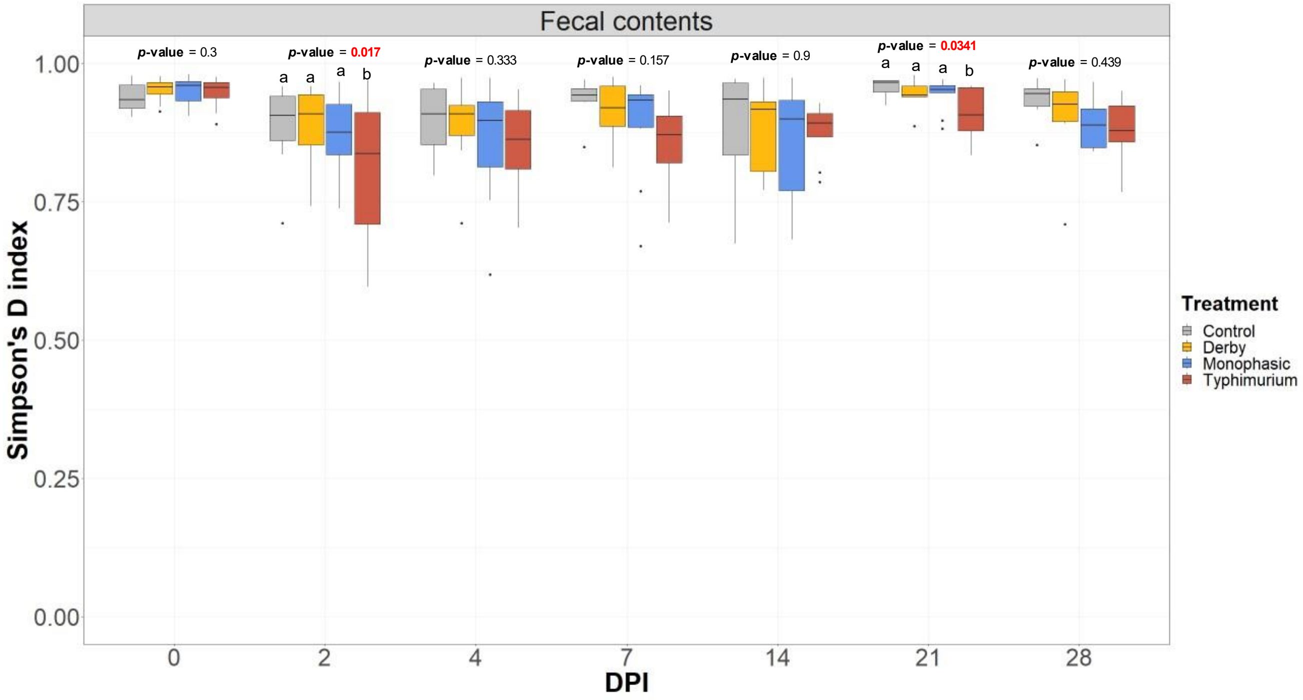


**Supplementary Figure 4 |** Alpha-diversity analysis (Simpson's D index) across treatments (Control, Derby, Monophasic, and Typhimurium) and DPI 2, 4, and 28, for fecal samples only. Statistical analysis was done using an ANOVA followed by a pairwise T-test ($p < 0.05$). Different superscript letters indicate significant differences between treatments. Only animals that had microbiome samples passed through the bioinformatic cut-off for quality control were included in this analysis.

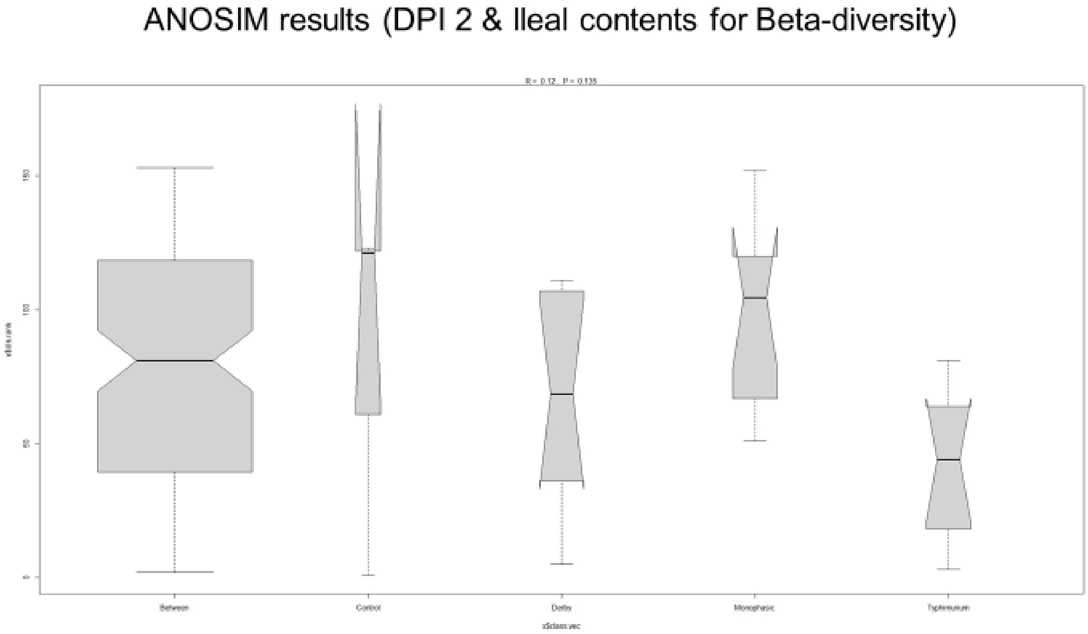


**Supplementary Figure 5 |** Ileal contents (day post-infection – DPI 2) analysis of similarity (ANOSIM) results modeled based on the Bray-Curtis distance (beta-diversity) between samples (response variable) and the experimental treatments (groups) as explanatory variable. A dissimilarity matrix was generated with the vegdist() in R from the Vegan package. In brief, when groups are significantly different ($p < 0.05$ – P refers to *p*-value on the figure) in their microbial community composition, then compositional dissimilarities between the groups is greater than those within the groups (as described here https://www.rdocumentation.org/packages/vegan/versions/2.3-5/topics/anosim). On the y-axis x$dis.rank is the rank of dissimilarity entry. On the x-axis are all individual treatments (groups) and the between groups entry. The higher the ANOSIM statistic R (value closer to 1), the more dissimilar the communities between treatments; and vice-versa (see examples here https://jkzorz.github.io/2019/06/11/ANOSIM-test.html; https://sites.google.com/site/mb3gustame/hypothesis-tests/anosim).

## ANOSIM results (DPI 4 & Ileal contents for Beta-diversity)

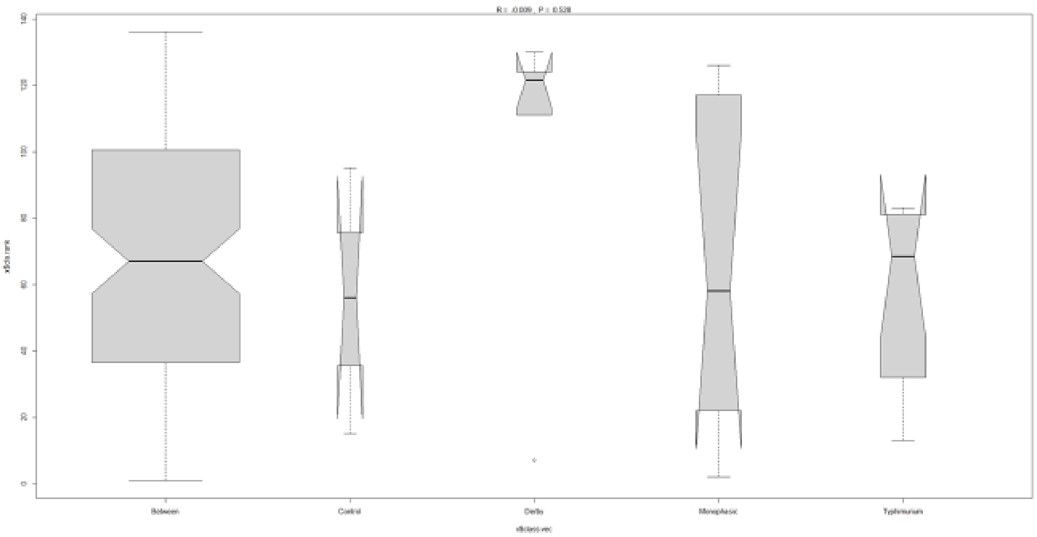

**Supplementary Figure 6 |** Ileal contents (day post-infection - DPI 4) analysis of similarity (ANOSIM) results modeled based on the Bray-Curtis distance (beta-diversity) between samples (response variable) and the experimental treatments (groups) as explanatory variable. A dissimilarity matrix was generated with the vegdist() in R from the Vegan package. In brief, when groups are significantly different ($p < 0.05$ – P refers to *p*-value on the figure) in their microbial community composition, then compositional dissimilarities between the groups is greater than those within the groups (as described here https://www.rdocumentation.org/packages/vegan/versions/2.3-5/topics/anosim). On the y-axis x$dis.rank is the rank of dissimilarity entry. On the x-axis are all individual treatments (groups) and the between groups entry. The higher the ANOSIM statistic R (value closer to 1), the more dissimilar the communities between treatments; and vice-versa (see examples here https://jkzorz.github.io/2019/06/11/ANOSIM-test.html; https://sites.google.com/site/mb3gustame/hypothesis-tests/anosim).

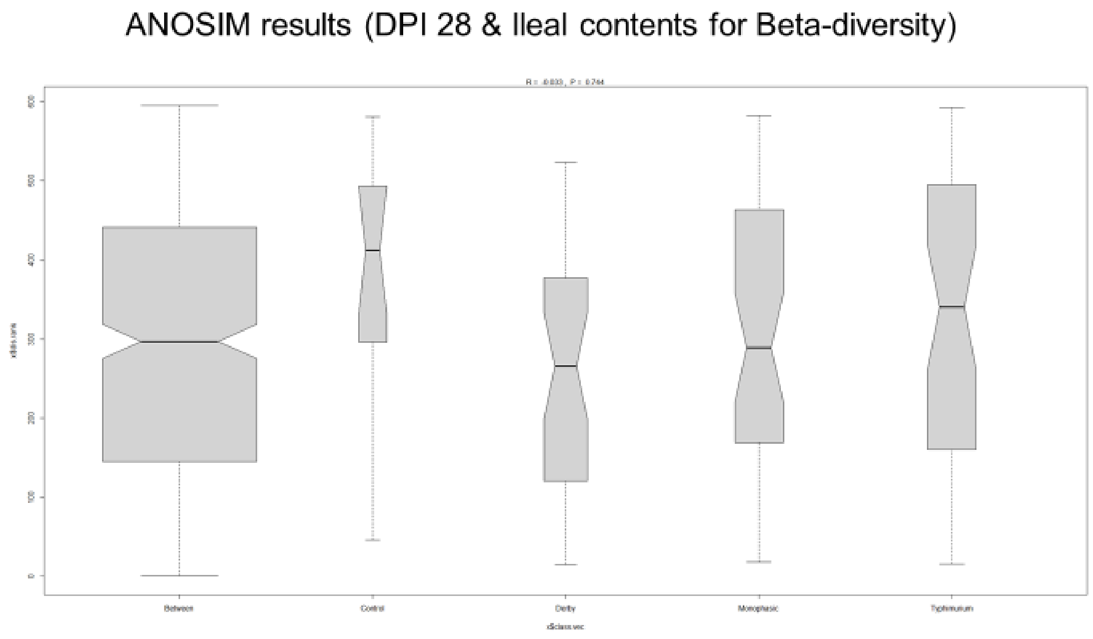


**Supplementary Figure 7 |** Ileal contents (day post-infection - DPI 28) analysis of similarity (ANOSIM) results modeled based on the Bray-Curtis distance (beta-diversity) between samples (response variable) and the experimental treatments (groups) as explanatory variable. A dissimilarity matrix was generated with the vegdist() in R from the Vegan package. In brief, when groups are significantly different ($p < 0.05$ – P refers to *p*-value on the figure) in their microbial community composition, then compositional dissimilarities between the groups is greater than those within the groups (as described here https://www.rdocumentation.org/packages/vegan/versions/2.3-5/topics/anosim). On the y-axis x$dis.rank is the rank of dissimilarity entry. On the x-axis are all individual treatments (groups) and the between groups entry. The higher the ANOSIM statistic R (value closer to 1), the more dissimilar the communities between treatments; and vice-versa (see examples here https://jkzorz.github.io/2019/06/11/ANOSIM-test.html; https://sites.google.com/site/mb3gustame/hypothesis-tests/anosim).

ANOSIM results (DPI 2 & Apex contents for Beta-diversity)

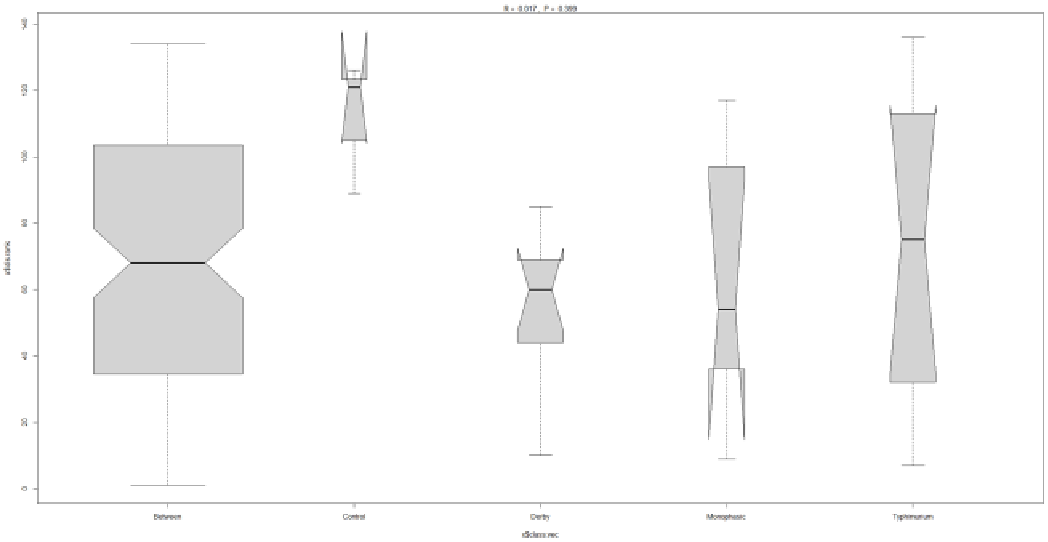


**Supplementary Figure 8 |** Colonic apex contents (day post-infection - DPI 2) analysis of similarity (ANOSIM) results modeled based on the Bray-Curtis distance (beta-diversity) between samples (response variable) and the experimental treatments (groups) as explanatory variable. A dissimilarity matrix was generated with the vegdist() in R from the Vegan package. In brief, when groups are significantly different ($p < 0.05$ – P refers to *p*-value on the figure) in their microbial community composition, then compositional dissimilarities between the groups is greater than those within the groups (as described here https://www.rdocumentation.org/packages/vegan/versions/2.3-5/topics/anosim). On the y-axis x$dis.rank is the rank of dissimilarity entry. On the x-axis are all individual treatments (groups) and the between groups entry. The higher the ANOSIM statistic R (value closer to 1), the more dissimilar the communities between treatments; and vice-versa (see examples here https://jkzorz.github.io/2019/06/11/ANOSIM-test.html; https://sites.google.com/site/mb3gustame/hypothesis-tests/anosim).

ANOSIM results (DPI 4 & Apex contents for Beta-diversity)

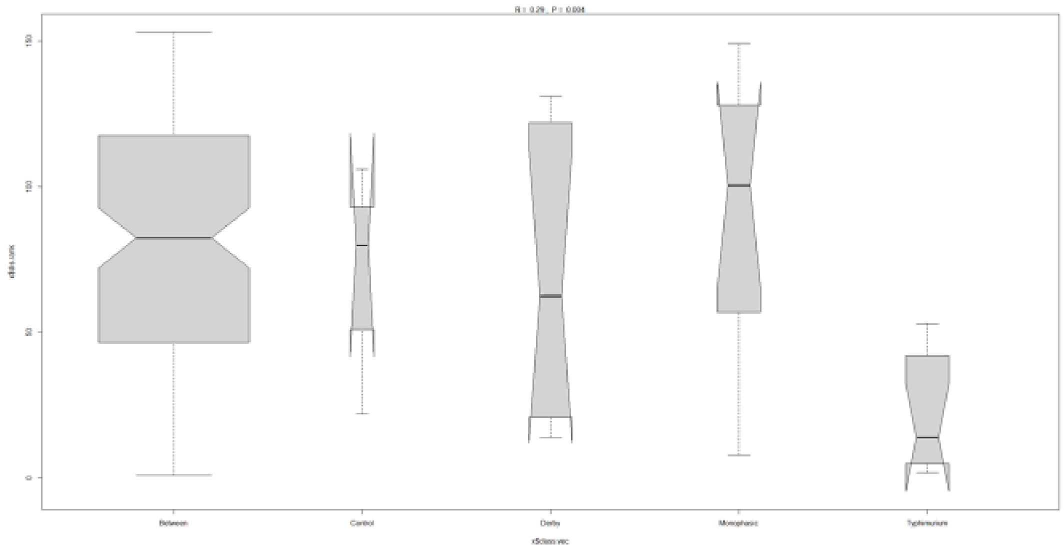


**Supplementary Figure 9 |** Colonic apex contents (day post-infection - DPI 4) analysis of similarity (ANOSIM) results modeled based on the Bray-Curtis distance (beta-diversity) between samples (response variable) and the experimental treatments (groups) as explanatory variable. A dissimilarity matrix was generated with the vegdist() in R from the Vegan package. In brief, when groups are significantly different ($p < 0.05$ – P refers to *p*-value on the figure) in their microbial community composition, then compositional dissimilarities between the groups is greater than those within the groups (as described here https://www.rdocumentation.org/packages/vegan/versions/2.3-5/topics/anosim). On the y-axis x$dis.rank is the rank of dissimilarity entry. On the x-axis are all individual treatments (groups) and the between groups entry. The higher the ANOSIM statistic R (value closer to 1), the more dissimilar the communities between treatments; and vice-versa (see examples here https://jkzorz.github.io/2019/06/11/ANOSIM-test.html; https://sites.google.com/site/mb3gustame/hypothesis-tests/anosim).

ANOSIM results (DPI 28 & Apex contents for Beta-diversity)

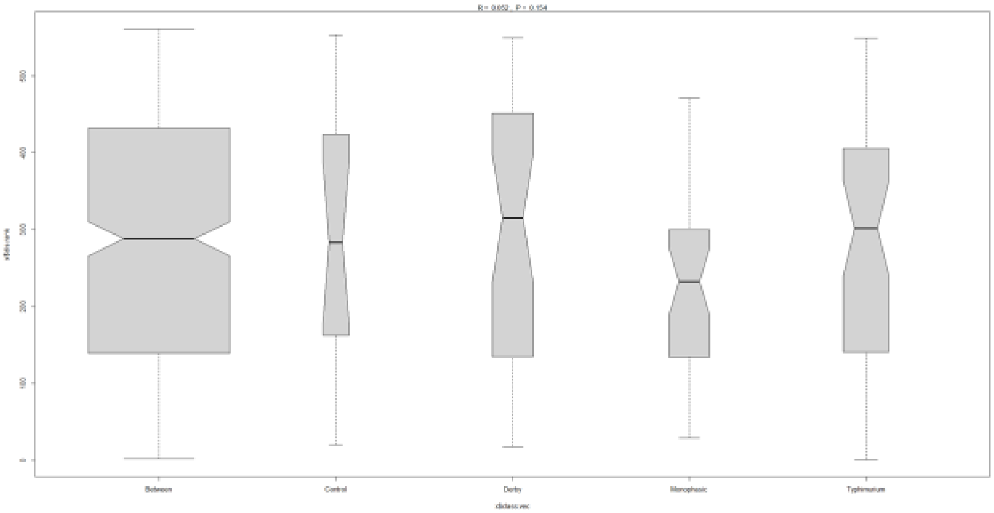


**Supplementary Figure 10 |** Colonic apex contents (day post-infection - DPI 29) analysis of similarity (ANOSIM) results modeled based on the Bray-Curtis distance (beta-diversity) between samples (response variable) and the experimental treatments (groups) as explanatory variable. A dissimilarity matrix was generated with the vegdist() in R from the Vegan package. In brief, when groups are significantly different ($p < 0.05$ – P refers to *p*-value on the figure) in their microbial community composition, then compositional dissimilarities between the groups is greater than those within the groups (as described here https://www.rdocumentation.org/packages/vegan/versions/2.3-5/topics/anosim). On the y-axis x$dis.rank is the rank of dissimilarity entry. On the x-axis are all individual treatments (groups) and the between groups entry. The higher the ANOSIM statistic R (value closer to 1), the more dissimilar the communities between treatments; and vice-versa (see examples here https://jkzorz.github.io/2019/06/11/ANOSIM-test.html; https://sites.google.com/site/mb3gustame/hypothesis-tests/anosim).

ANOSIM results (DPI 2 & Fecal contents for Beta-diversity)

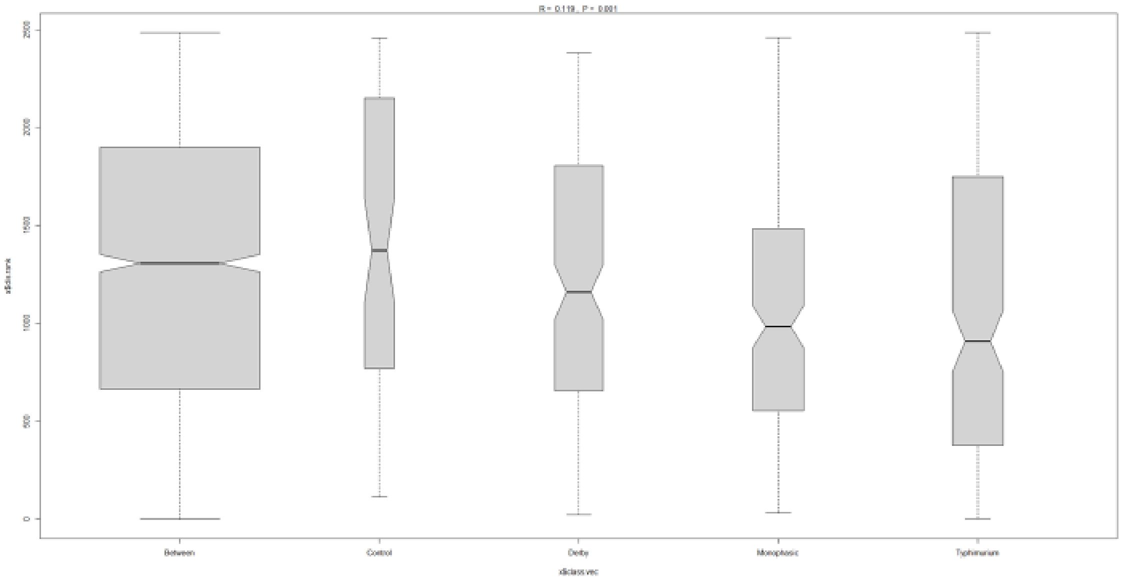


**Supplementary Figure 11 |** Fecal contents (day post-infection - DPI 2) analysis of similarity (ANOSIM) results modeled based on the Bray-Curtis distance (beta-diversity) between samples (response variable) and the experimental treatments (groups) as explanatory variable. A dissimilarity matrix was generated with the vegdist() in R from the Vegan package. In brief, when groups are significantly different ($p < 0.05$ – P refers to *p*-value on the figure) in their microbial community composition, then compositional dissimilarities between the groups is greater than those within the groups (as described here https://www.rdocumentation.org/packages/vegan/versions/2.3-5/topics/anosim). On the y-axis x$dis.rank is the rank of dissimilarity entry. On the x-axis are all individual treatments (groups) and the between groups entry. The higher the ANOSIM statistic R (value closer to 1), the more dissimilar the communities between treatments; and vice-versa (see examples here https://jkzorz.github.io/2019/06/11/ANOSIM-test.html; https://sites.google.com/site/mb3gustame/hypothesis-tests/anosim).

ANOSIM results (DPI 4 & Fecal contents for Beta-diversity)

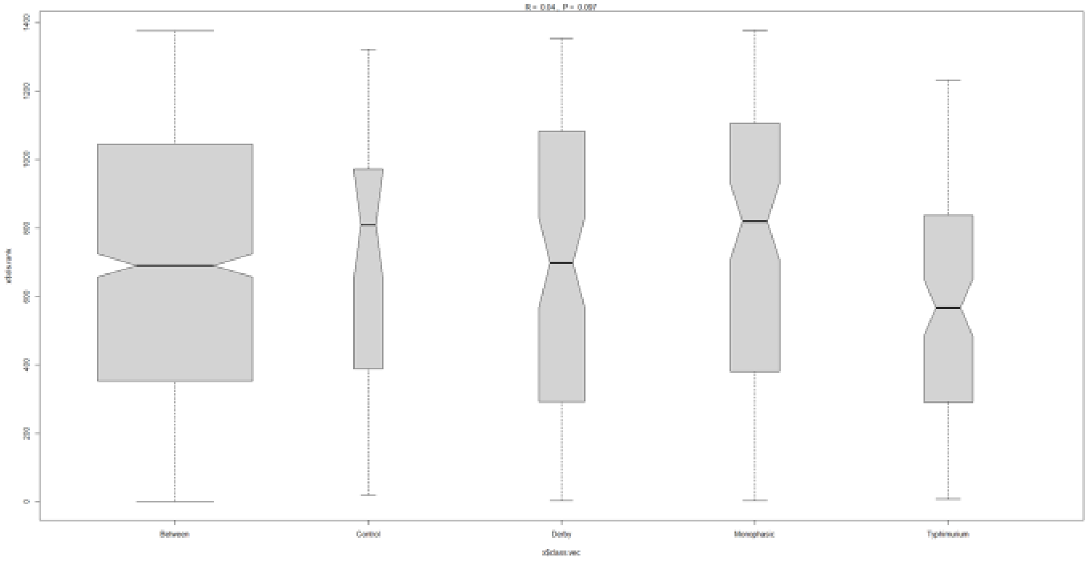


**Supplementary Figure 12 |** Fecal contents (day post-infection - DPI 4) analysis of similarity (ANOSIM) results modeled based on the Bray-Curtis distance (beta-diversity) between samples (response variable) and the experimental treatments (groups) as explanatory variable. A dissimilarity matrix was generated with the vegdist() in R from the Vegan package. In brief, when groups are significantly different ($p < 0.05$ – P refers to *p*-value on the figure) in their microbial community composition, then compositional dissimilarities between the groups is greater than those within the groups (as described here https://www.rdocumentation.org/packages/vegan/versions/2.3-5/topics/anosim). On the y-axis x$dis.rank is the rank of dissimilarity entry. On the x-axis are all individual treatments (groups) and the between groups entry. The higher the ANOSIM statistic R (value closer to 1), the more dissimilar the communities between treatments; and vice-versa (see examples here https://jkzorz.github.io/2019/06/11/ANOSIM-test.html; https://sites.google.com/site/mb3gustame/hypothesis-tests/anosim).

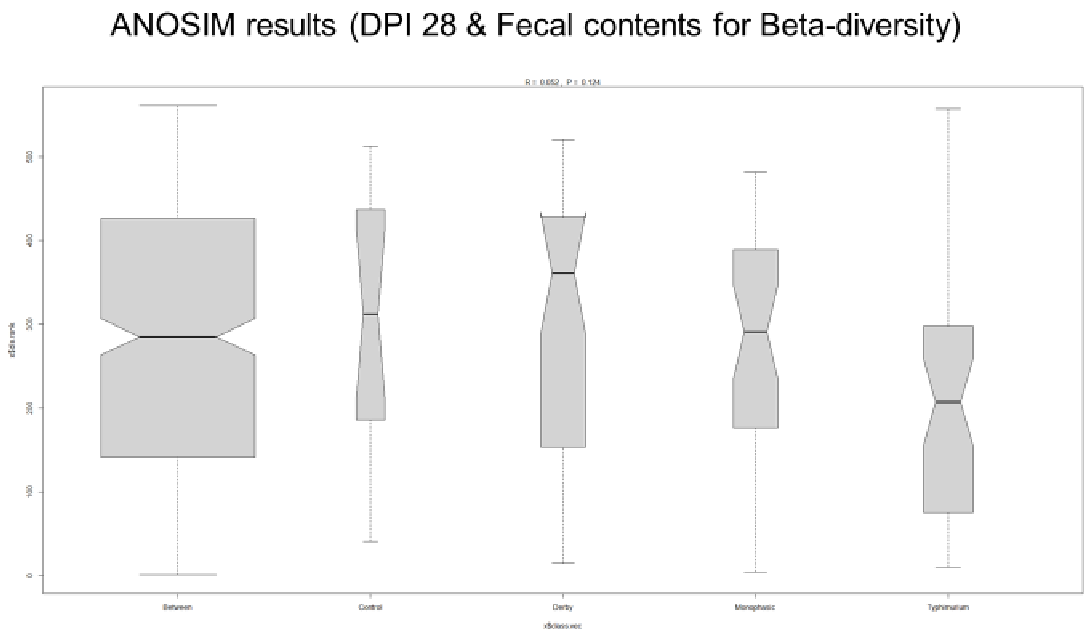


**Supplementary Figure 13 |** Fecal contents (day post-infection - DPI 28) analysis of similarity (ANOSIM) results modeled based on the Bray-Curtis distance (beta-diversity) between samples (response variable) and the experimental treatments (groups) as explanatory variable. A dissimilarity matrix was generated with the vegdist() in R from the Vegan package. In brief, when groups are significantly different ($p < 0.05$ – P refers to $p$-value on the figure) in their microbial community composition, then compositional dissimilarities between the groups is greater than those within the groups (as described here https://www.rdocumentation.org/packages/vegan/versions/2.3-5/topics/anosim). On the y-axis x$dis.rank is the rank of dissimilarity entry. On the x-axis are all individual treatments (groups) and the between groups entry. The higher the ANOSIM statistic R (value closer to 1), the more dissimilar the communities between treatments; and vice-versa (see examples here https://jkzorz.github.io/2019/06/11/ANOSIM-test.html; https://sites.google.com/site/mb3gustame/hypothesis-tests/anosim).

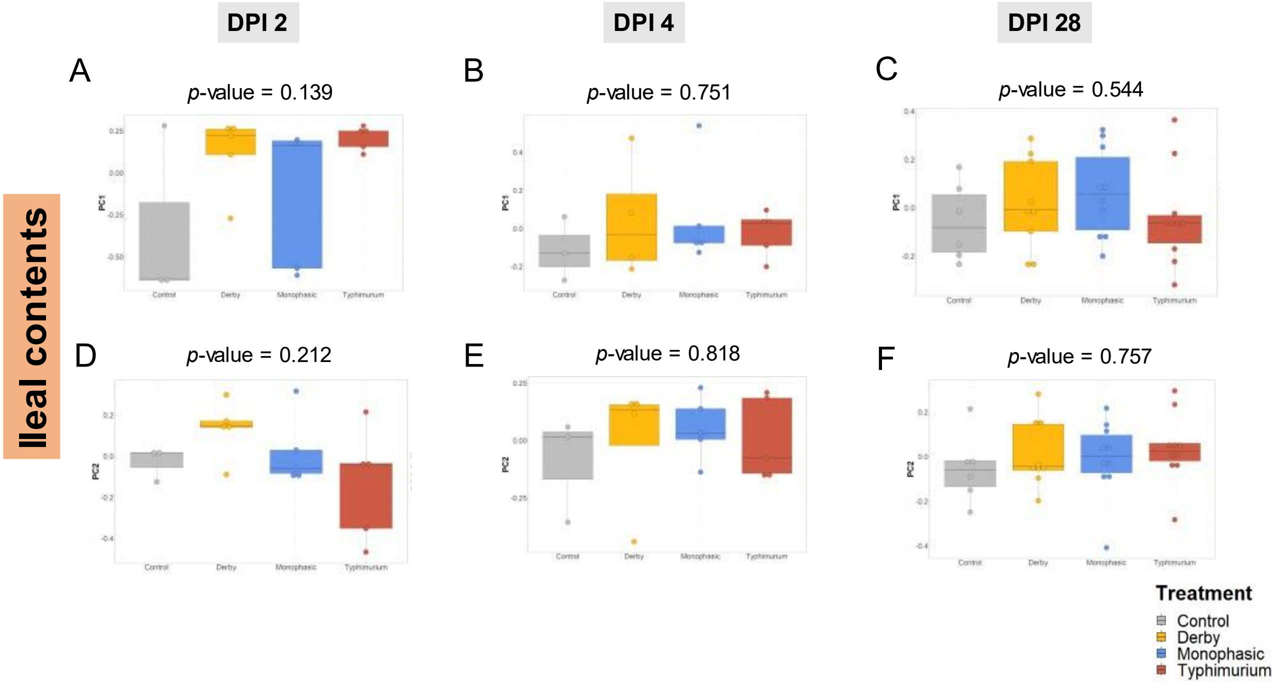


**Supplementary Figure 14 |** Beta-diversity decomposition analysis for ileal contents across DPI (2, 4, and 28) and treatments (Control, Derby, Monophasic, and Typhimurium). PC1 (A-C) and PC2 (D-F) were analyzed separately to examine dispersion and volatility of microbial communities across treatments. Statistical analysis was done using an ANOVA followed by a pairwise T-test ($p < 0.05$). Different superscript letters indicate significant differences between treatments. Only animals that had microbiome samples passed through the bioinformatic cut-off for quality control were included in this analysis.

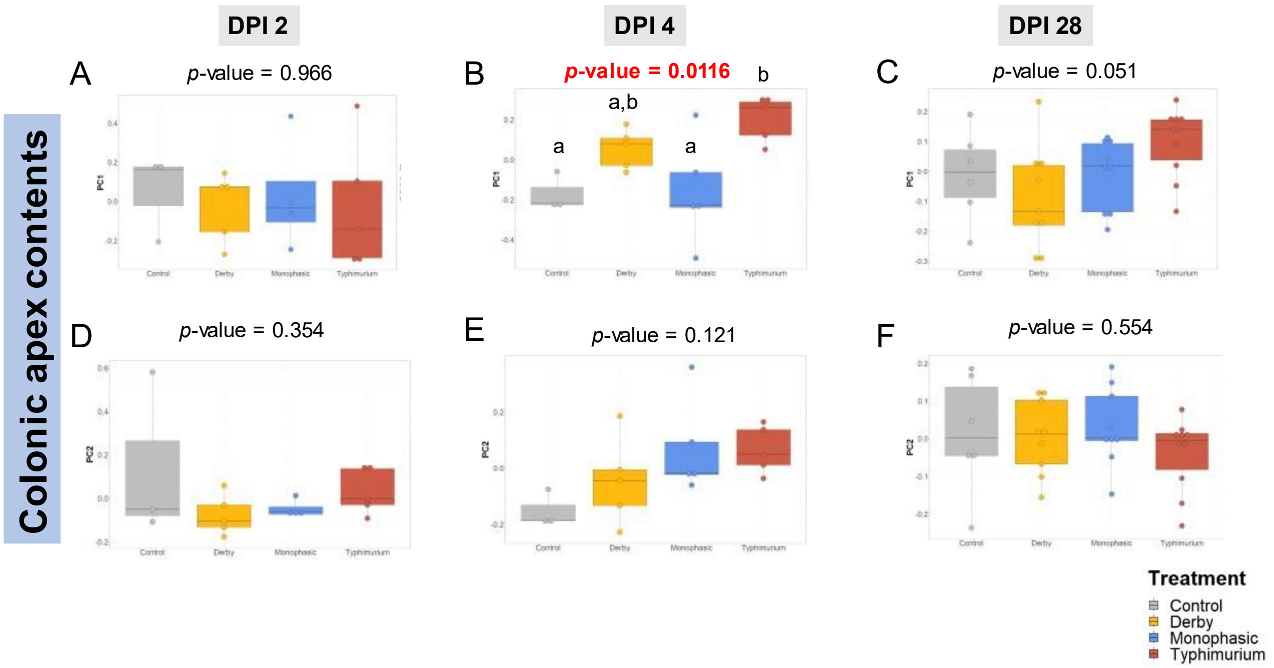


**Supplementary Figure 15 |** Beta-diversity decomposition analysis for colonic apex contents across DPI (2, 4, and 28) and treatments (Control, Derby, Monophasic, and Typhimurium). PC1 (A-C) and PC2 (D-F) were analyzed separately to examine dispersion and volatility of microbial communities across treatments. Statistical analysis was done using an ANOVA followed by a pairwise T-test ($p < 0.05$). Different superscript letters indicate significant differences between treatments. Only animals that had microbiome samples passed through the bioinformatic cut-off for quality control were included in this analysis.

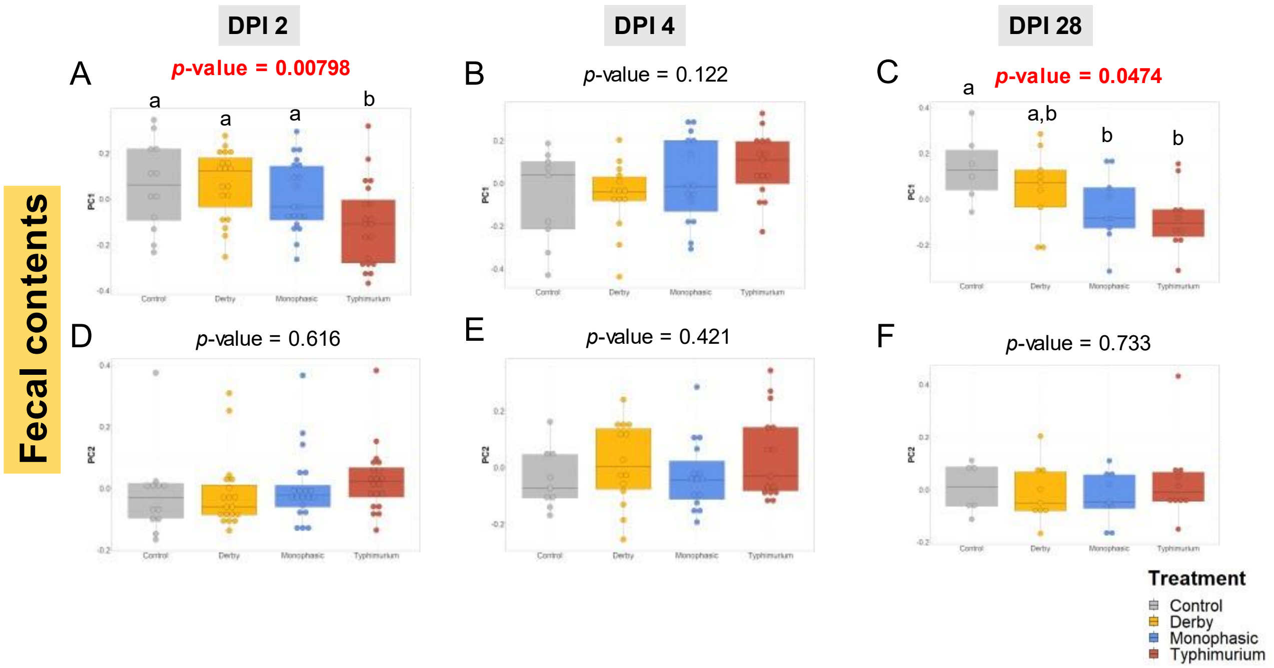


**Supplementary Figure 16** | Beta-diversity decomposition analysis for fecal contents across DPI (2, 4, and 28) and treatments (Control, Derby, Monophasic, and Typhimurium). PC1 (A-C) and PC2 (D-F) were analyzed separately to examine dispersion and volatility of microbial communities across treatments. Statistical analysis was done using an ANOVA followed by a pairwise T-test ($p < 0.05$). Different superscript letters indicate significant differences between treatments. Only animals that had microbiome samples passed through the bioinformatic cut-off for quality control were included in this analysis.

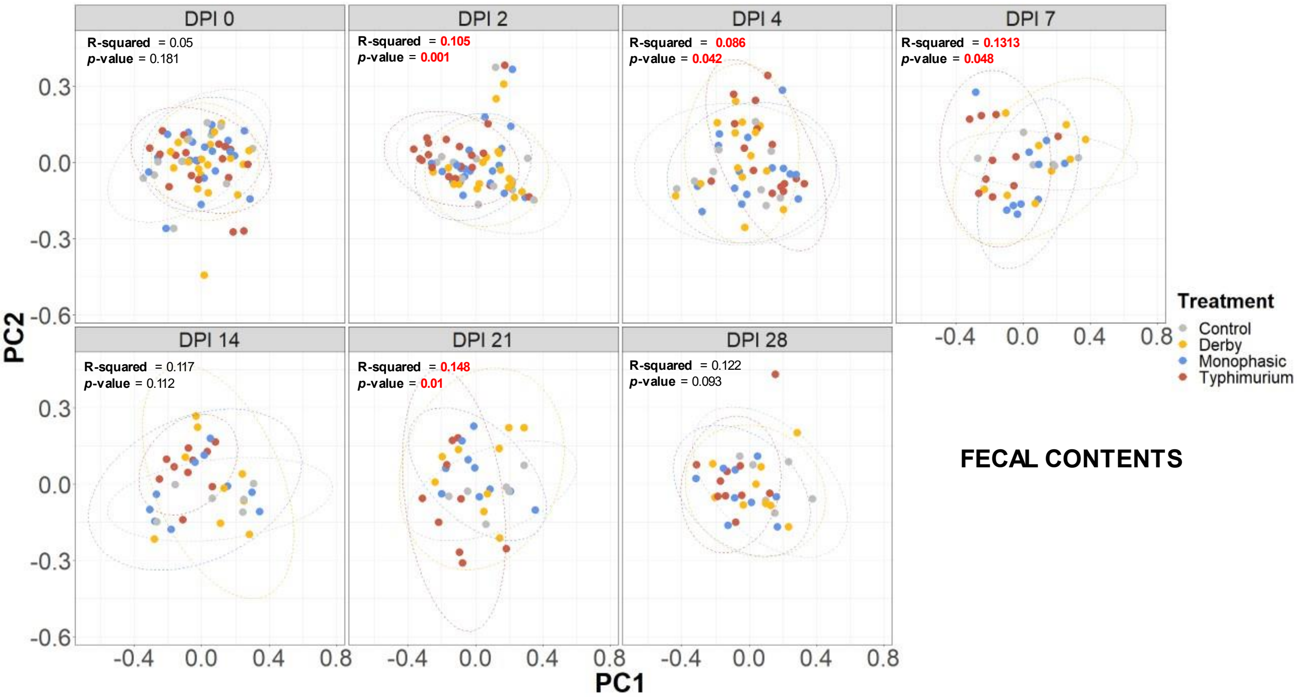


**Supplementary Figure 17 |** Beta-diversity analysis of fecal microbiome across treatments and DPI. A Bray-Curtis distance matrix was used to calculate the beta-diversity between treatments. Two principal coordinates are shown across all DPI. A PERMANOVA model was used to assess the treatment effect on beta-diversity. *P*-values and R-squared statistics are shown in each plot. Significant differences were considered based $p < 0.05$ (results marked in red).

ANOSIM results (DPI 0 & Fecal contents for Beta-diversity)

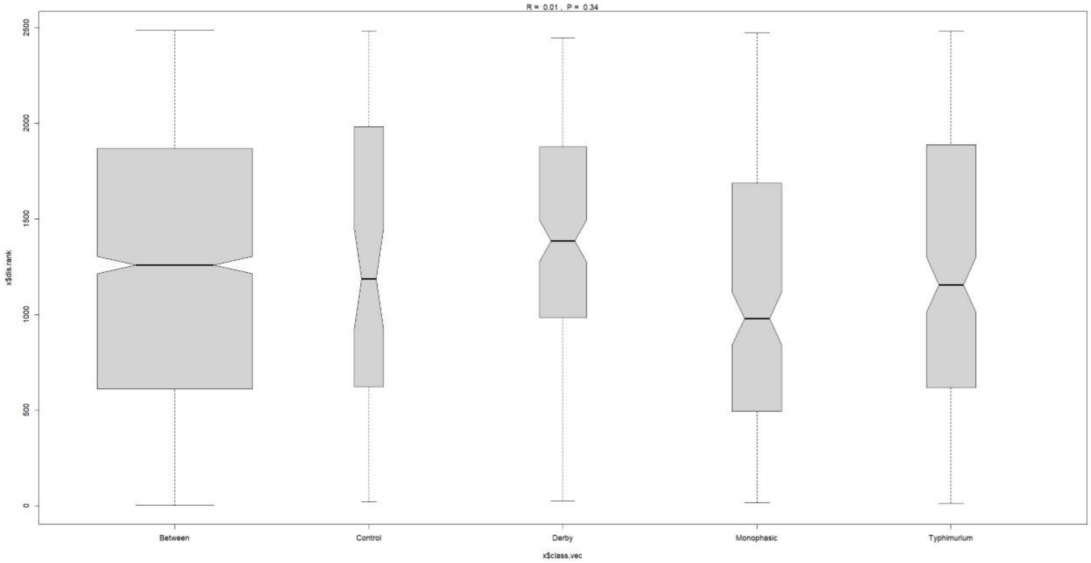


**Supplementary Figure 18 |** Fecal contents (day post-infection - DPI 0) analysis of similarity (ANOSIM) results modeled based on the Bray-Curtis distance (beta-diversity) between samples (response variable) and the experimental treatments (groups) as explanatory variable. A dissimilarity matrix was generated with the vegdist() in R from the Vegan package. In brief, when groups are significantly different ($p < 0.05$ – P refers to *p*-value on the figure) in their microbial community composition, then compositional dissimilarities between the groups is greater than those within the groups (as described here https://www.rdocumentation.org/packages/vegan/versions/2.3-5/topics/anosim). On the y-axis x$dis.rank is the rank of dissimilarity entry. On the x-axis are all individual treatments (groups) and the between groups entry. The higher the ANOSIM statistic R (value closer to 1), the more dissimilar the communities between treatments; and vice-versa (see examples here https://jkzorz.github.io/2019/06/11/ANOSIM-test.html; https://sites.google.com/site/mb3gustame/hypothesis-tests/anosim).

ANOSIM results (DPI 7 & Fecal contents for Beta-diversity)

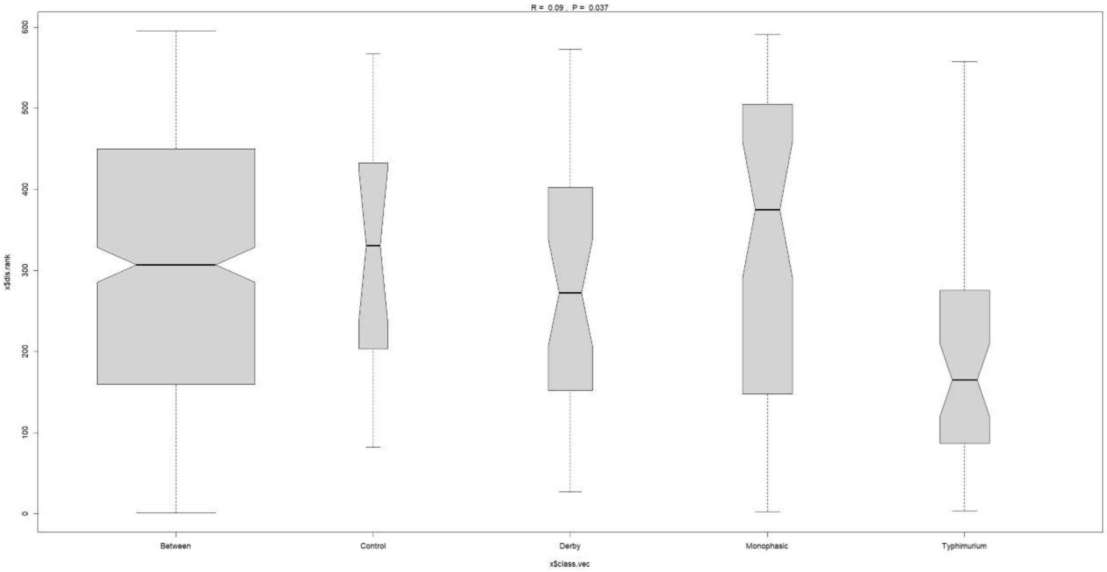


**Supplementary Figure 19 |** Fecal contents (day post-infection - DPI 7) analysis of similarity (ANOSIM) results modeled based on the Bray-Curtis distance (beta-diversity) between samples (response variable) and the experimental treatments (groups) as explanatory variable. A dissimilarity matrix was generated with the vegdist() in R from the Vegan package. In brief, when groups are significantly different ($p < 0.05$ – P refers to *p*-value on the figure) in their microbial community composition, then compositional dissimilarities between the groups is greater than those within the groups (as described here https://www.rdocumentation.org/packages/vegan/versions/2.3-5/topics/anosim). On the y-axis x$dis.rank is the rank of dissimilarity entry. On the x-axis are all individual treatments (groups) and the between groups entry. The higher the ANOSIM statistic R (value closer to 1), the more dissimilar the communities between treatments; and vice-versa (see examples here https://jkzorz.github.io/2019/06/11/ANOSIM-test.html; https://sites.google.com/site/mb3gustame/hypothesis-tests/anosim).

ANOSIM results (DPI 14 & Fecal contents for Beta-diversity)

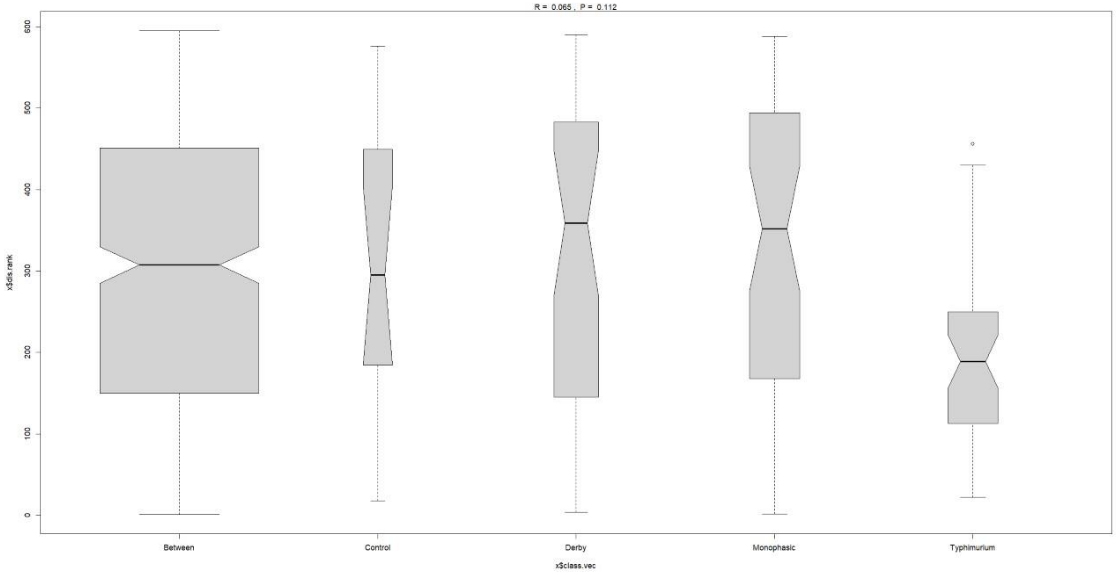


**Supplementary Figure 20 |** Fecal contents (day post-infection - DPI 14) analysis of similarity (ANOSIM) results modeled based on the Bray-Curtis distance (beta-diversity) between samples (response variable) and the experimental treatments (groups) as explanatory variable. A dissimilarity matrix was generated with the vegdist() in R from the Vegan package. In brief, when groups are significantly different ($p < 0.05$ – P refers to *p*-value on the figure) in their microbial community composition, then compositional dissimilarities between the groups is greater than those within the groups (as described here https://www.rdocumentation.org/packages/vegan/versions/2.3-5/topics/anosim). On the y-axis x$dis.rank is the rank of dissimilarity entry. On the x-axis are all individual treatments (groups) and the between groups entry. The higher the ANOSIM statistic R (value closer to 1), the more dissimilar the communities between treatments; and vice-versa (see examples here https://jkzorz.github.io/2019/06/11/ANOSIM-test.html; https://sites.google.com/site/mb3gustame/hypothesis-tests/anosim).

ANOSIM results (DPI 21 & Fecal contents for Beta-diversity)

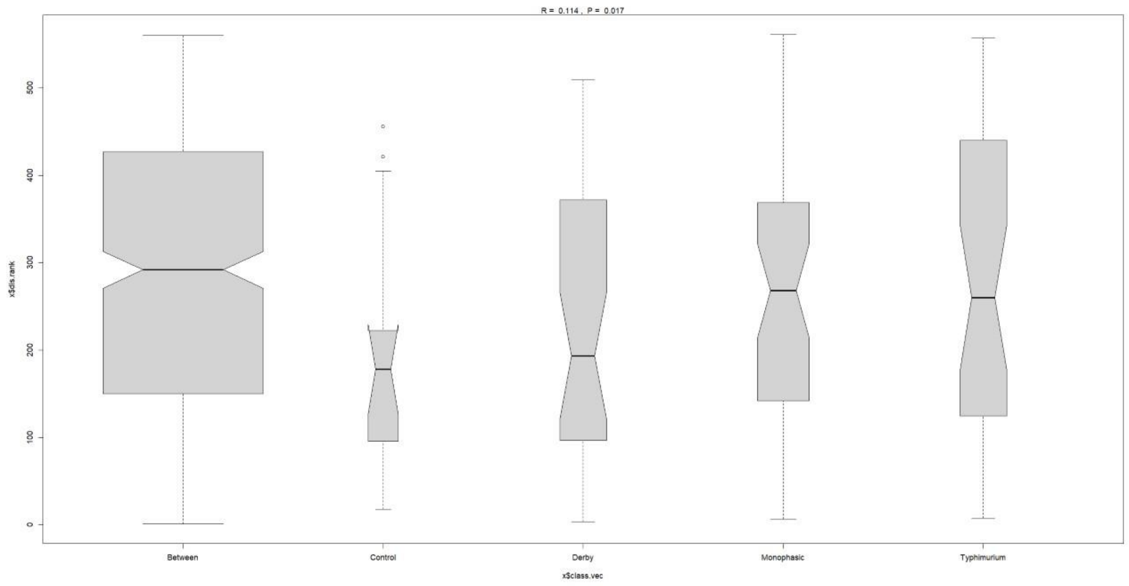


**Supplementary Figure 21 |** Fecal contents (day post-infection - DPI 21) analysis of similarity (ANOSIM) results modeled based on the Bray-Curtis distance (beta-diversity) between samples (response variable) and the experimental treatments (groups) as explanatory variable. A dissimilarity matrix was generated with the vegdist() in R from the Vegan package. In brief, when groups are significantly different ($p < 0.05$ – P refers to *p*-value on the figure) in their microbial community composition, then compositional dissimilarities between the groups is greater than those within the groups (as described here https://www.rdocumentation.org/packages/vegan/versions/2.3-5/topics/anosim). On the y-axis x$dis.rank is the rank of dissimilarity entry. On the x-axis are all individual treatments (groups) and the between groups entry. The higher the ANOSIM statistic R (value closer to 1), the more dissimilar the communities between treatments; and vice-versa (see examples here https://jkzorz.github.io/2019/06/11/ANOSIM-test.html; https://sites.google.com/site/mb3gustame/hypothesis-tests/anosim).

Day 2
Colonic apex
contents

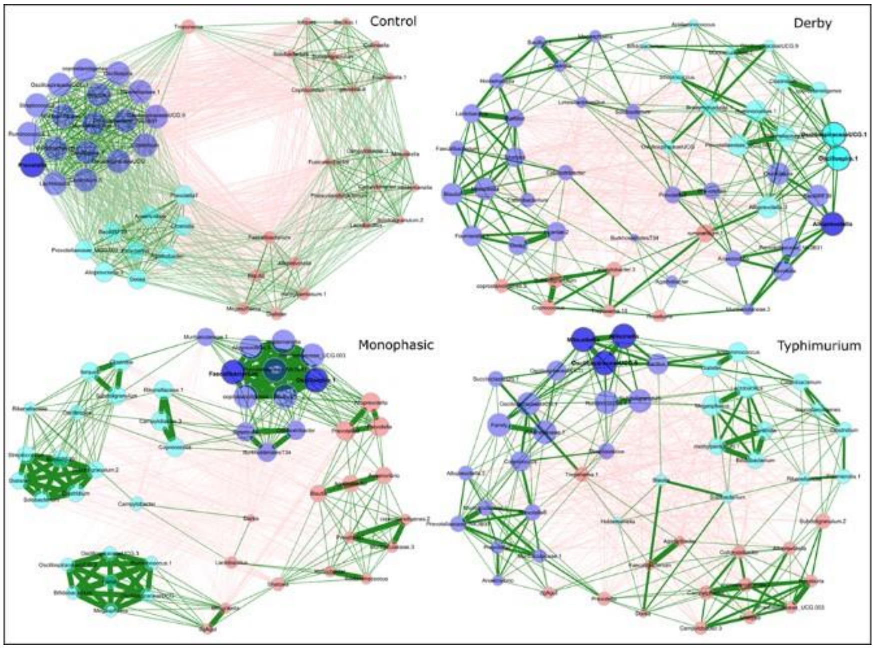


**Supplementary Figure 22 |** Co-occurrence network analysis for DPI 2 colonic apex microbiome across treatments (Control, Derby, Monophasic, and Typhimurium).

Day 28
Colonic apex
contents

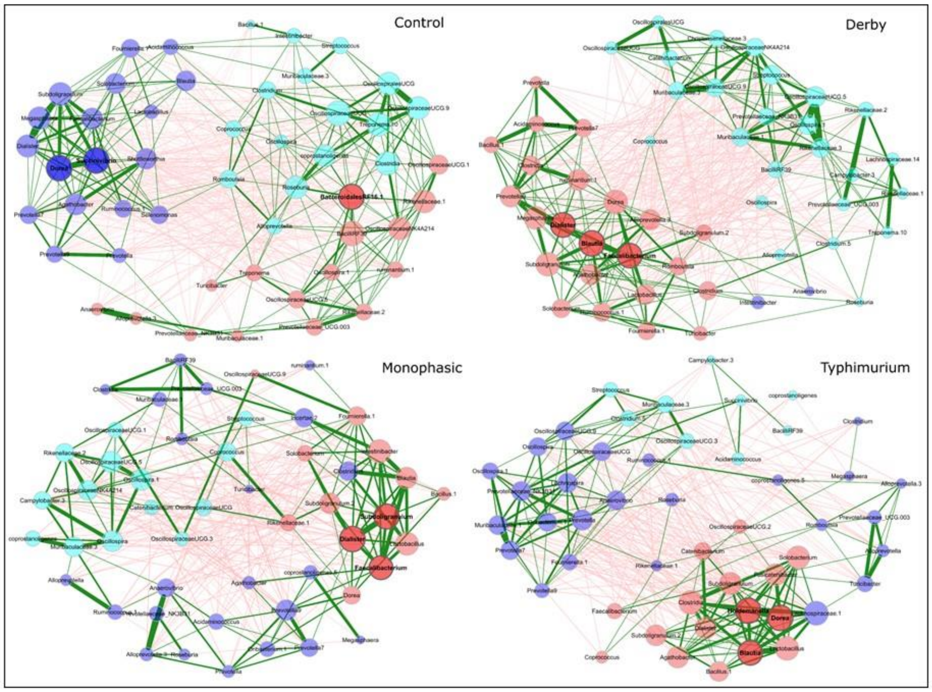


**Supplementary Figure 23 |** Co-occurrence network analysis for DPI 28 colonic apex microbiome across treatments (Control, Derby, Monophasic, and Typhimurium).

Day 2
Fecal
contents

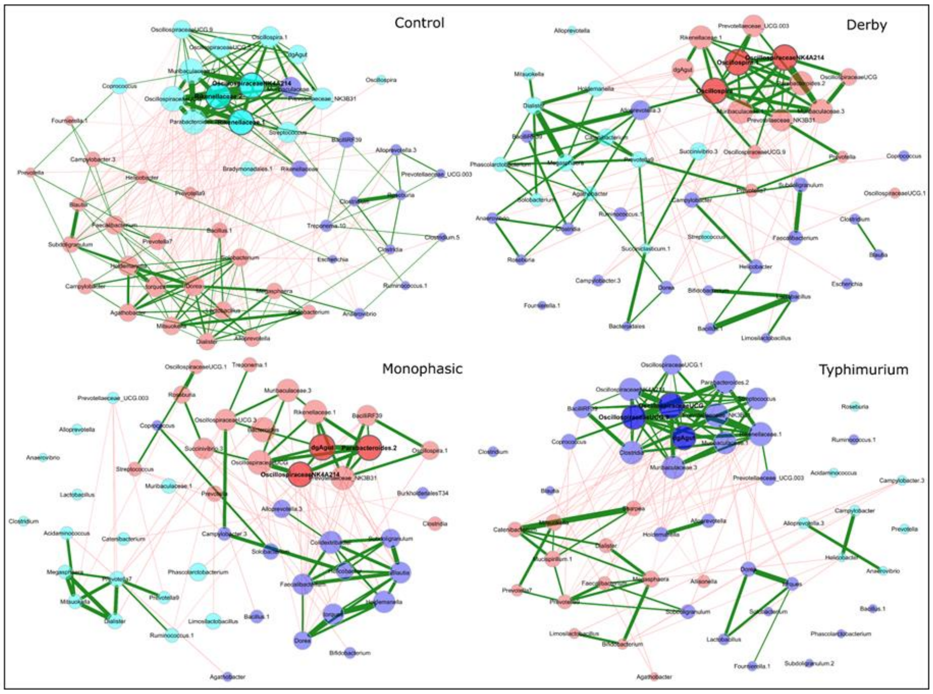


**Supplementary Figure 24 |** Co-occurrence network analysis for DPI 2 fecal microbiome across treatments (Control, Derby, Monophasic, and Typhimurium).

Day 4
Fecal
contents

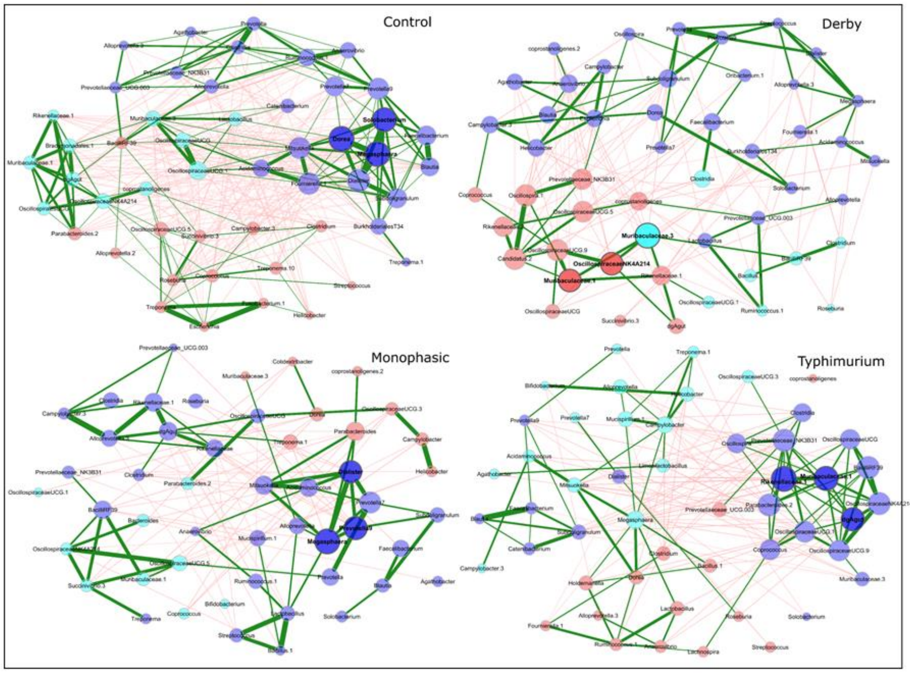


**Supplementary Figure 25 |** Co-occurrence network analysis for DPI 4 fecal microbiome across treatments (Control, Derby, Monophasic, and Typhimurium).

Day 28
Fecal
contents

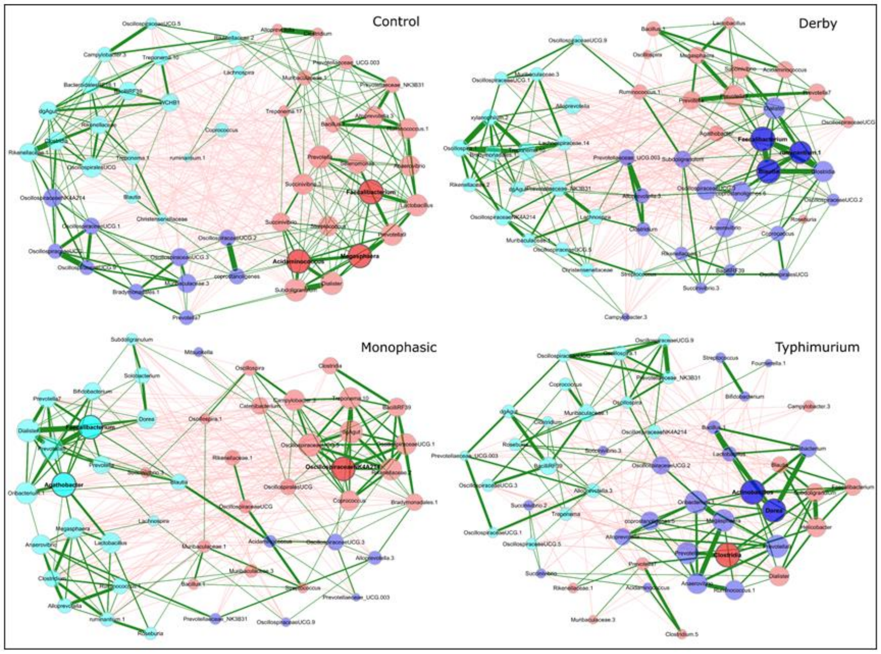


**Supplementary Figure 26 |** Co-occurrence network analysis for DPI 28 fecal microbiome across treatments (Control, Derby, Monophasic, and Typhimurium).

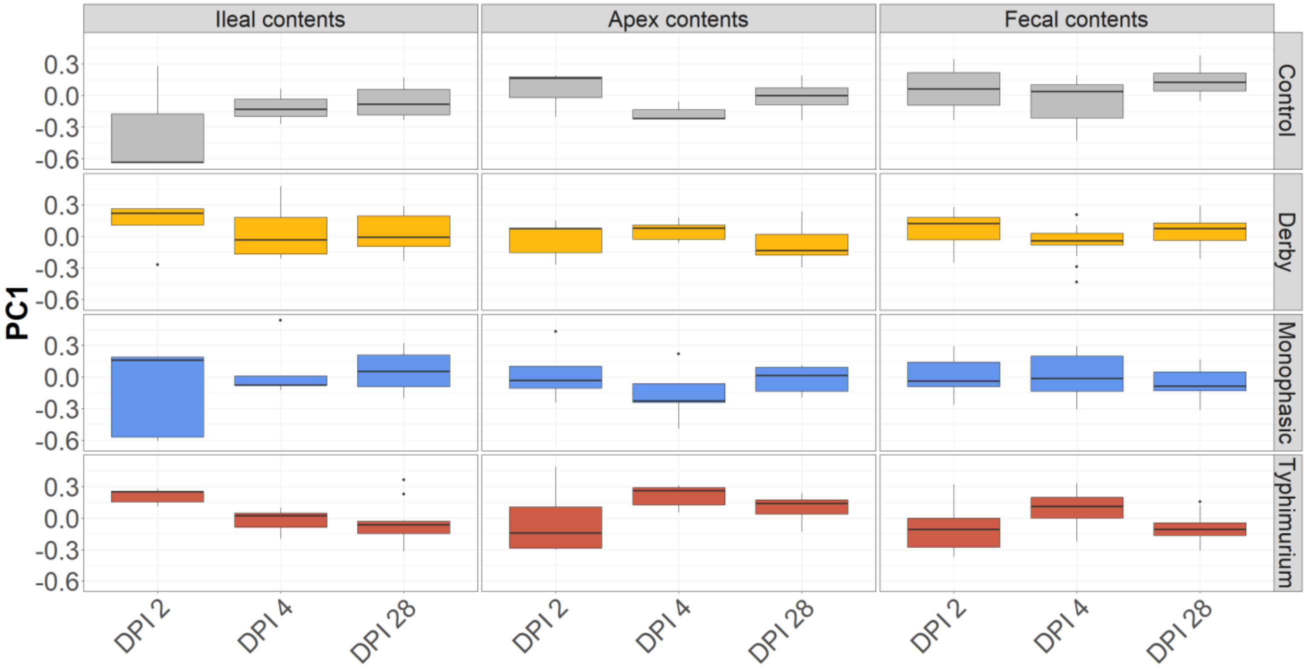


**Supplementary Figure 27 |** Volatility analysis of the microbiome community using distinct sample types across DPI 2, 4, and 28, across treatments (Control, Derby, Monophasic, and Typhimurium). Principal coordinate 1 (PC1) using the Bray Curtis's distance matrix was used to represent the microbiota composition. Sampling is unevenly distributed across DPI and treatments (see Supplementary Figure 1).

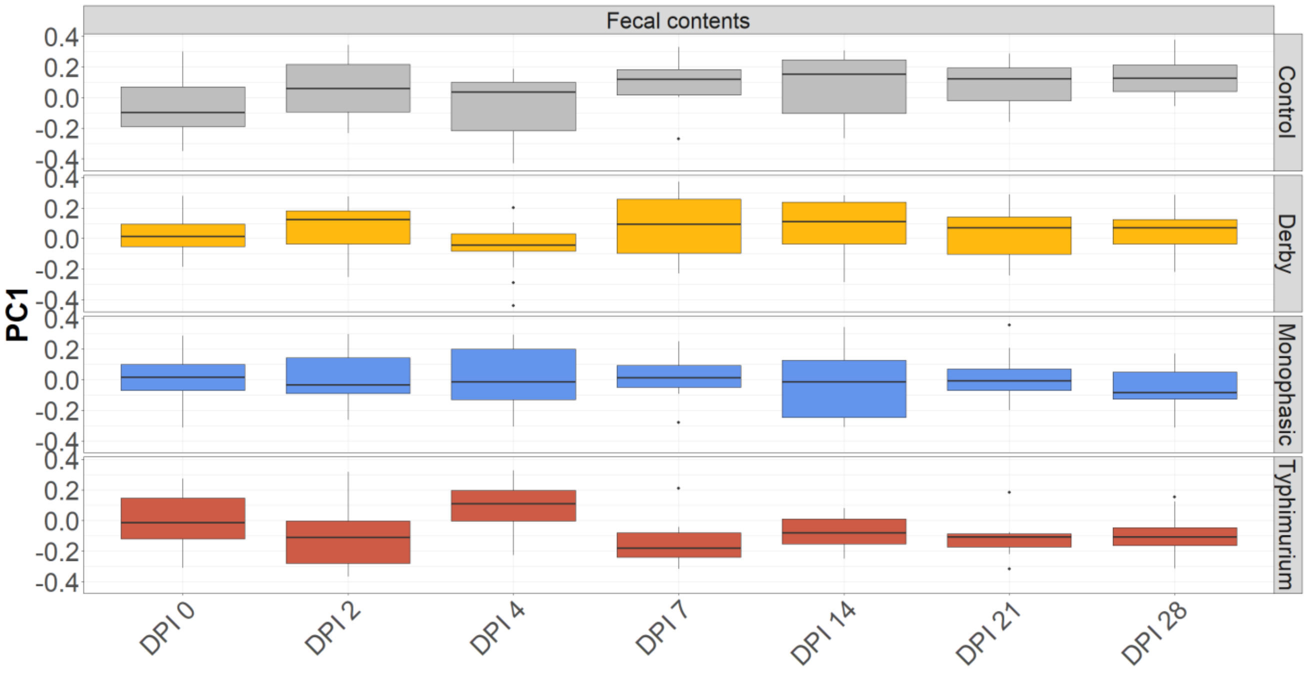


**Supplementary Figure 28 |** Volatility analysis of the fecal microbiome community using distinct sample types across sequential DPI, across treatments (Control, Derby, Monophasic, and Typhimurium). Principal coordinate 1 (PC1) using the Bray Curtis's distance matrix was used to represent the microbiota composition. Sampling is unevenly distributed across DPI and treatments (see **Supplementary Figure 1**).

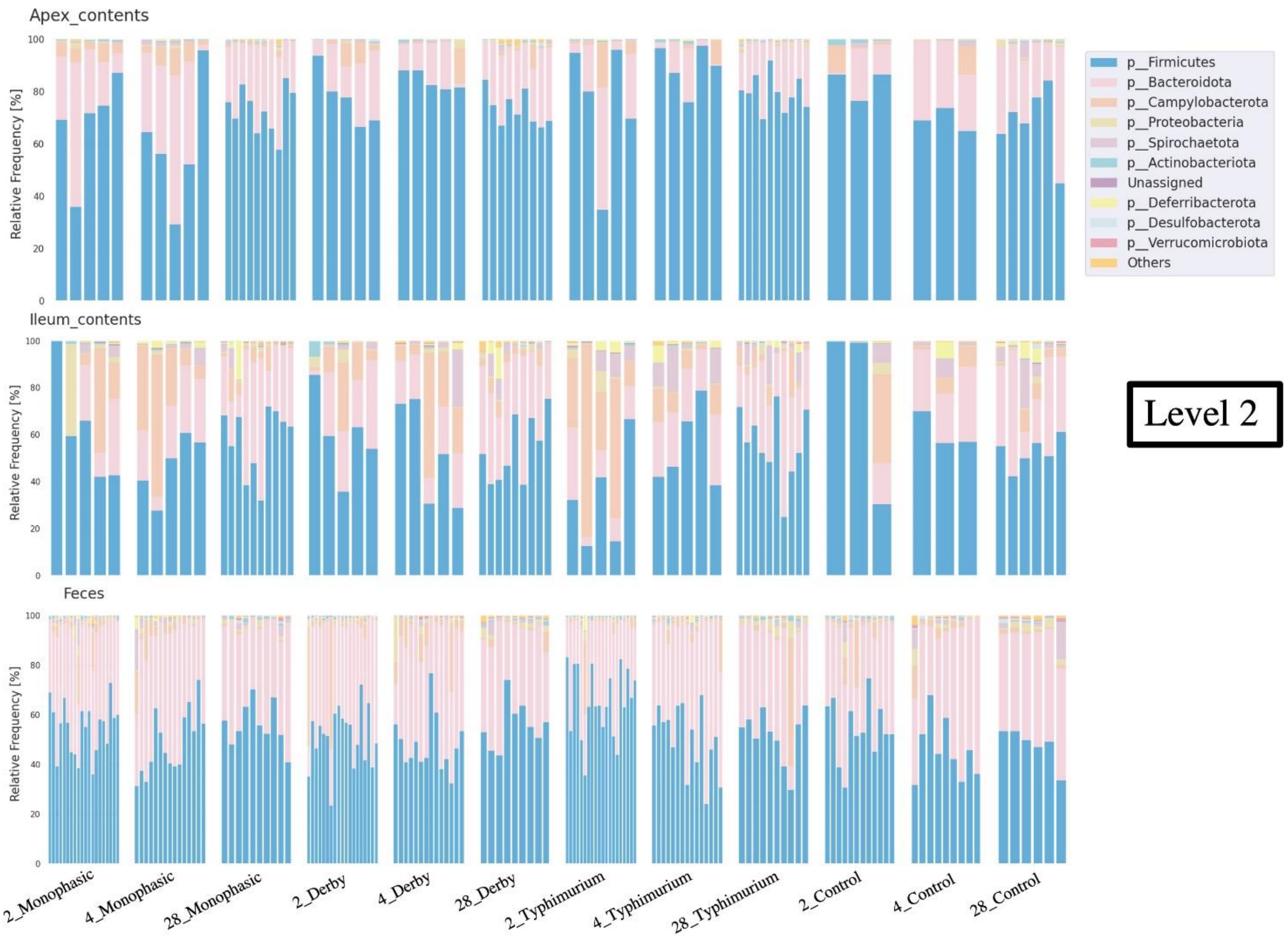


**Supplementary Figure 29 |** Relative frequency of microbiome members at the phylum level (level 2 using QIIME2-based mapping) across samples (colonic apex, ileal, and fecal contents), DPI (2, 4, and 28), and treatments (Control, Derby, Monophasic, and Typhimurium).

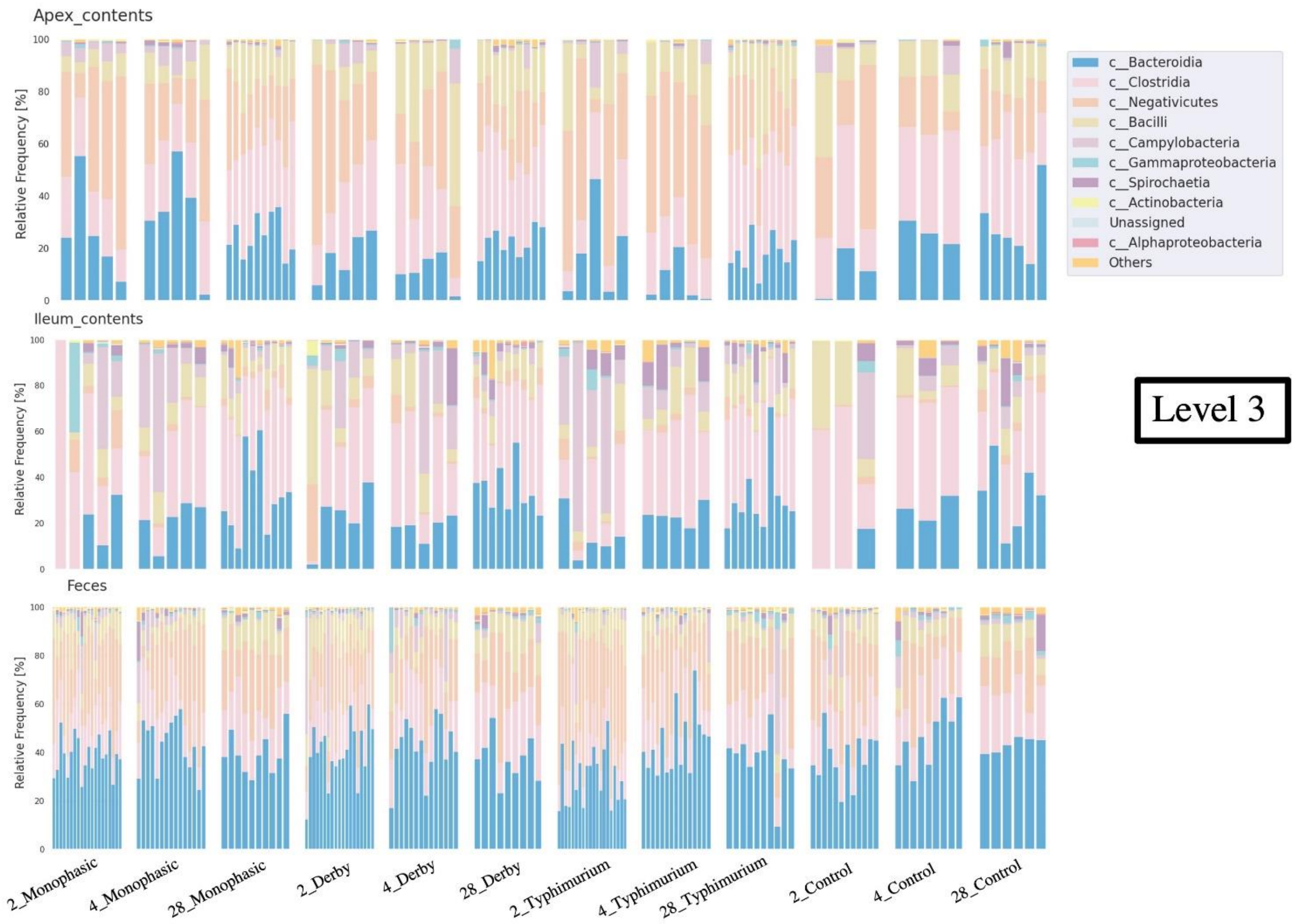


**Supplementary Figure 30 |** Relative frequency of microbiome members at the family level (level 3 using QIIME2-based mapping) across samples (colonic apex, ileal, and fecal contents), DPI (2, 4, and 28), and treatments (Control, Derby, Monophasic, and Typhimurium).

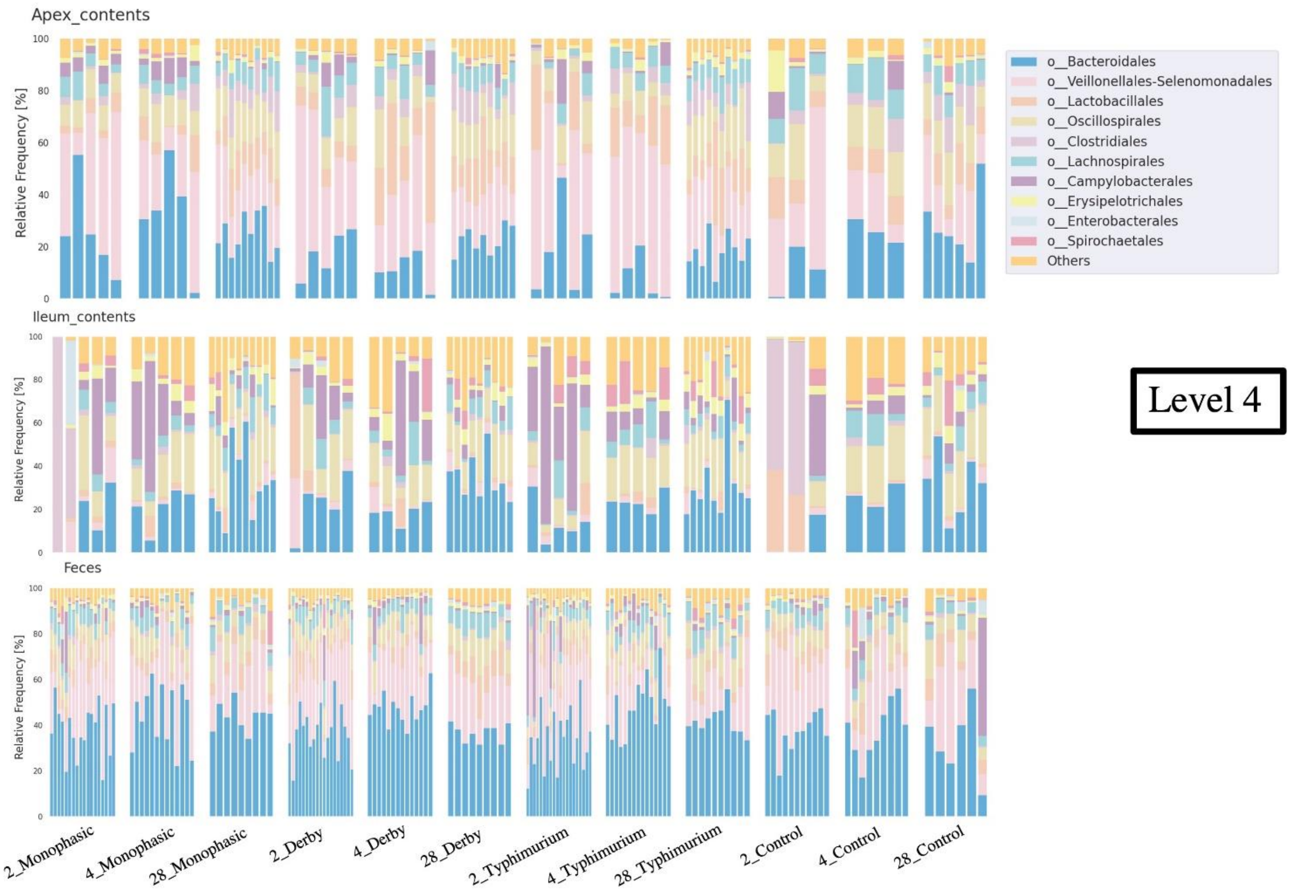


**Supplementary Figure 31 |** Relative frequency of microbiome members at the genus/species levels (level 4 using QIIME2-based mapping) across samples (colonic apex, ileal, and fecal contents), DPI (2, 4, and 28), and treatments (Control, Derby, Monophasic, and Typhimurium).

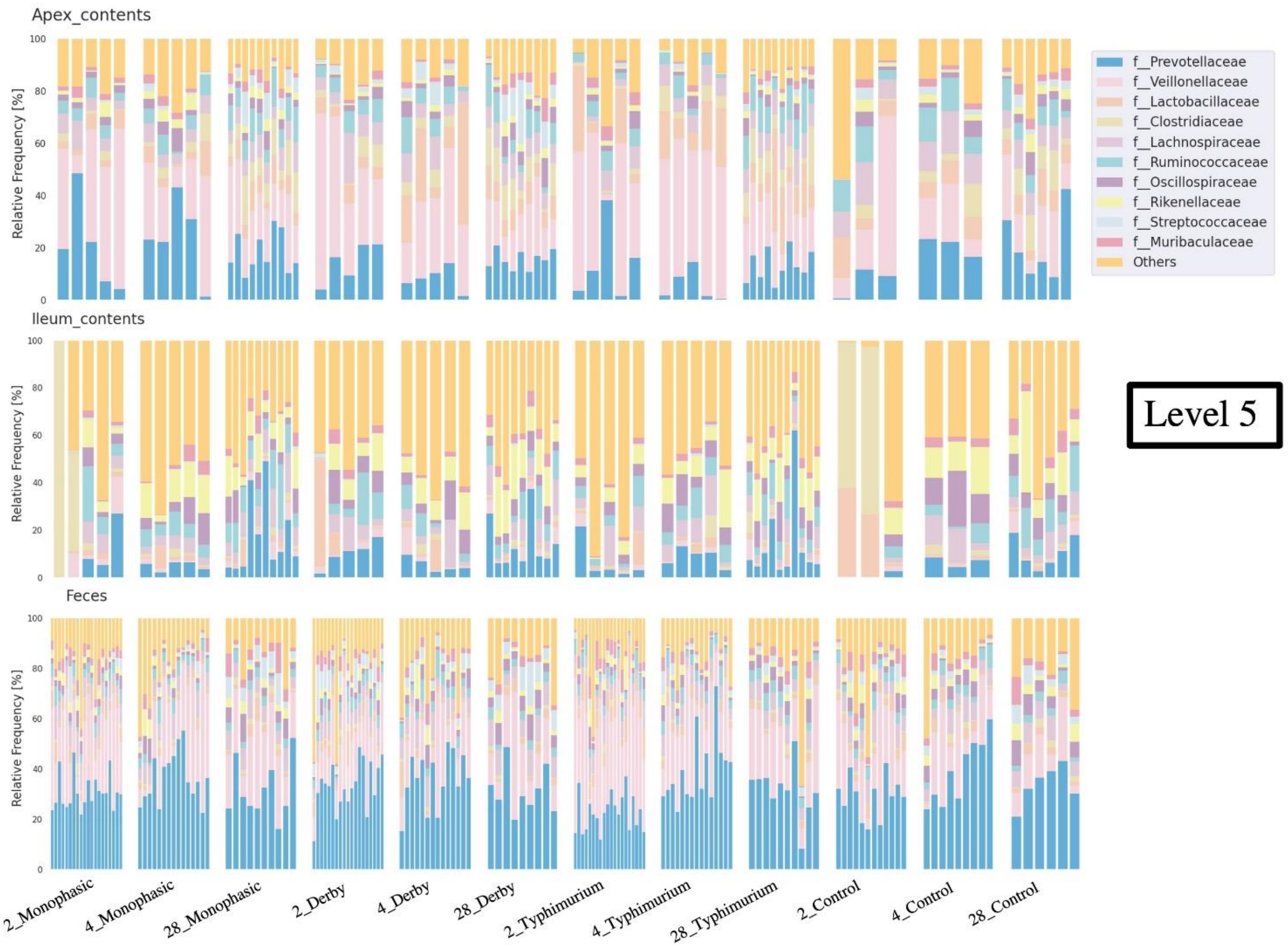


**Supplementary Figure 32 |** Relative frequency of microbiome members at the genus/species levels (level 5 using QIIME2-based mapping) across samples (colonic apex, ileal, and fecal contents), DPI (2, 4, and 28), and treatments (Control, Derby, Monophasic, and Typhimurium).

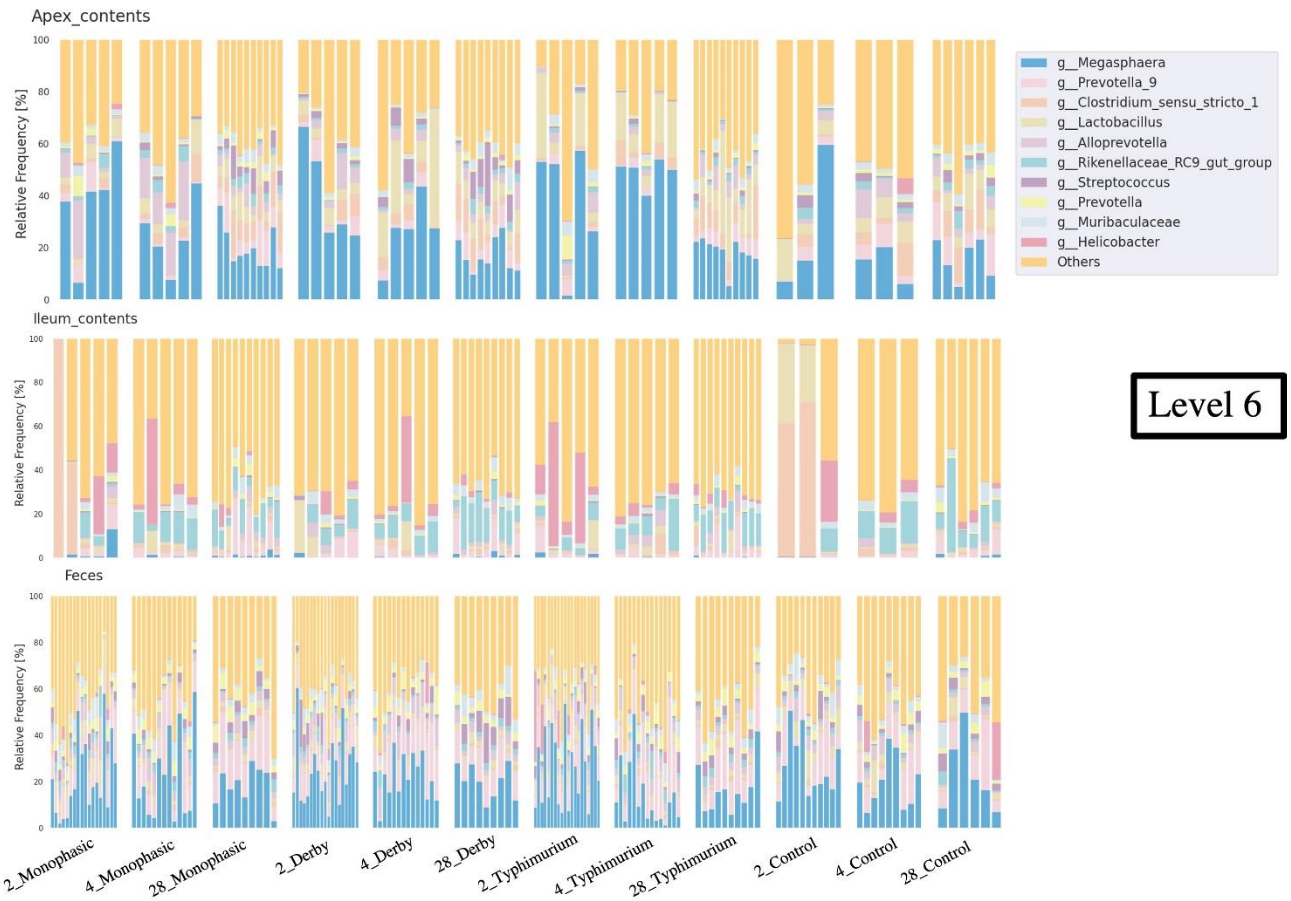


**Supplementary Figure 33 |** Relative frequency of microbiome members at the genus/species levels (level 6 using QIIME2-based mapping) across samples (colonic apex, ileal, and fecal contents), DPI (2, 4, and 28), and treatments (Control, Derby, Monophasic, and Typhimurium).

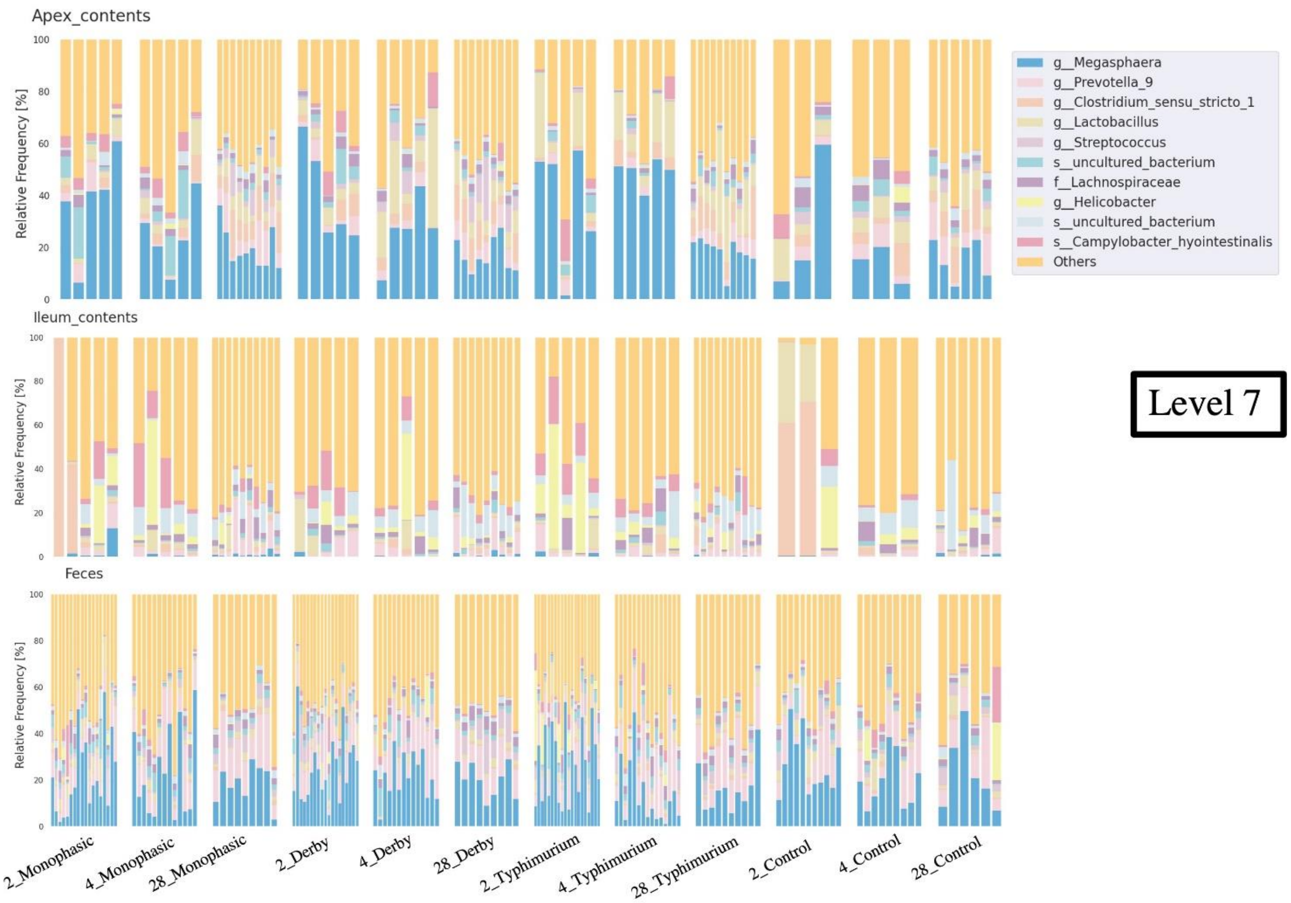


**Supplementary Figure 34 |** Relative frequency of microbiome members at the genus/species levels (level 7 using QIIME2-based mapping) across samples (colonic apex, ileal, and fecal contents), DPI (2, 4, and 28), and treatments (Control, Derby, Monophasic, and Typhimurium).

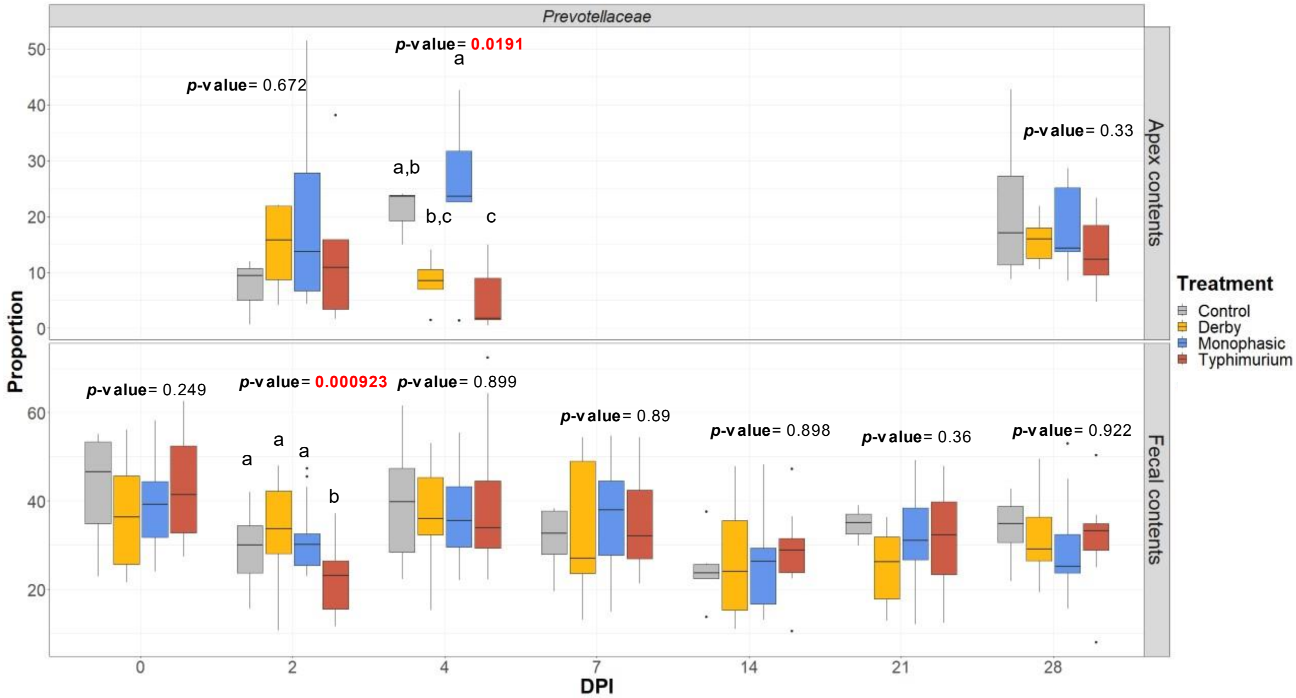


**Supplementary Figure 35 |** *Prevotellaceae* proportion across treatments (Control, Derby, Monophasic, and Typhimurium) and DPI for both colonic apex (apex) and fecal contents. Statistical analysis was done using an ANOVA followed by a pairwise T-test ($p < 0.05$). Different superscript letters indicate significant differences between treatments. Only animals that had microbiome samples passed through the bioinformatic cut-off for quality control were included in this analysis.

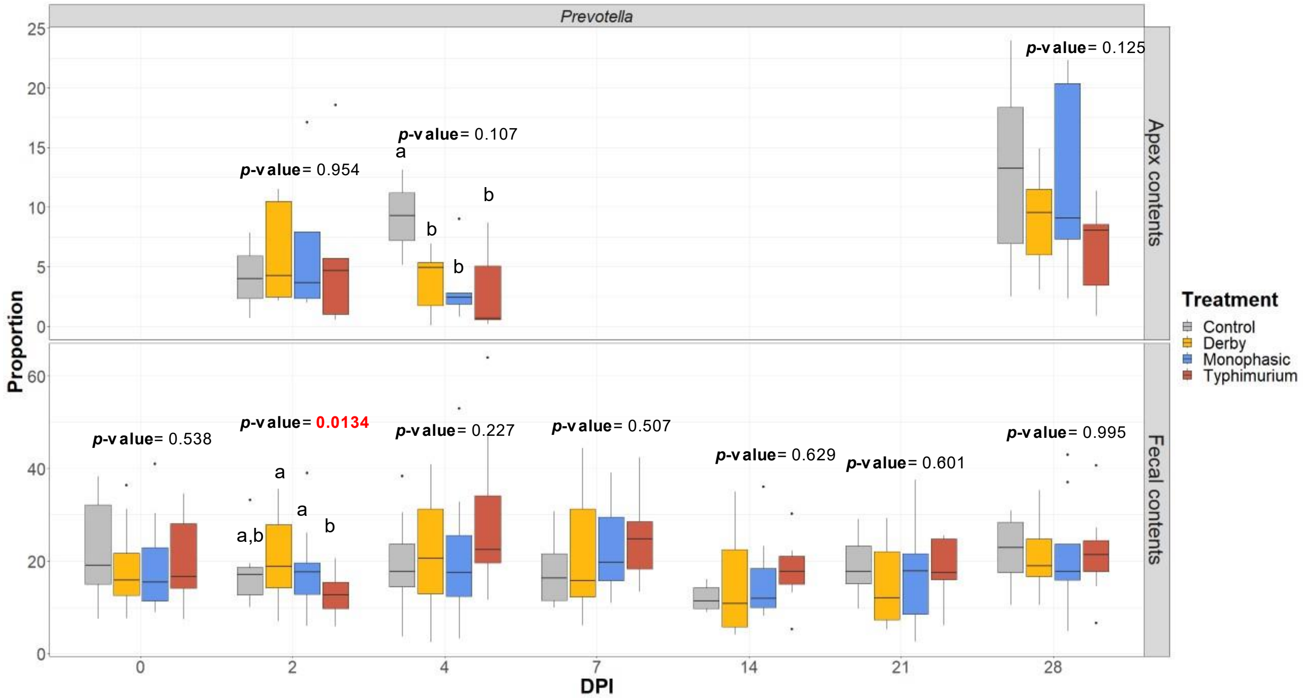


**Supplementary Figure 36 |** *Prevotella* proportion across treatments (Control, Derby, Monophasic, and Typhimurium) and DPI for both colonic apex (apex) and fecal contents. Statistical analysis was done using an ANOVA followed by a pairwise T-test ($p < 0.05$). Different superscript letters indicate significant differences between treatments. Only animals that had microbiome samples passed through the bioinformatic cut-off for quality control were included in this analysis.

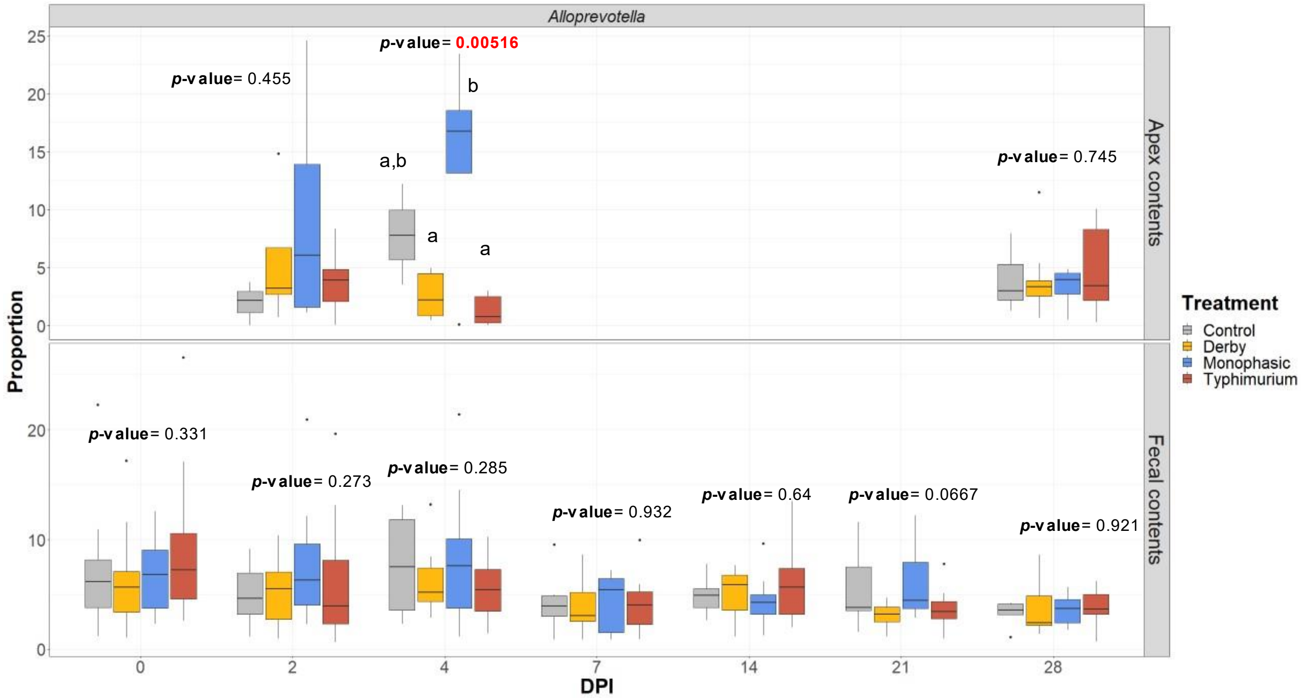


**Supplementary Figure 37 |** *Alloprevotella* proportion across treatments (Control, Derby, Monophasic, and Typhimurium) and DPI for both colonic apex (apex) and fecal contents. Statistical analysis was done using an ANOVA followed by a pairwise T-test ($p < 0.05$). Different superscript letters indicate significant differences between treatments. Only animals that had microbiome samples passed through the bioinformatic cut-off for quality control were included in this analysis.

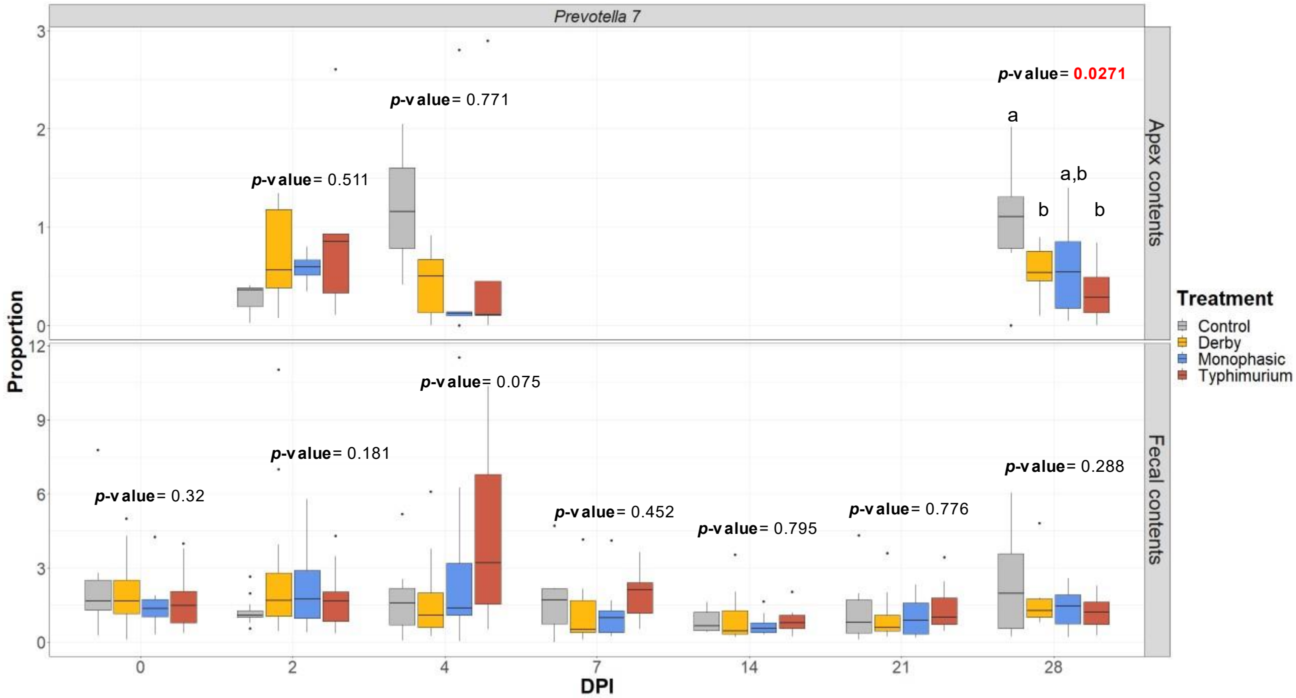


**Supplementary Figure 38 |** *Prevotella 7* proportion across treatments (Control, Derby, Monophasic, and Typhimurium) and DPI for both colonic apex (apex) and fecal contents. Statistical analysis was done using an ANOVA followed by a pairwise T-test ($p < 0.05$). Different superscript letters indicate significant differences between treatments. Only animals that had microbiome samples passed through the bioinformatic cut-off for quality control were included in this analysis.

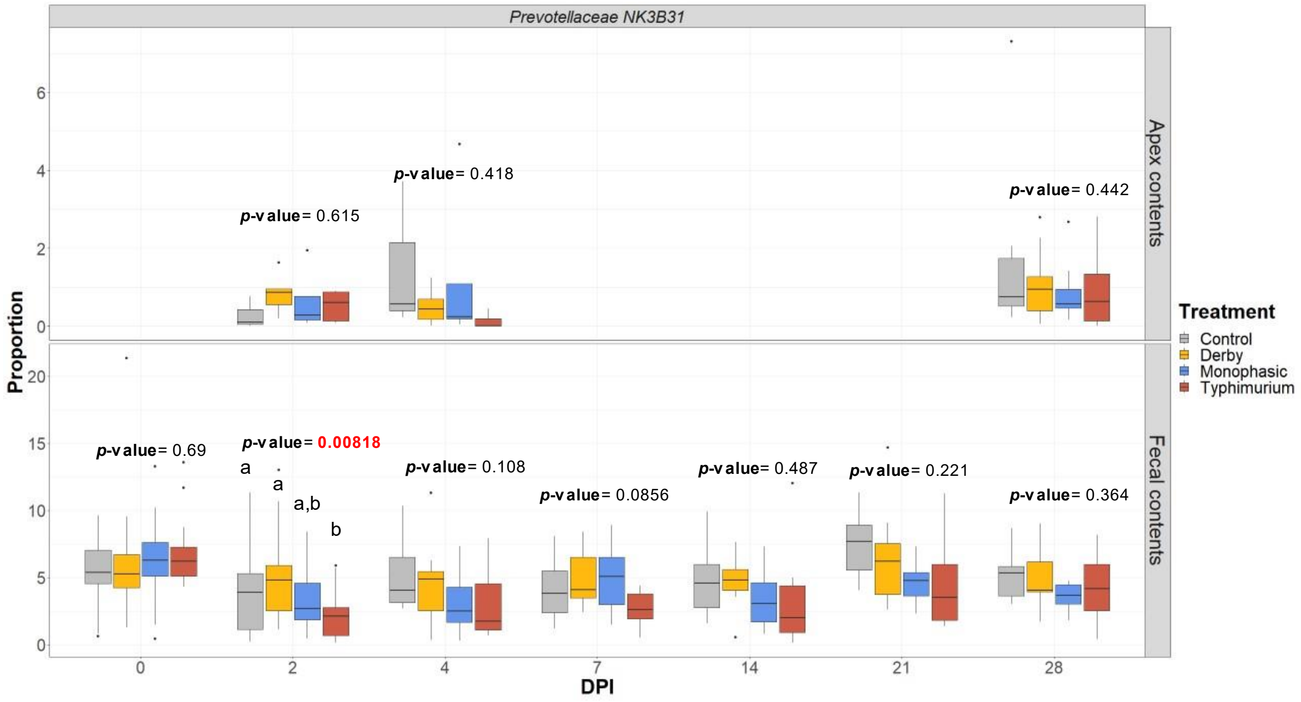


**Supplementary Figure 39 |** *Prevotellaceae NK3B31* proportion across treatments (Control, Derby, Monophasic, and Typhimurium) and DPI for both colonic apex (apex) and fecal contents. Statistical analysis was done using an ANOVA followed by a pairwise T-test ($p < 0.05$). Different superscript letters indicate significant differences between treatments. Only animals that had microbiome samples passed through the bioinformatic cut-off for quality control were included in this analysis.

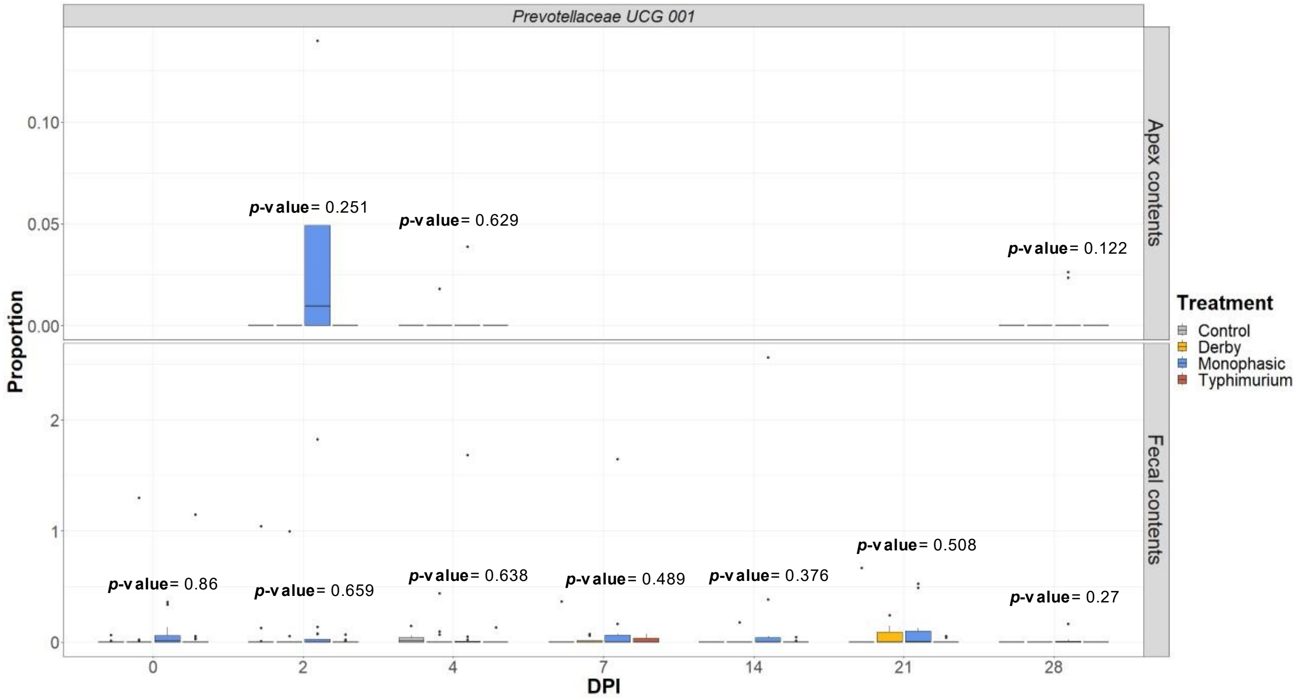


**Supplementary Figure 40 |** *Prevotellaceae UCG 001* proportion across treatments (Control, Derby, Monophasic, and Typhimurium) and DPI for both colonic apex (apex) and fecal contents. Statistical analysis was done using an ANOVA followed by a pairwise T-test ($p < 0.05$). Different superscript letters indicate significant differences between treatments. Only animals that had microbiome samples passed through the bioinformatic cut-off for quality control were included in this analysis.

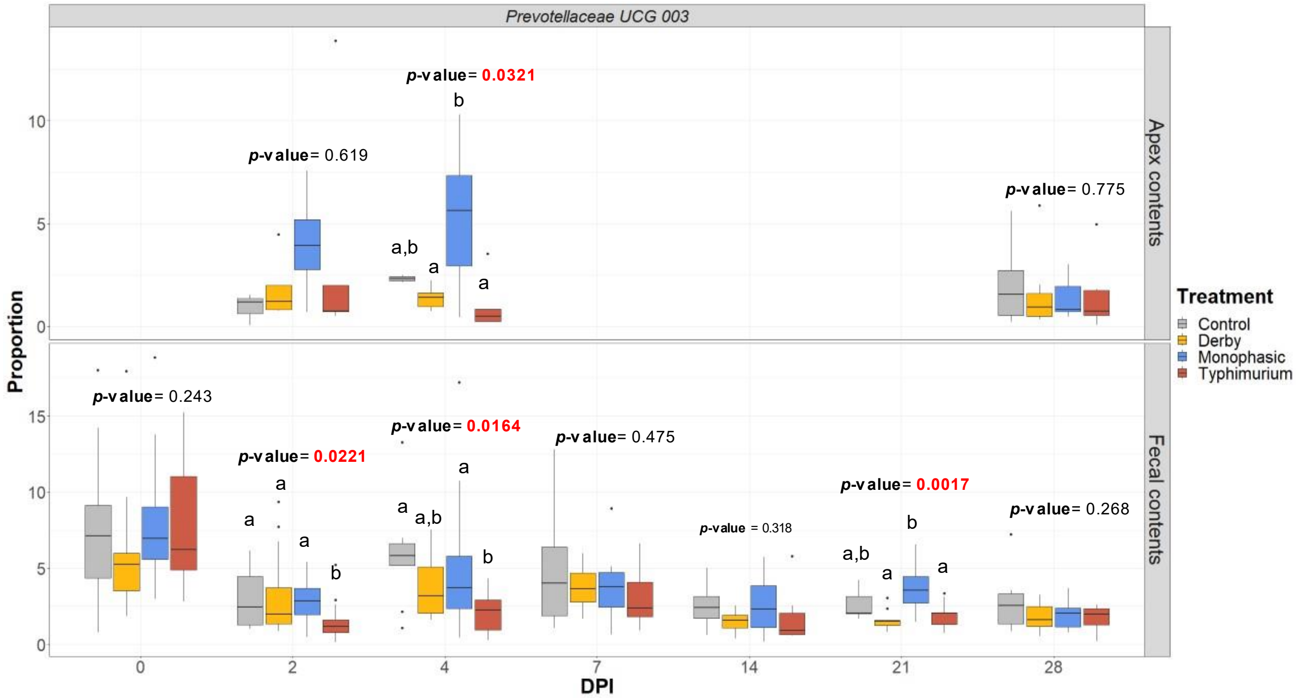


**Supplementary Figure 41** | *Prevotellaceae UCG 003* proportion across treatments (Control, Derby, Monophasic, and Typhimurium) and DPI for both colonic apex (apex) and fecal contents. Statistical analysis was done using an ANOVA followed by a pairwise T-test ($p < 0.05$). Different superscript letters indicate significant differences between treatments. Only animals that had microbiome samples passed through the bioinformatic cut-off for quality control were included in this analysis.

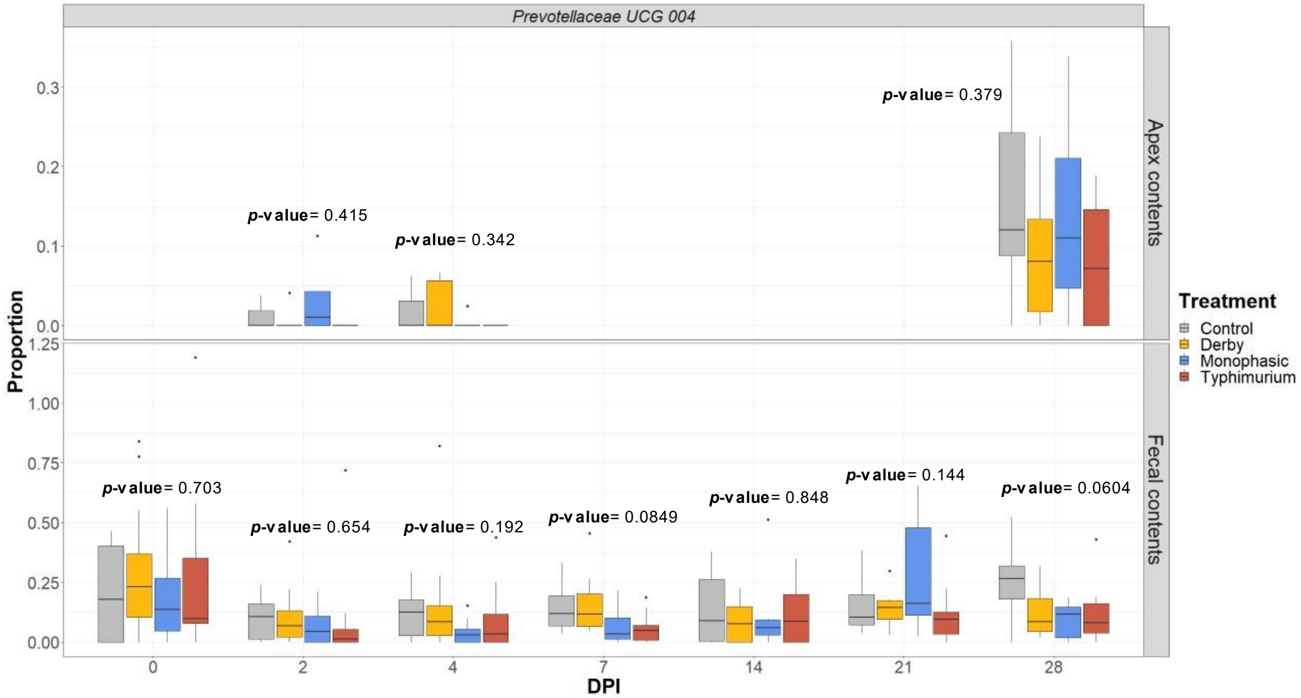


**Supplementary Figure 42 |** *Prevotellaceae UCG 004* proportion across treatments (Control, Derby, Monophasic, and Typhimurium) and DPI for both colonic apex (apex) and fecal contents. Statistical analysis was done using an ANOVA followed by a pairwise T-test ($p < 0.05$). Different superscript letters indicate significant differences between treatments. Only animals that had microbiome samples passed through the bioinformatic cut-off for quality control were included in this analysis.

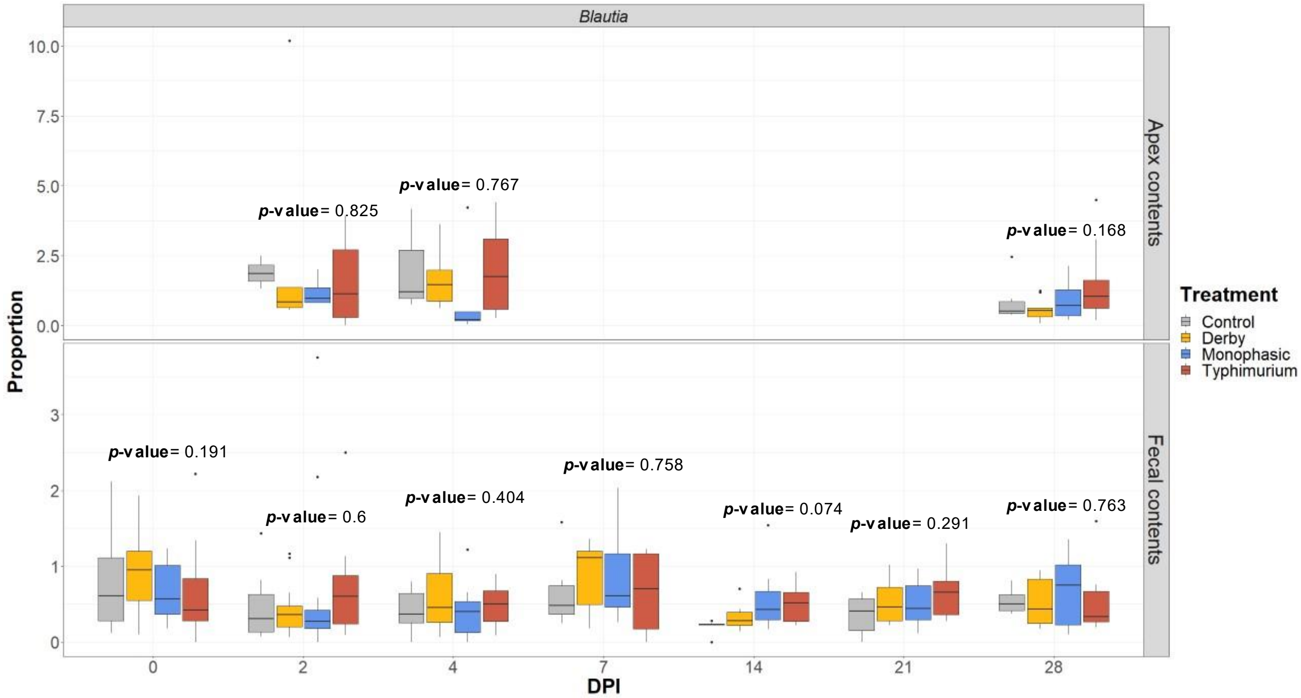


**Supplementary Figure 43 |** *Blautia* proportion across treatments (Control, Derby, Monophasic, and Typhimurium) and DPI for both colonic apex (apex) and fecal contents. Statistical analysis was done using an ANOVA followed by a pairwise T-test ($p < 0.05$). Different superscript letters indicate significant differences between treatments. Only animals that had microbiome samples passed through the bioinformatic cut-off for quality control were included in this analysis.

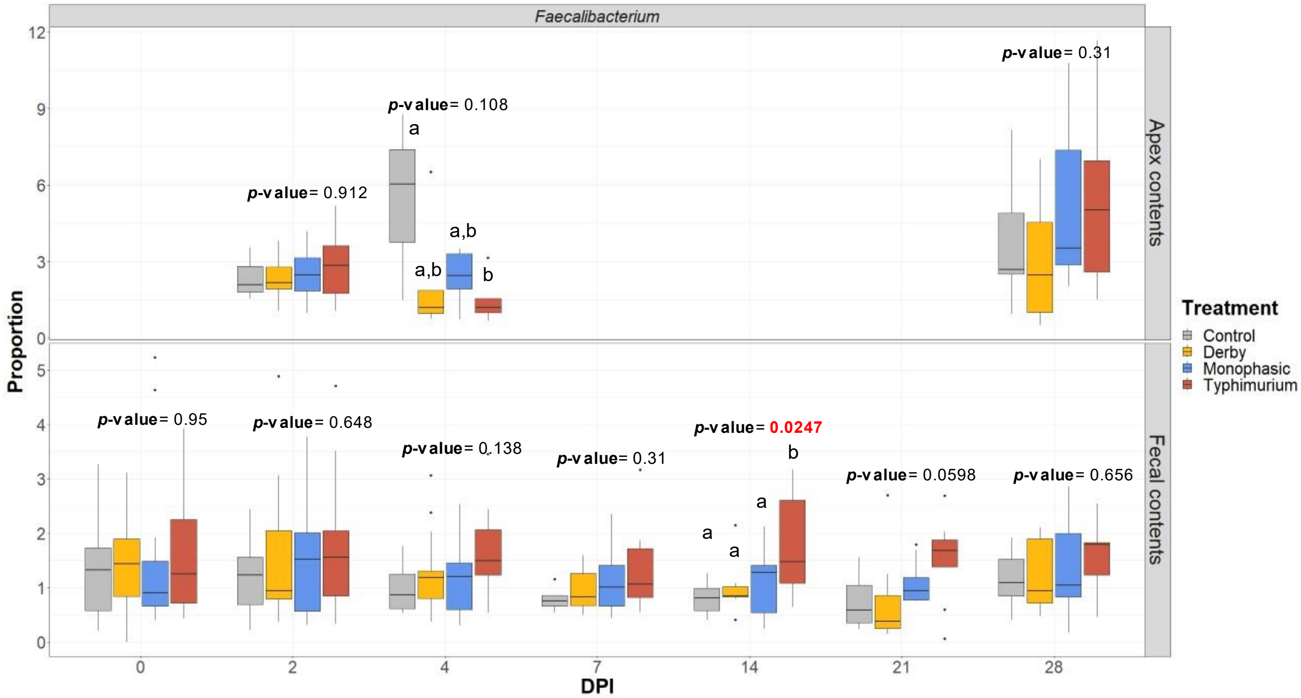


**Supplementary Figure 44 |** *Faecalibacterium* proportion across treatments (Control, Derby, Monophasic, and Typhimurium) and DPI for both colonic apex (apex) and fecal contents. Statistical analysis was done using an ANOVA followed by a pairwise T-test ($p < 0.05$). Different superscript letters indicate significant differences between treatments. Only animals that had microbiome samples passed through the bioinformatic cut-off for quality control were included in this analysis.

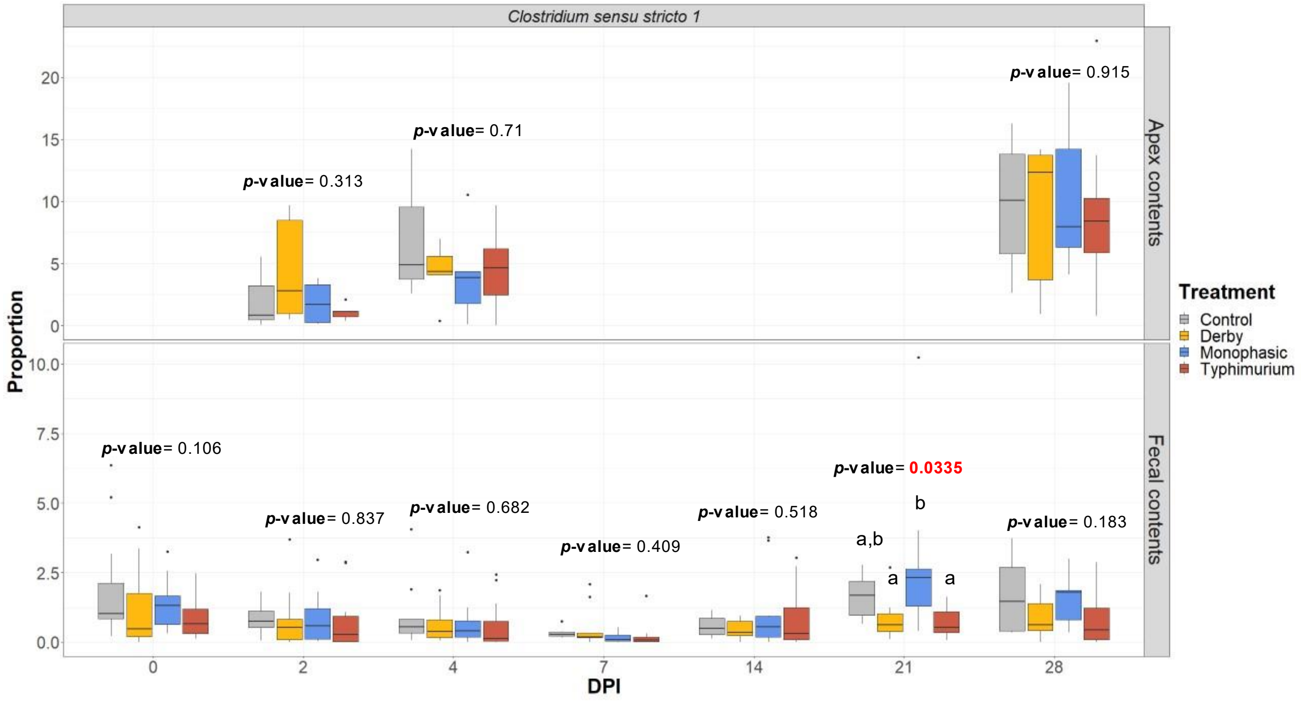


**Supplementary Figure 45 |** *Clostridium sensu stricto 1* proportion across treatments (Control, Derby, Monophasic, and Typhimurium) and DPI for both colonic apex (apex) and fecal contents. Statistical analysis was done using an ANOVA followed by a pairwise T-test ($p < 0.05$). Different superscript letters indicate significant differences between treatments. Only animals that had microbiome samples passed through the bioinformatic cut-off for quality control were included in this analysis.

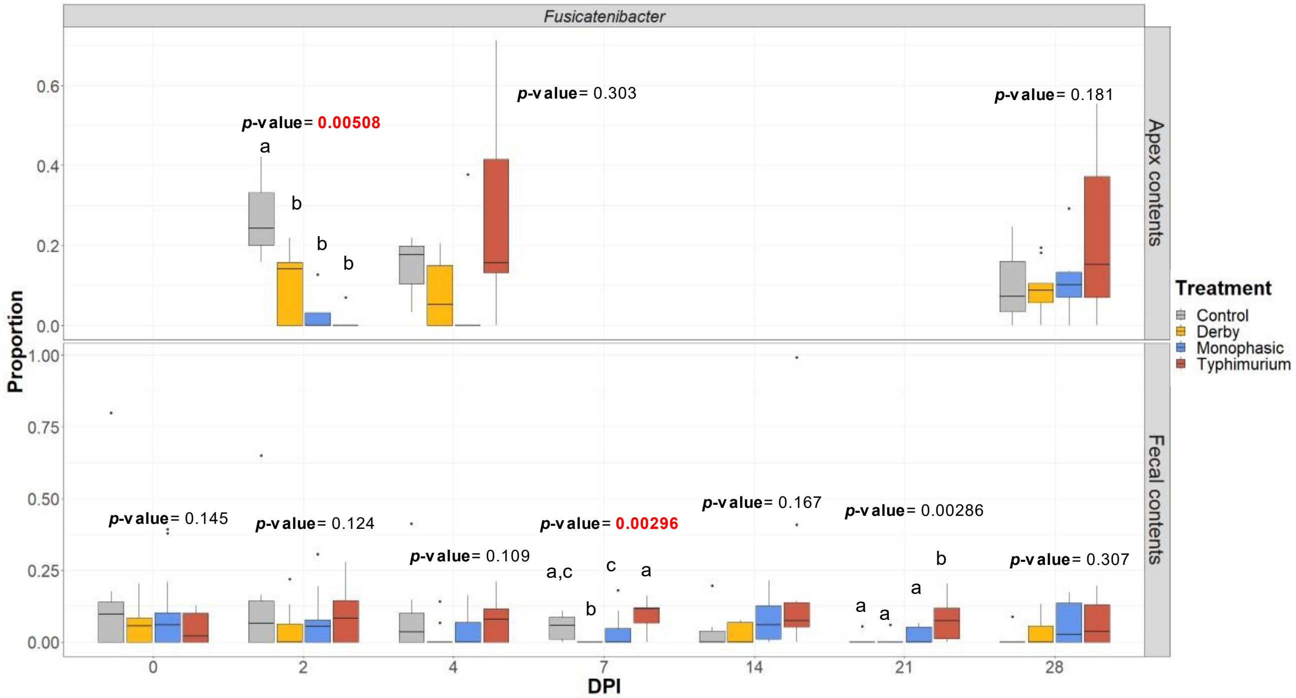


**Supplementary Figure 46 |** *Fusicatenibacter* proportion across treatments (Control, Derby, Monophasic, and Typhimurium) and DPI for both colonic apex (apex) and fecal contents. Statistical analysis was done using an ANOVA followed by a pairwise T-test ($p < 0.05$). Different superscript letters indicate significant differences between treatments. Only animals that had microbiome samples passed through the bioinformatic cut-off for quality control were included in this analysis.

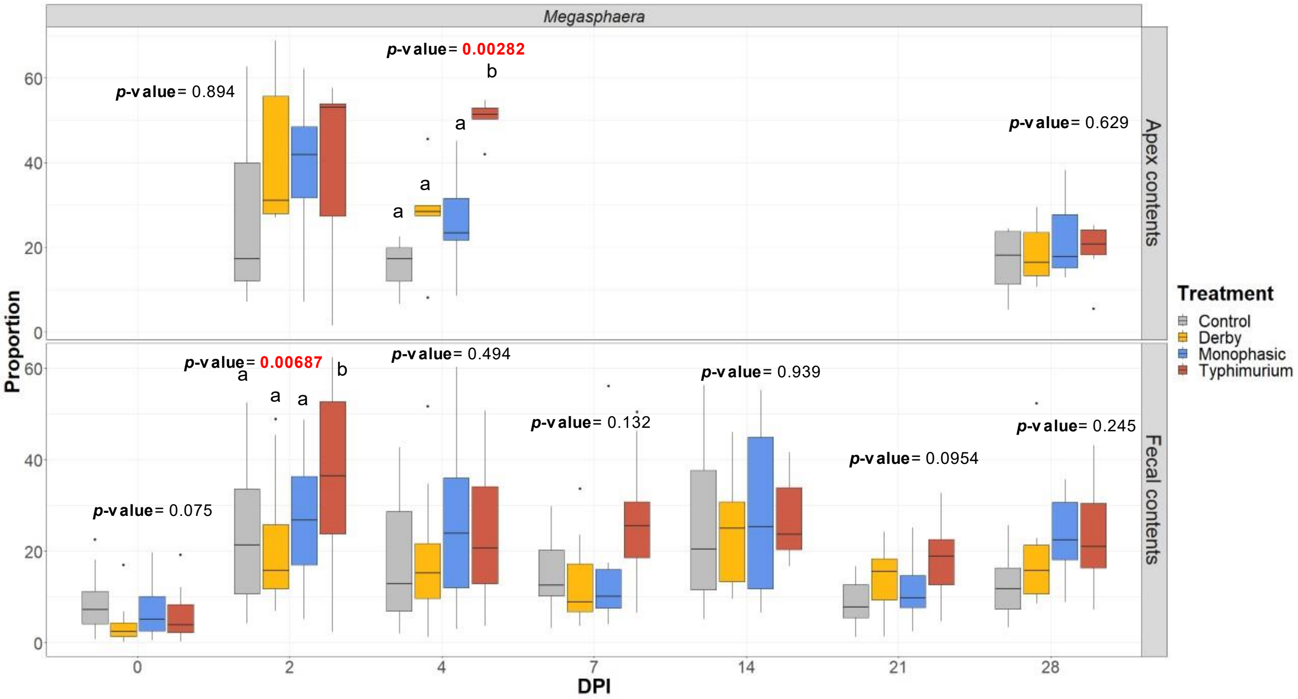


**Supplementary Figure 47 |** *Megasphaera* proportion across treatments (Control, Derby, Monophasic, and Typhimurium) and DPI for both colonic apex (apex) and fecal contents. Statistical analysis was done using an ANOVA followed by a pairwise T-test ($p < 0.05$). Different superscript letters indicate significant differences between treatments. Only animals that had microbiome samples passed through the bioinformatic cut-off for quality control were included in this analysis.

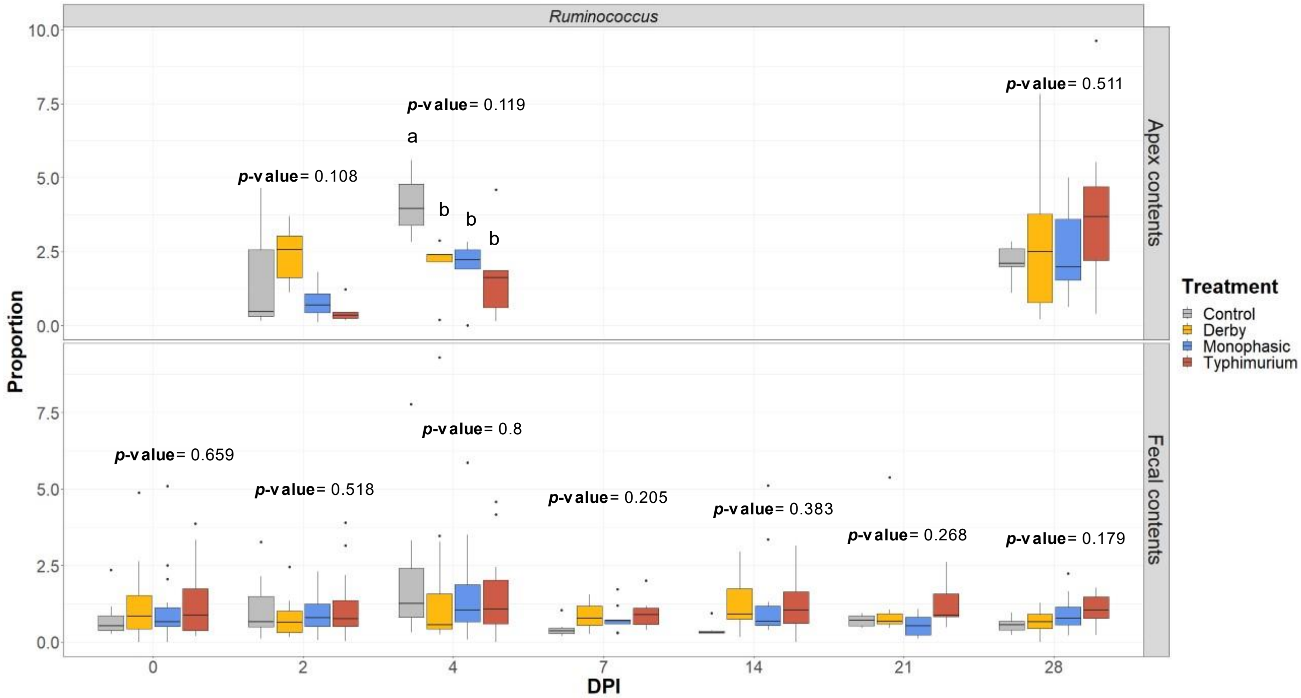


**Supplementary Figure 48 |** *Ruminococcus* proportion across treatments (Control, Derby, Monophasic, and Typhimurium) and DPI for both colonic apex (apex) and fecal contents. Statistical analysis was done using an ANOVA followed by a pairwise T-test ($p < 0.05$). Different superscript letters indicate significant differences between treatments. Only animals that had microbiome samples passed through the bioinformatic cut-off for quality control were included in this analysis.

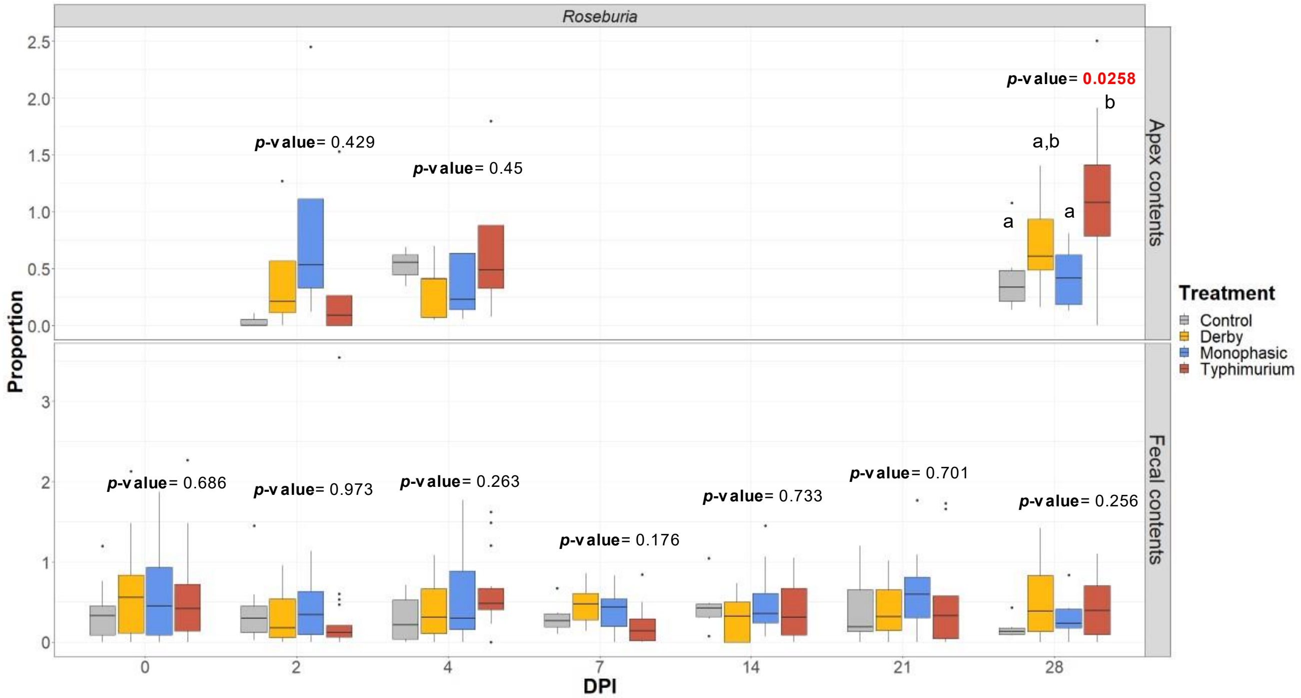


**Supplementary Figure 49 |** *Roseburia* proportion across treatments (Control, Derby, Monophasic, and Typhimurium) and DPI for both colonic apex (apex) and fecal contents. Statistical analysis was done using an ANOVA followed by a pairwise T-test ($p < 0.05$). Different superscript letters indicate significant differences between treatments. Only animals that had microbiome samples passed through the bioinformatic cut-off for quality control were included in this analysis.

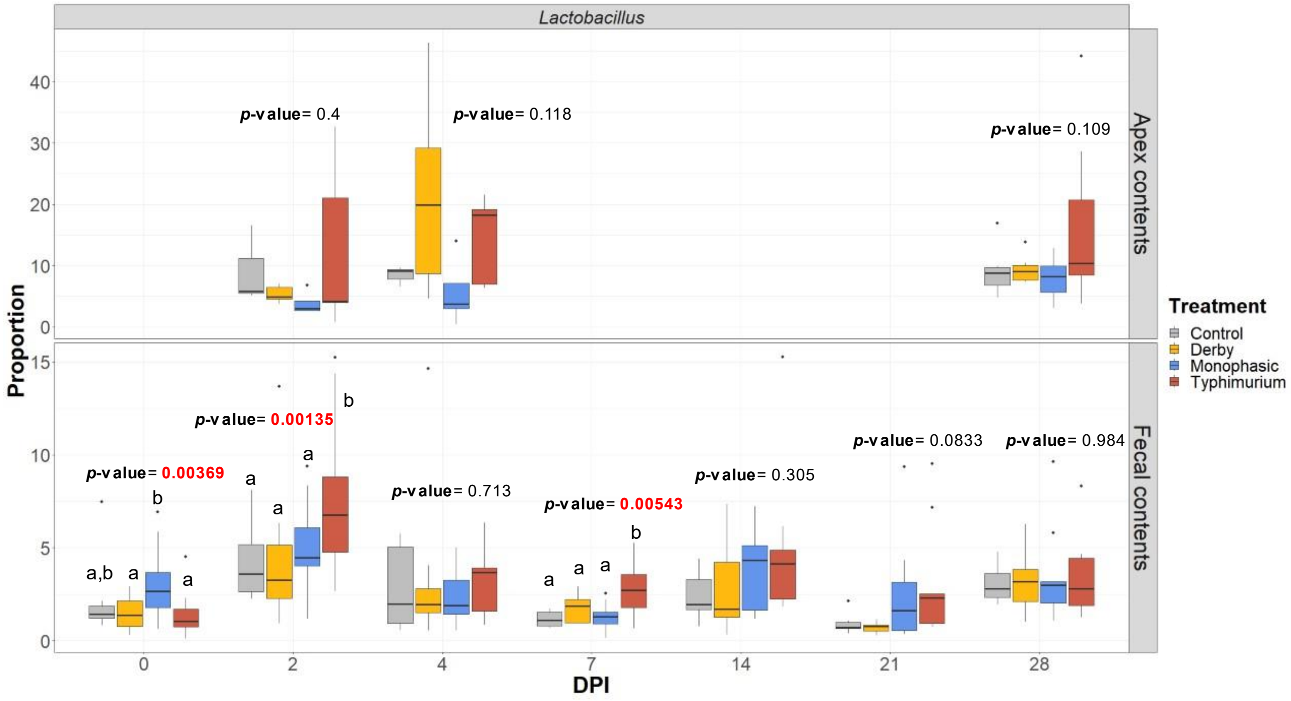


**Supplementary Figure 50 |** *Lactobacillus* proportion across treatments (Control, Derby, Monophasic, and Typhimurium) and DPI for both colonic apex (apex) and fecal contents. Statistical analysis was done using an ANOVA followed by a pairwise T-test ($p < 0.05$). Different superscript letters indicate significant differences between treatments. Only animals that had microbiome samples passed through the bioinformatic cut-off for quality control were included in this analysis.

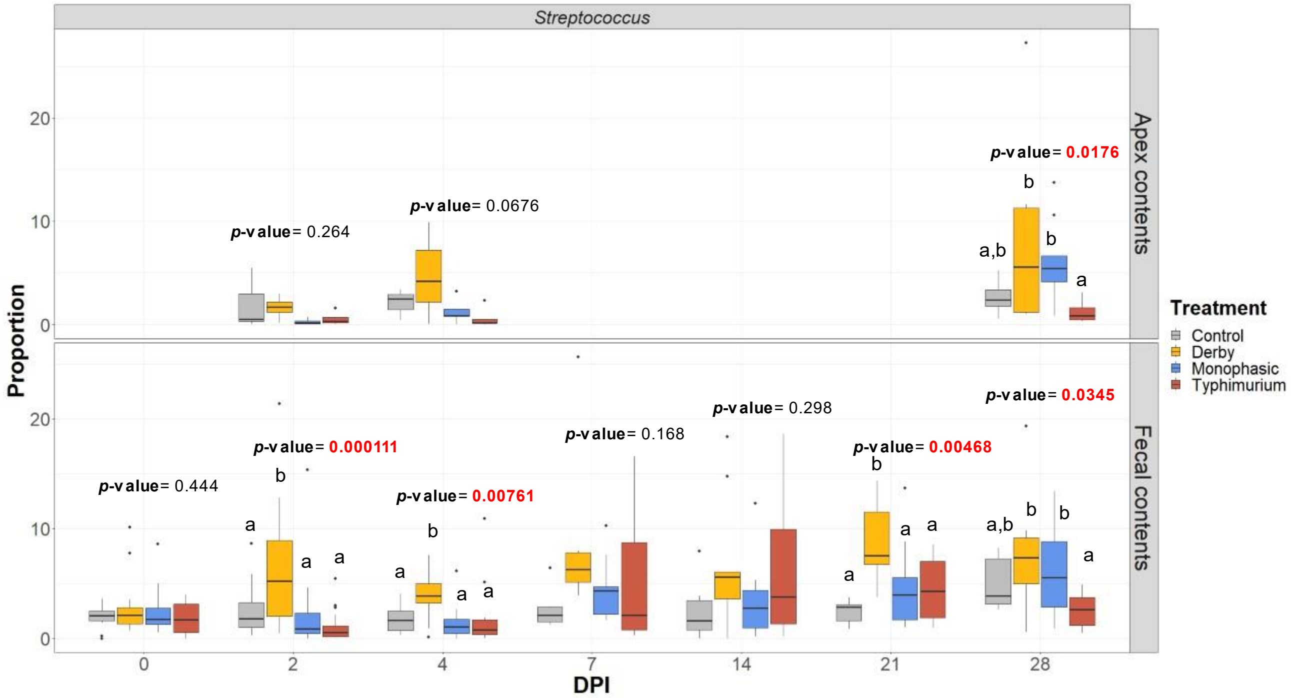


**Supplementary Figure 51 |** *Streptococcus* proportion across treatments (Control, Derby, Monophasic, and Typhimurium) and DPI for both colonic apex (apex) and fecal contents. Statistical analysis was done using an ANOVA followed by a pairwise T-test ($p < 0.05$). Different superscript letters indicate significant differences between treatments. Only animals that had microbiome samples passed through the bioinformatic cut-off for quality control were included in this analysis.

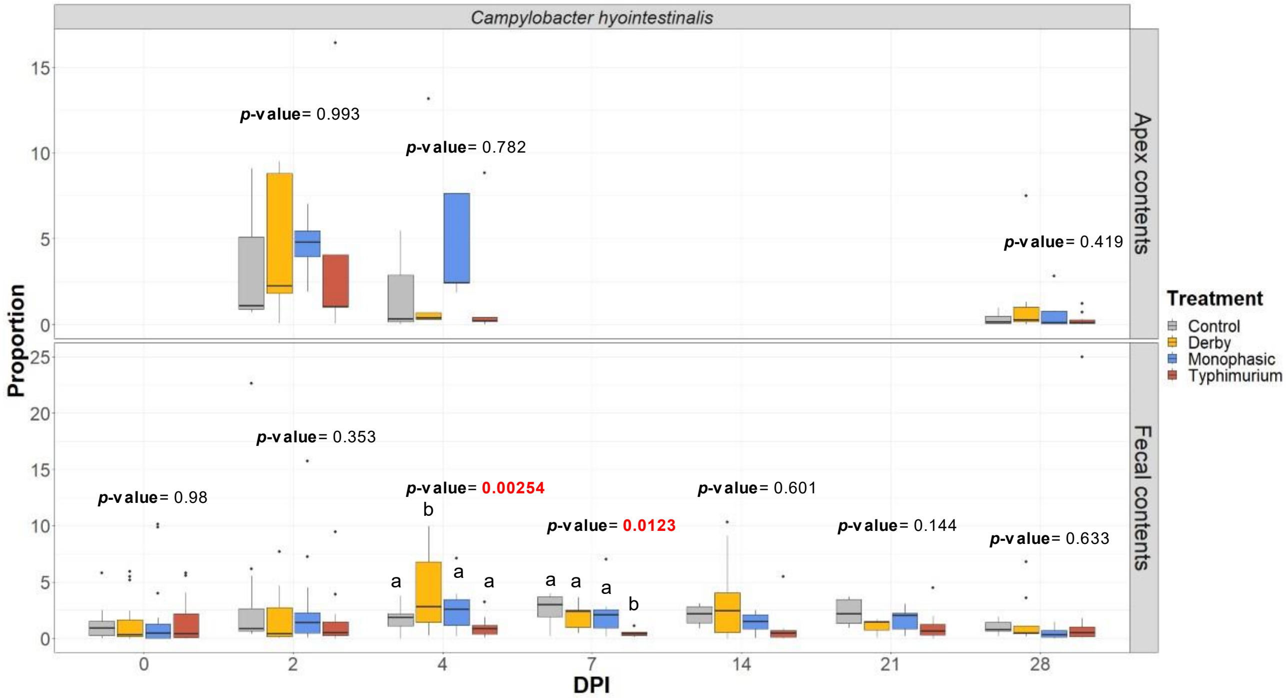


**Supplementary Figure 52 |** *Campylobacter hyointestinalis* proportion across treatments (Control, Derby, Monophasic, and Typhimurium) and DPI for both colonic apex (apex) and fecal contents. Statistical analysis was done using an ANOVA followed by a pairwise T-test ($p < 0.05$). Different superscript letters indicate significant differences between treatments. Only animals that had microbiome samples passed through the bioinformatic cut-off for quality control were included in this analysis.

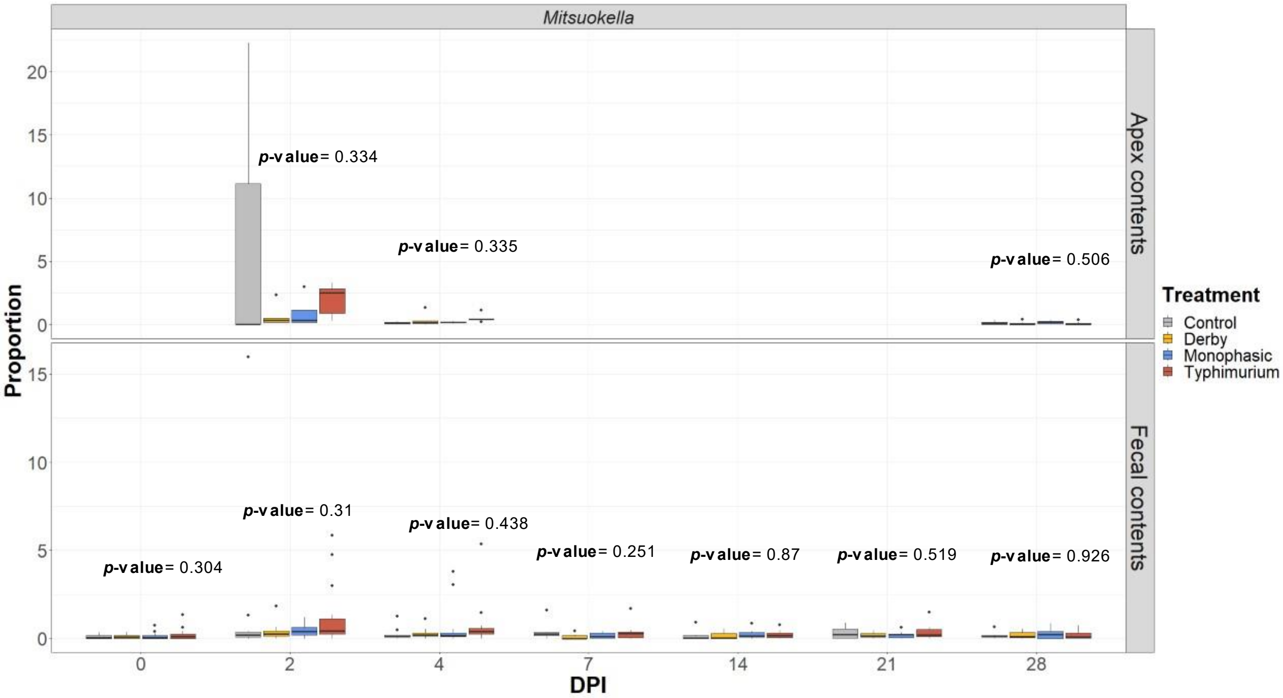


**Supplementary Figure 53 |** *Mitsuokella* proportion across treatments (Control, Derby, Monophasic, and Typhimurium) and DPI for both colonic apex (apex) and fecal contents. Statistical analysis was done using an ANOVA followed by a pairwise T-test ($p < 0.05$). Different superscript letters indicate significant differences between treatments. Only animals that had microbiome samples passed through the bioinformatic cut-off for quality control were included in this analysis.

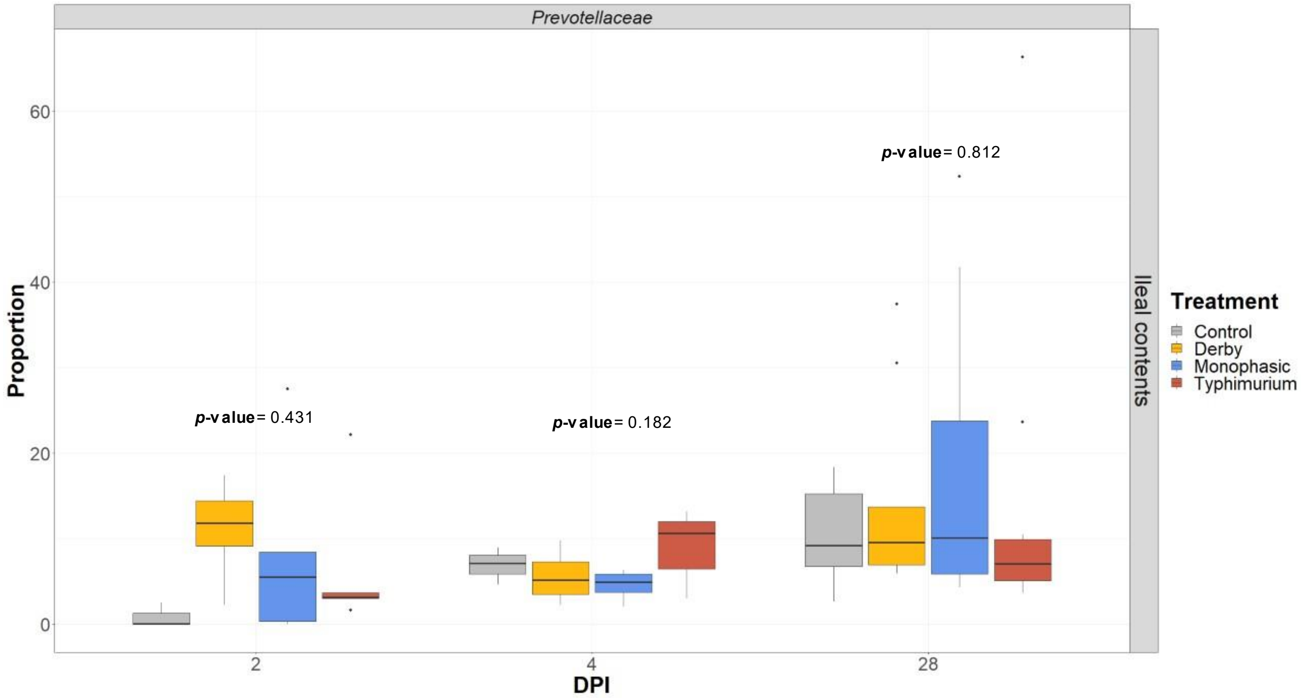


**Supplementary Figure 54 |** *Prevotellaceae* proportion across treatments (Control, Derby, Monophasic, and Typhimurium) and DPI for ileal contents. Statistical analysis was done using an ANOVA followed by a pairwise T-test ($p < 0.05$). Different superscript letters indicate significant differences between treatments. Only animals that had microbiome samples passed through the bioinformatic cut-off for quality control were included in this analysis.

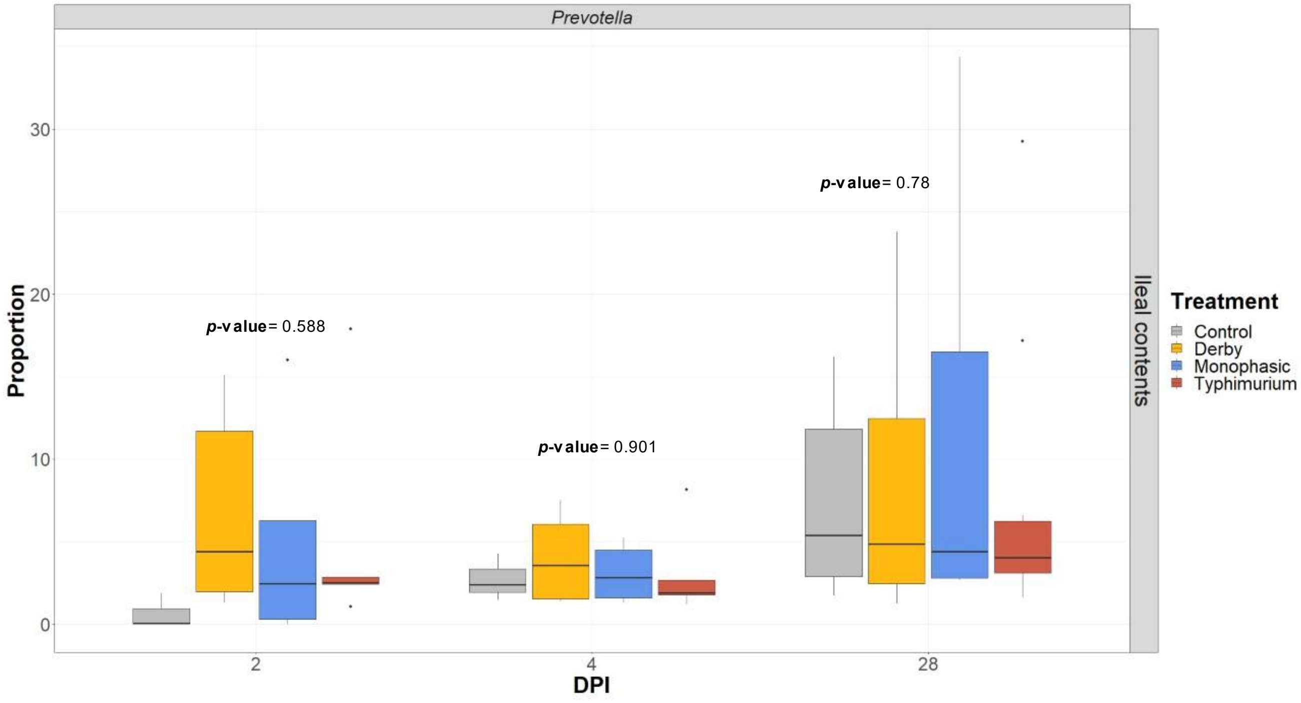


**Supplementary Figure 55 |** *Prevotella* proportion across treatments (Control, Derby, Monophasic, and Typhimurium) and DPI for ileal contents. Statistical analysis was done using an ANOVA followed by a pairwise T-test ($p < 0.05$). Different superscript letters indicate significant differences between treatments. Only animals that had microbiome samples passed through the bioinformatic cut-off for quality control were included in this analysis.

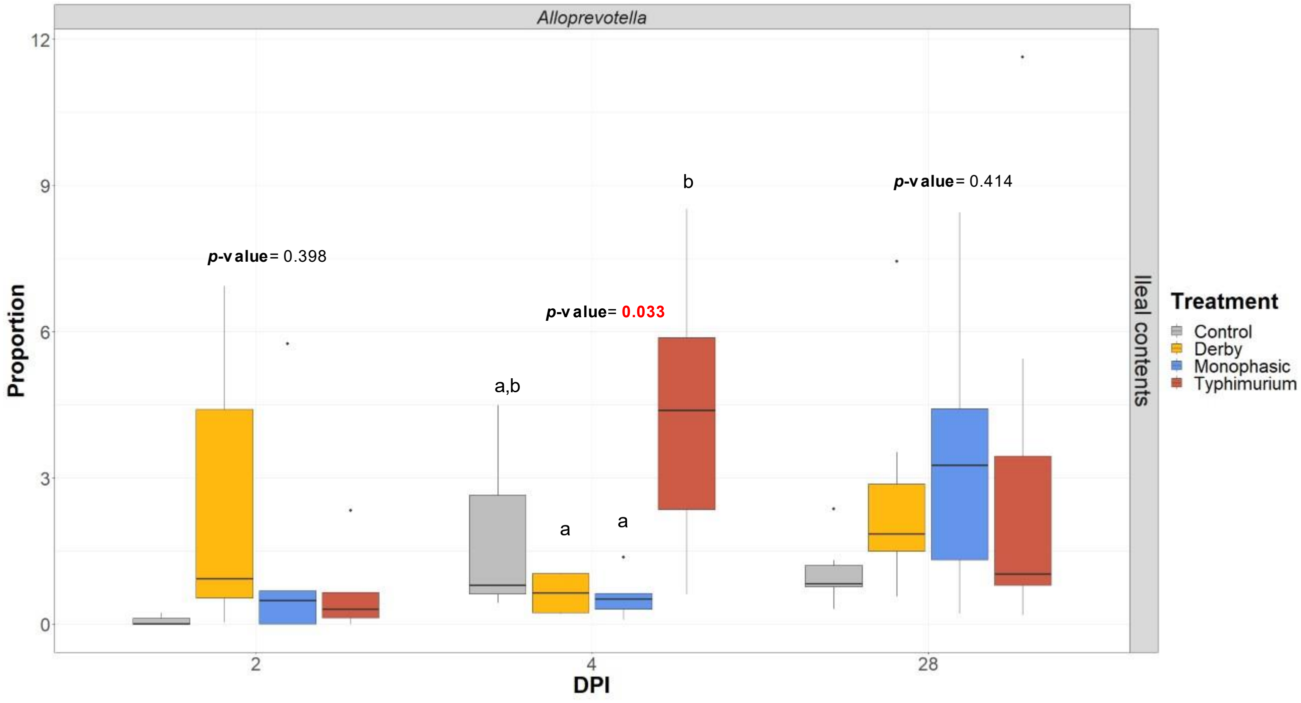


**Supplementary Figure 56 |** *Alloprevotella* proportion across treatments (Control, Derby, Monophasic, and Typhimurium) and DPI for ileal contents. Statistical analysis was done using an ANOVA followed by a pairwise T-test ($p < 0.05$). Different superscript letters indicate significant differences between treatments. Only animals that had microbiome samples passed through the bioinformatic cut-off for quality control were included in this analysis.

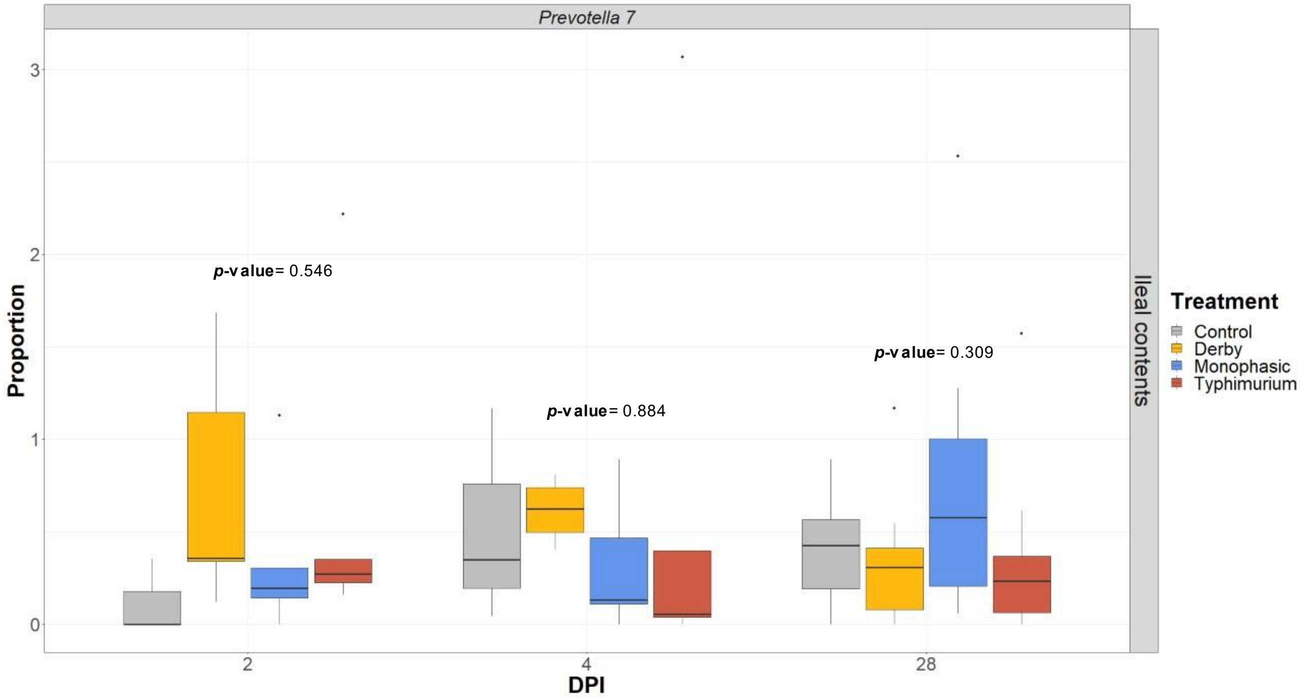


**Supplementary Figure 57 |** *Prevotella 7* proportion across treatments (Control, Derby, Monophasic, and Typhimurium) and DPI for ileal contents. Statistical analysis was done using an ANOVA followed by a pairwise T-test ($p < 0.05$). Different superscript letters indicate significant differences between treatments. Only animals that had microbiome samples passed through the bioinformatic cut-off for quality control were included in this analysis.

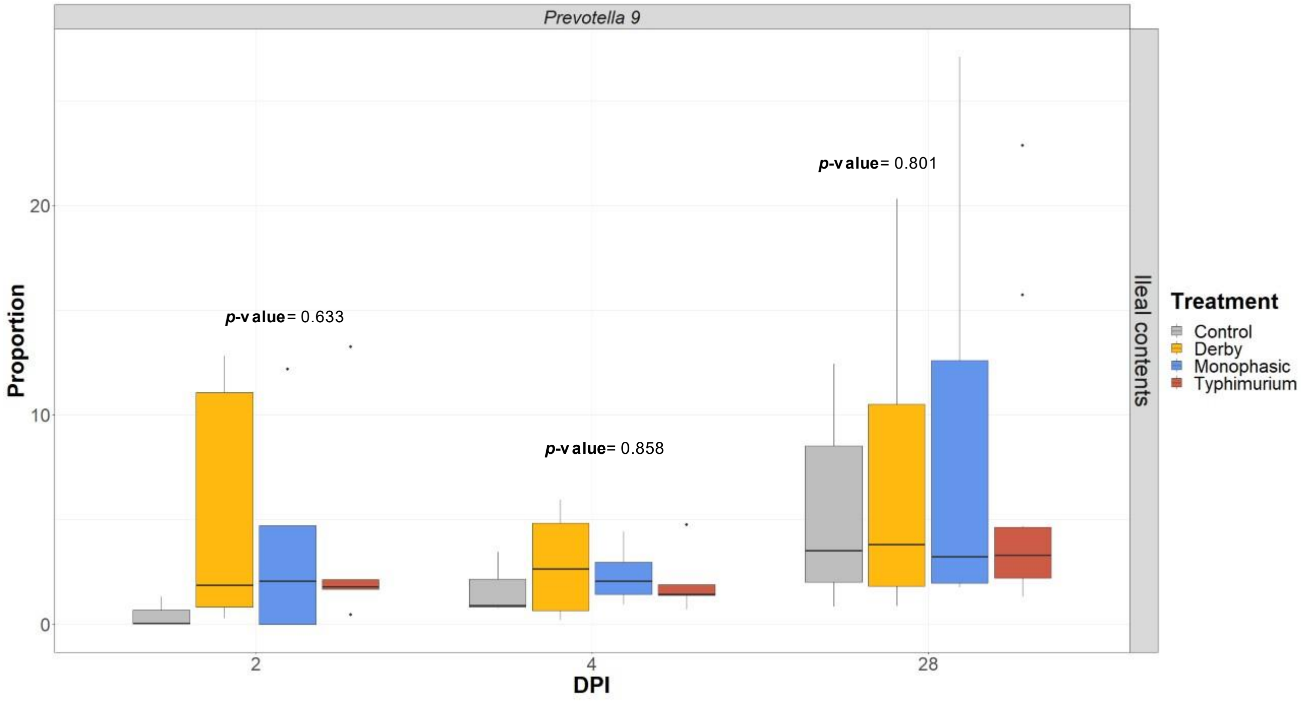


**Supplementary Figure 58 |** *Prevotella 9* proportion across treatments (Control, Derby, Monophasic, and Typhimurium) and DPI for ileal contents. Statistical analysis was done using an ANOVA followed by a pairwise T-test ($p < 0.05$). Different superscript letters indicate significant differences between treatments. Only animals that had microbiome samples passed through the bioinformatic cut-off for quality control were included in this analysis.

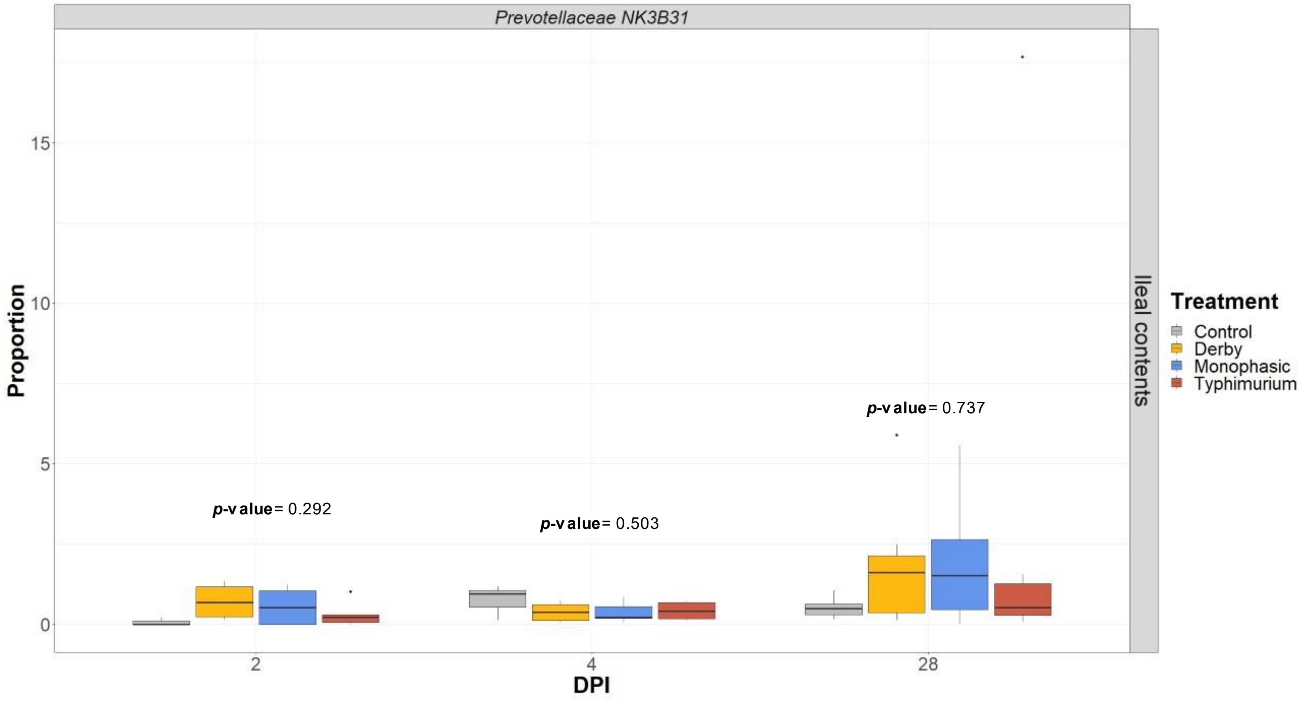


**Supplementary Figure 59 |** *Prevotellaceae NK3B31* proportion across treatments (Control, Derby, Monophasic, and Typhimurium) and DPI for ileal contents. Statistical analysis was done using an ANOVA followed by a pairwise T-test ($p < 0.05$). Different superscript letters indicate significant differences between treatments. Only animals that had microbiome samples passed through the bioinformatic cut-off for quality control were included in this analysis.

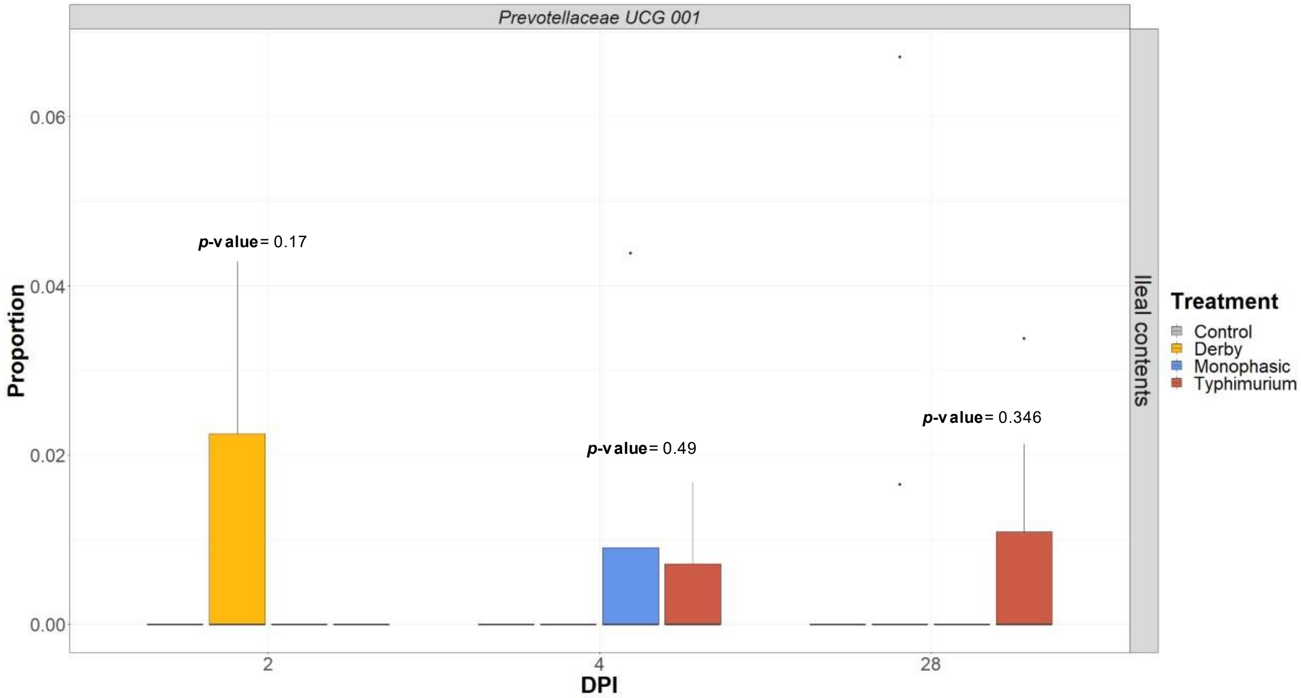


**Supplementary Figure 60 |** *Prevotellaceae UCG 001* proportion across treatments (Control, Derby, Monophasic, and Typhimurium) and DPI for ileal contents. Statistical analysis was done using an ANOVA followed by a pairwise T-test ($p < 0.05$). Different superscript letters indicate significant differences between treatments. Only animals that had microbiome samples passed through the bioinformatic cut-off for quality control were included in this analysis.

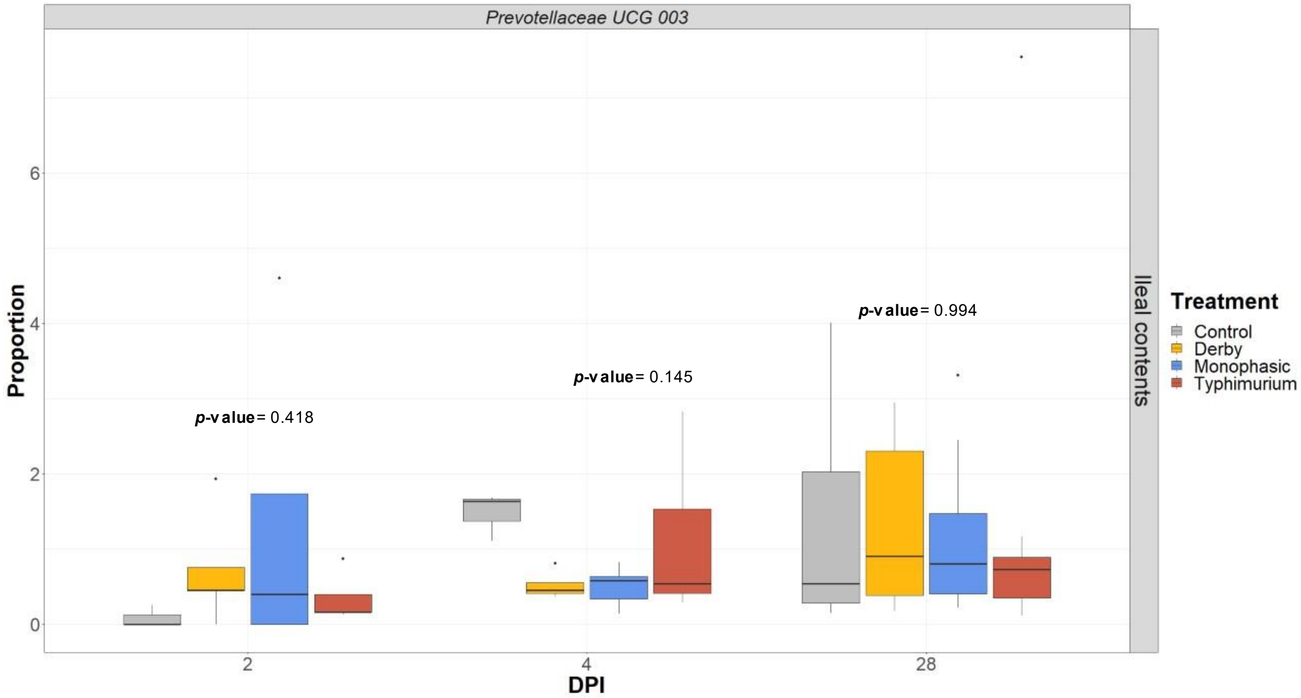


**Supplementary Figure 61 |** *Prevotellaceae UCG 003* proportion across treatments (Control, Derby, Monophasic, and Typhimurium) and DPI for ileal contents. Statistical analysis was done using an ANOVA followed by a pairwise T-test ($p < 0.05$). Different superscript letters indicate significant differences between treatments. Only animals that had microbiome samples passed through the bioinformatic cut-off for quality control were included in this analysis.

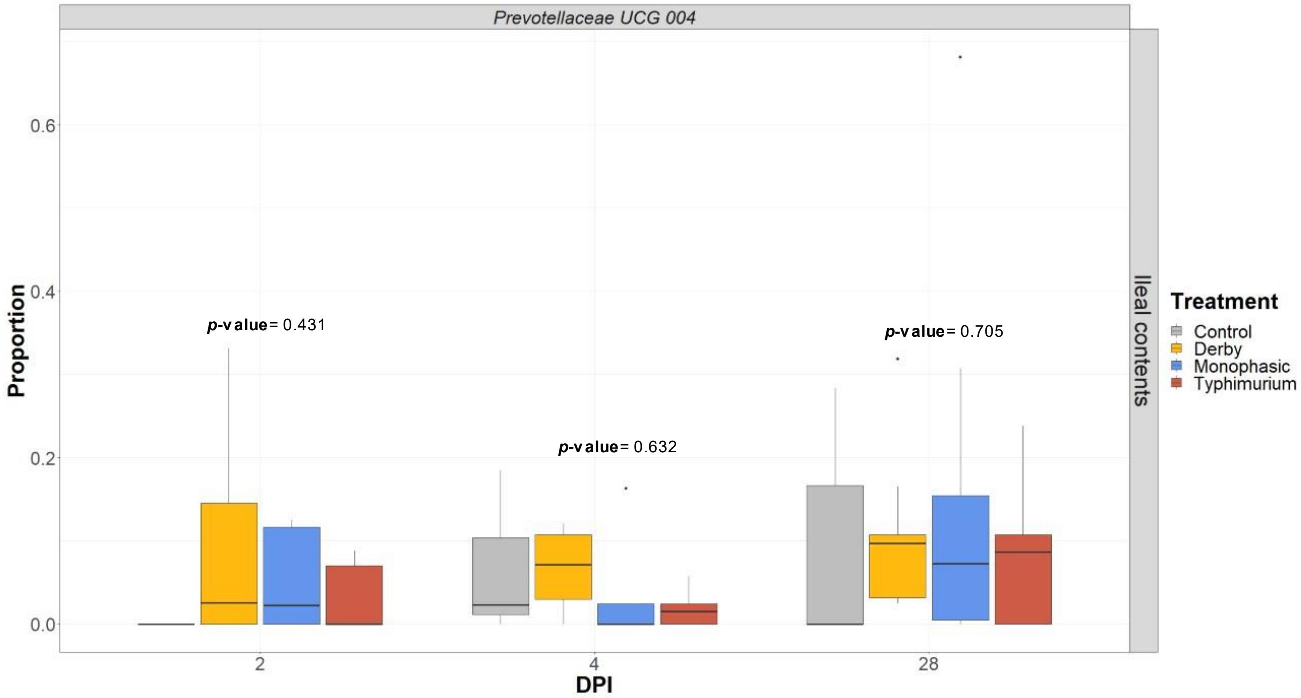


**Supplementary Figure 62 |** *Prevotellaceae UCG 004* proportion across treatments (Control, Derby, Monophasic, and Typhimurium) and DPI for ileal contents. Statistical analysis was done using an ANOVA followed by a pairwise T-test ($p < 0.05$). Different superscript letters indicate significant differences between treatments. Only animals that had microbiome samples passed through the bioinformatic cut-off for quality control were included in this analysis.

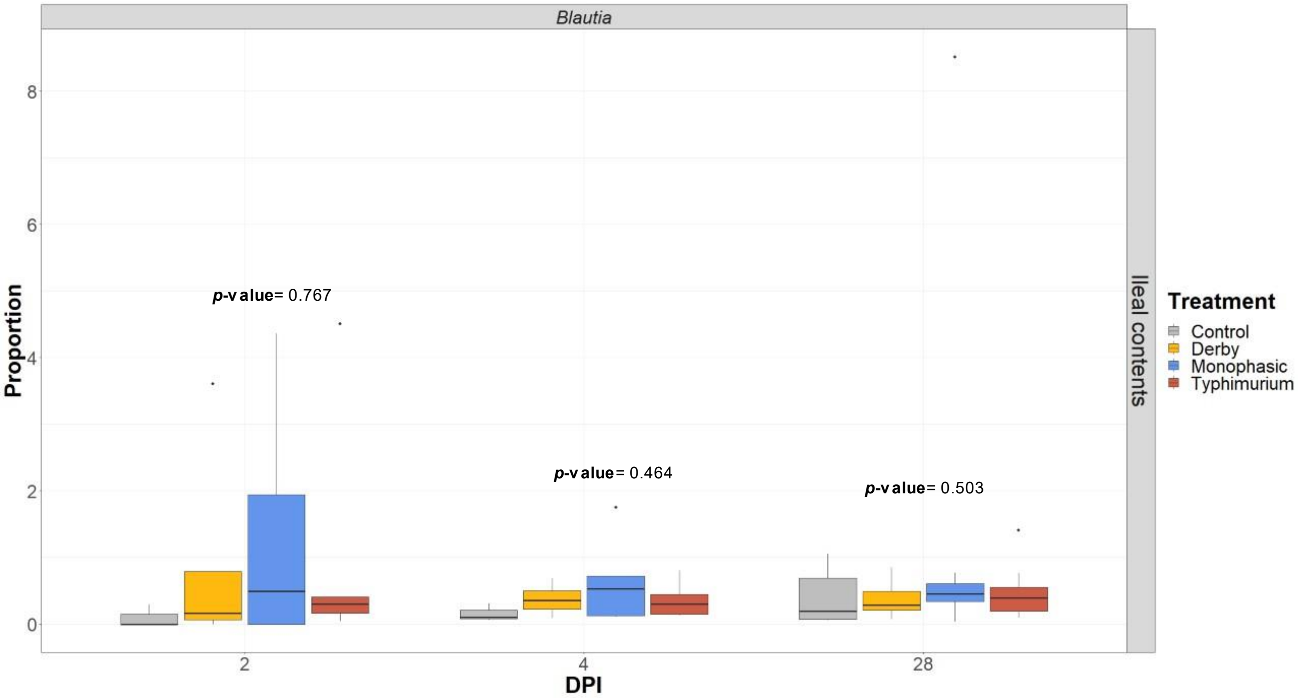


**Supplementary Figure 63 |** *Blautia* proportion across treatments (Control, Derby, Monophasic, and Typhimurium) and DPI for ileal contents. Statistical analysis was done using an ANOVA followed by a pairwise T-test ($p < 0.05$). Different superscript letters indicate significant differences between treatments. Only animals that had microbiome samples passed through the bioinformatic cut-off for quality control were included in this analysis.

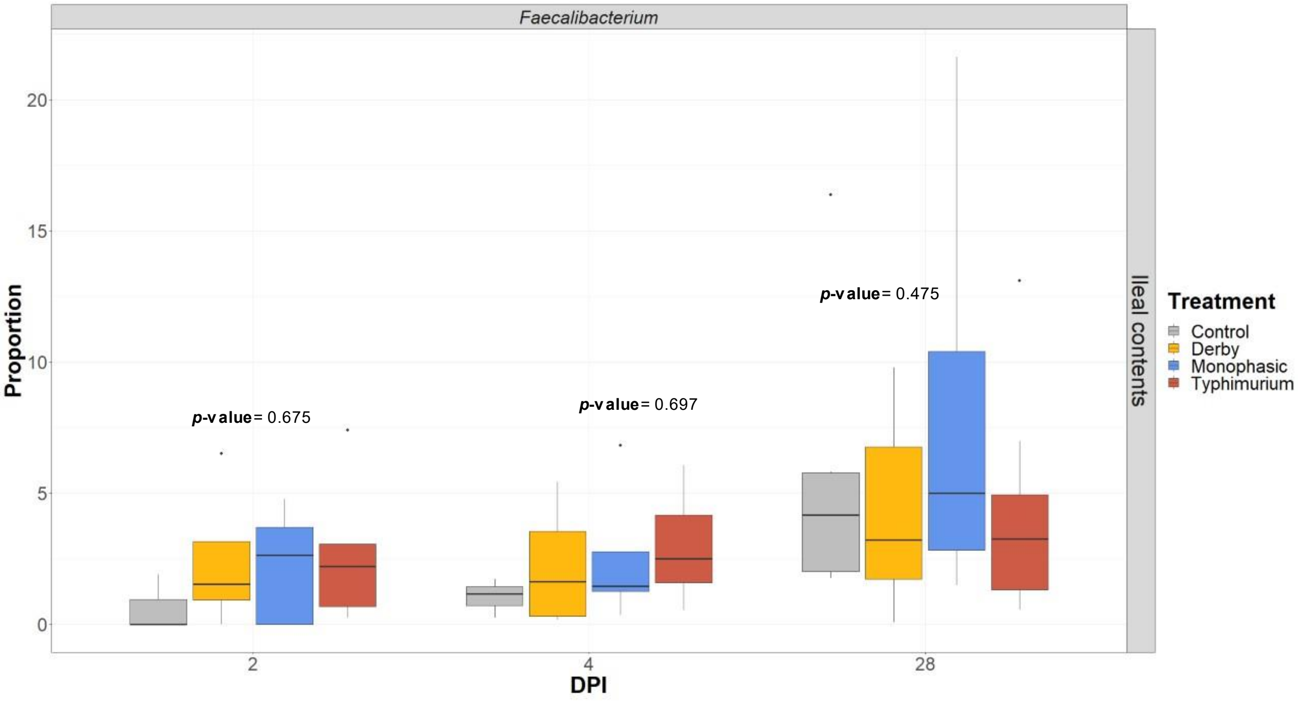


**Supplementary Figure 64 |** *Faecalibacterium* proportion across treatments (Control, Derby, Monophasic, and Typhimurium) and DPI for ileal contents. Statistical analysis was done using an ANOVA followed by a pairwise T-test ($p < 0.05$). Different superscript letters indicate significant differences between treatments. Only animals that had microbiome samples passed through the bioinformatic cut-off for quality control were included in this analysis.

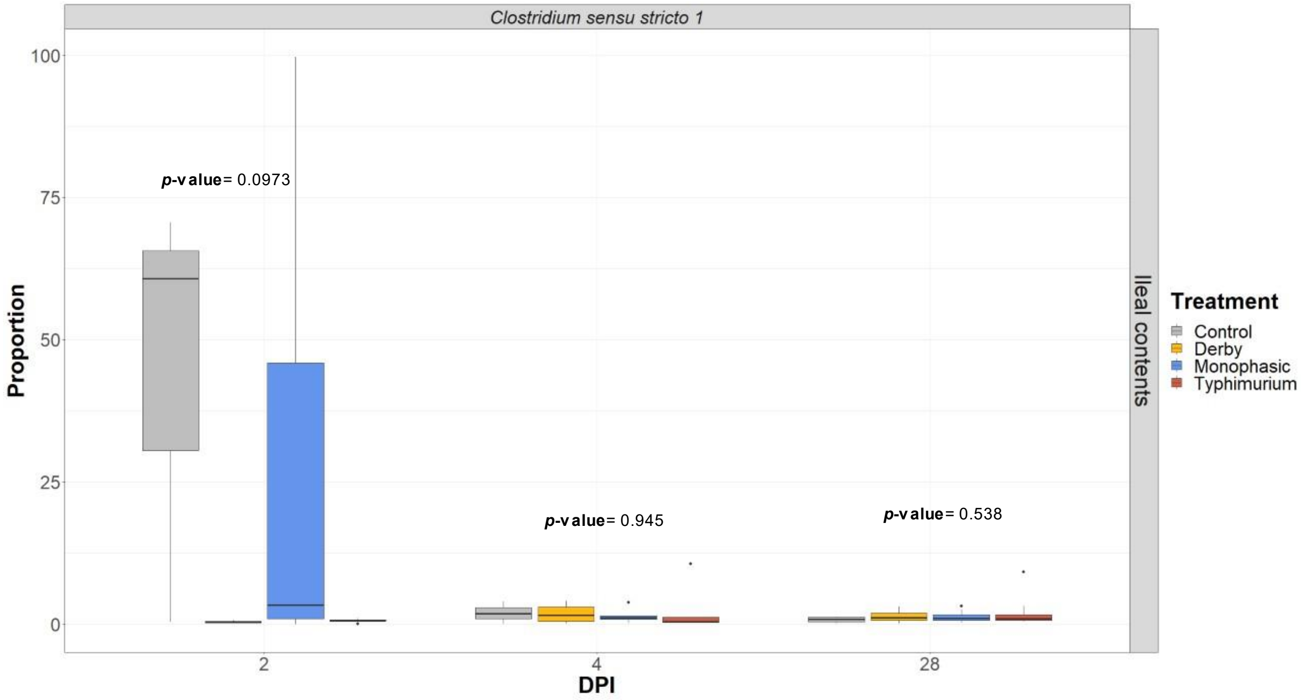


**Supplementary Figure 65 |** *Clostridium sensu stricto 1* proportion across treatments (Control, Derby, Monophasic, and Typhimurium) and DPI for ileal contents. Statistical analysis was done using an ANOVA followed by a pairwise T-test ($p < 0.05$). Different superscript letters indicate significant differences between treatments. Only animals that had microbiome samples passed through the bioinformatic cut-off for quality control were included in this analysis.

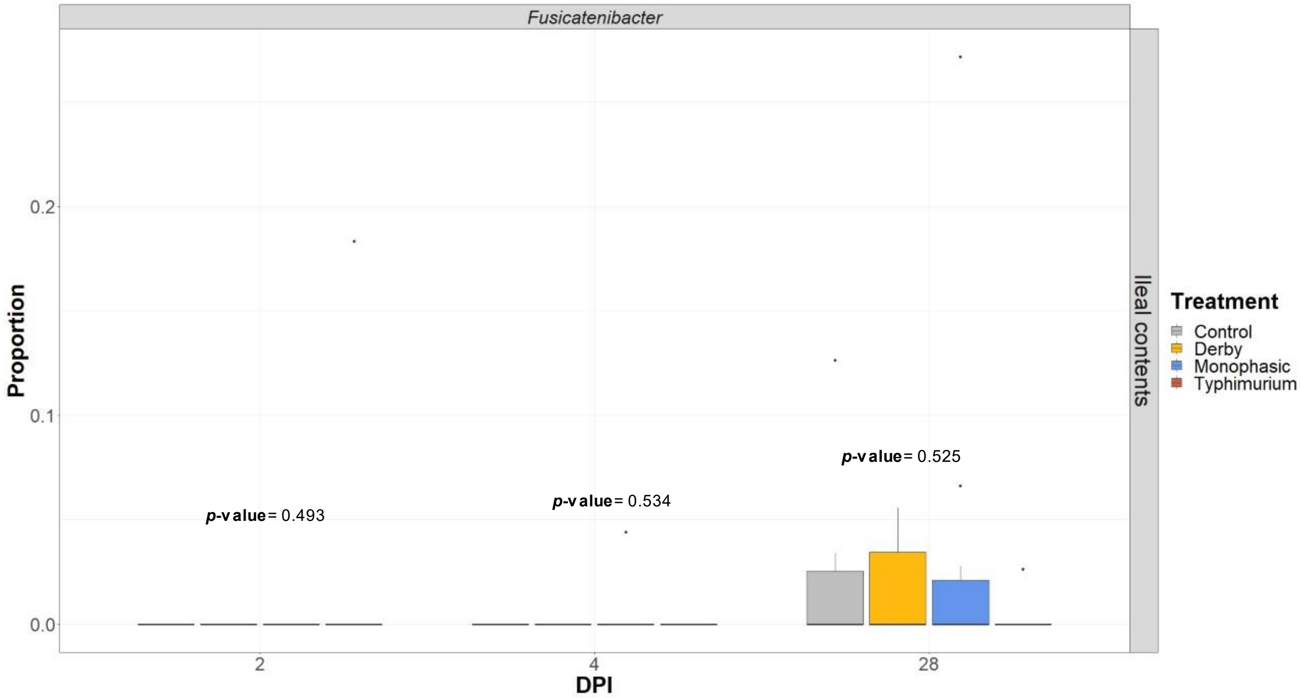


**Supplementary Figure 66 |** *Fusicatenibacter* proportion across treatments (Control, Derby, Monophasic, and Typhimurium) and DPI for ileal contents. Statistical analysis was done using an ANOVA followed by a pairwise T-test ($p < 0.05$). Different superscript letters indicate significant differences between treatments. Only animals that had microbiome samples passed through the bioinformatic cut-off for quality control were included in this analysis.

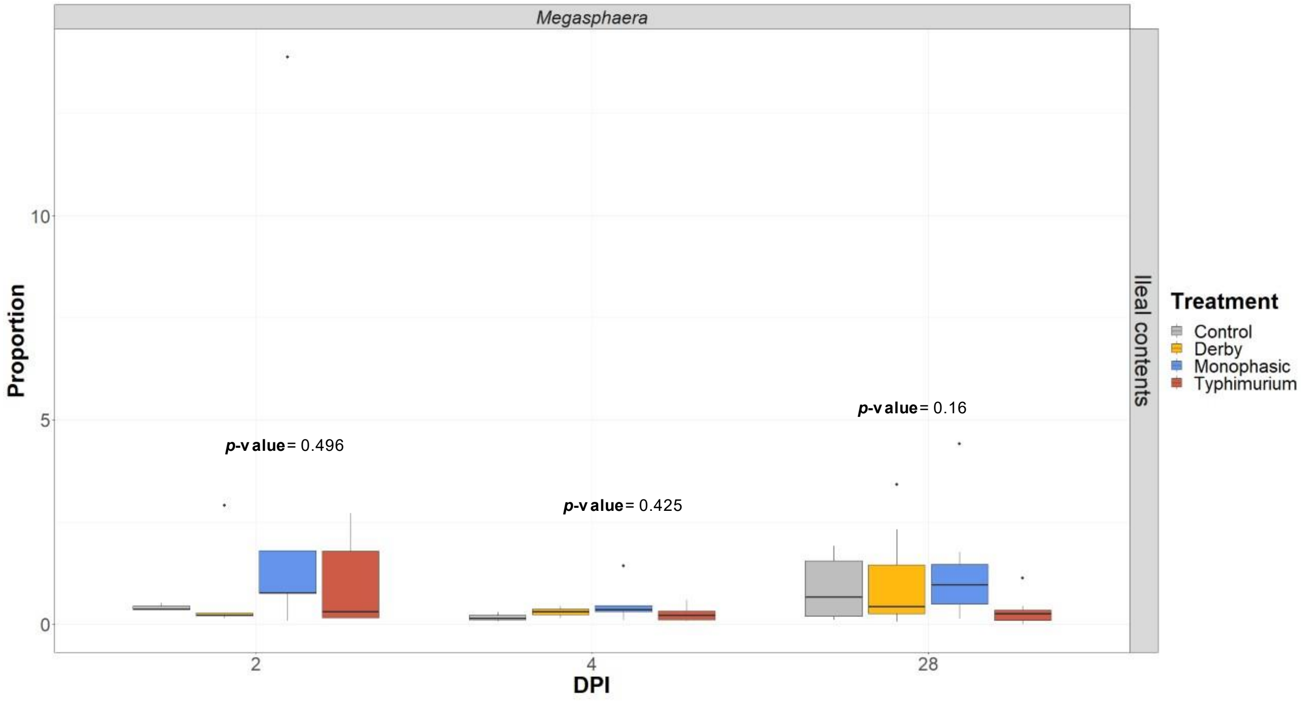


**Supplementary Figure 67 |** *Megasphaera* proportion across treatments (Control, Derby, Monophasic, and Typhimurium) and DPI for ileal contents. Statistical analysis was done using an ANOVA followed by a pairwise T-test ($p < 0.05$). Different superscript letters indicate significant differences between treatments. Only animals that had microbiome samples passed through the bioinformatic cut-off for quality control were included in this analysis.

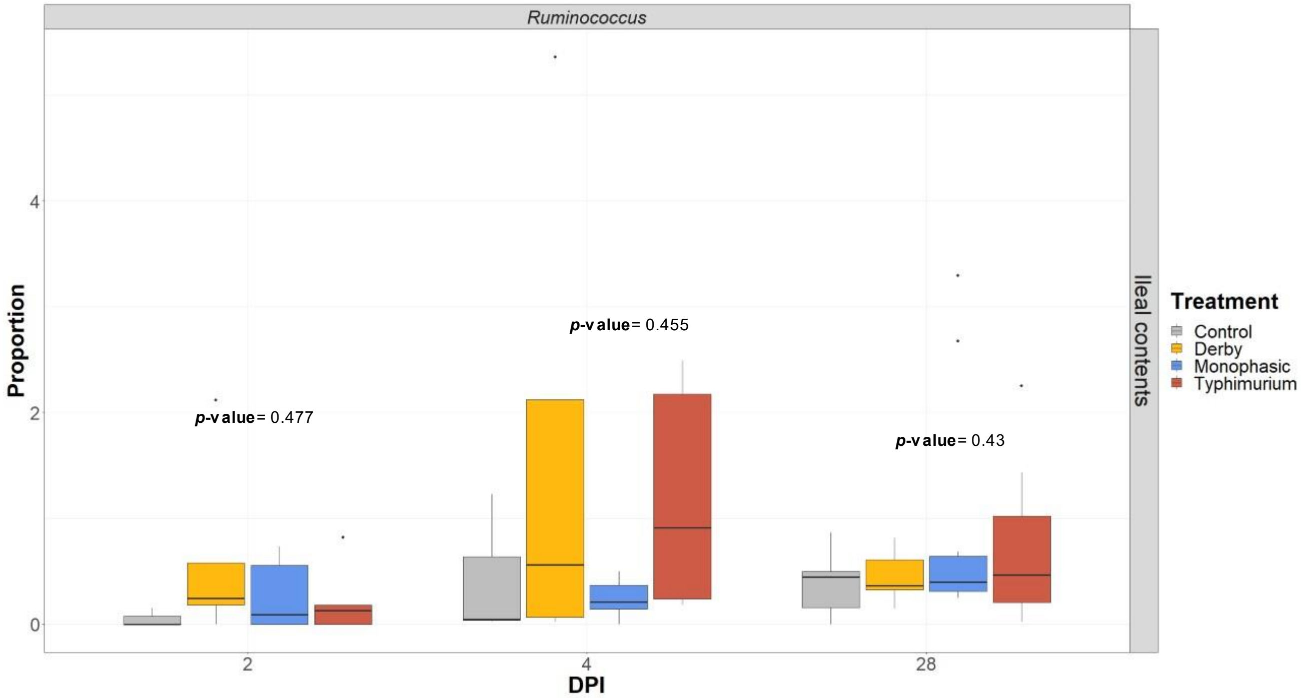


**Supplementary Figure 68 |** *Ruminococcus* proportion across treatments (Control, Derby, Monophasic, and Typhimurium) and DPI for ileal contents. Statistical analysis was done using an ANOVA followed by a pairwise T-test ($p < 0.05$). Different superscript letters indicate significant differences between treatments. Only animals that had microbiome samples passed through the bioinformatic cut-off for quality control were included in this analysis.

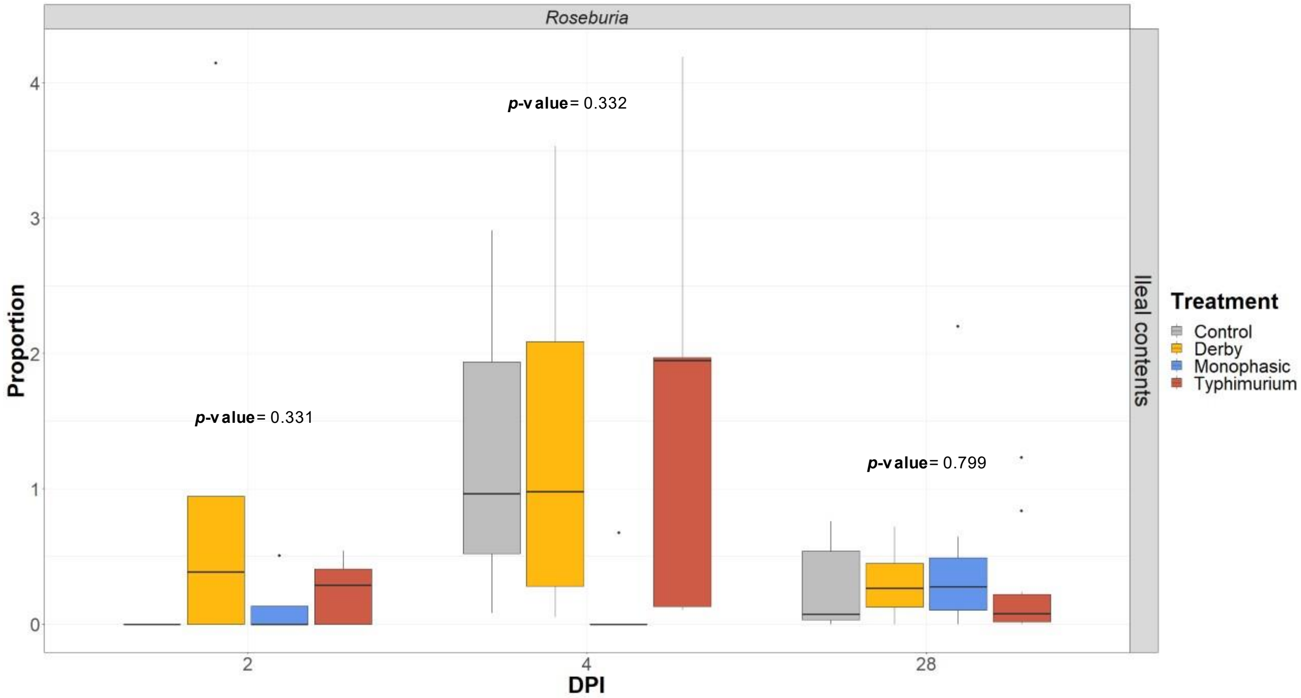


**Supplementary Figure 69 |** *Roseburia* proportion across treatments (Control, Derby, Monophasic, and Typhimurium) and DPI for ileal contents. Statistical analysis was done using an ANOVA followed by a pairwise T-test ($p < 0.05$). Different superscript letters indicate significant differences between treatments. Only animals that had microbiome samples passed through the bioinformatic cut-off for quality control were included in this analysis.

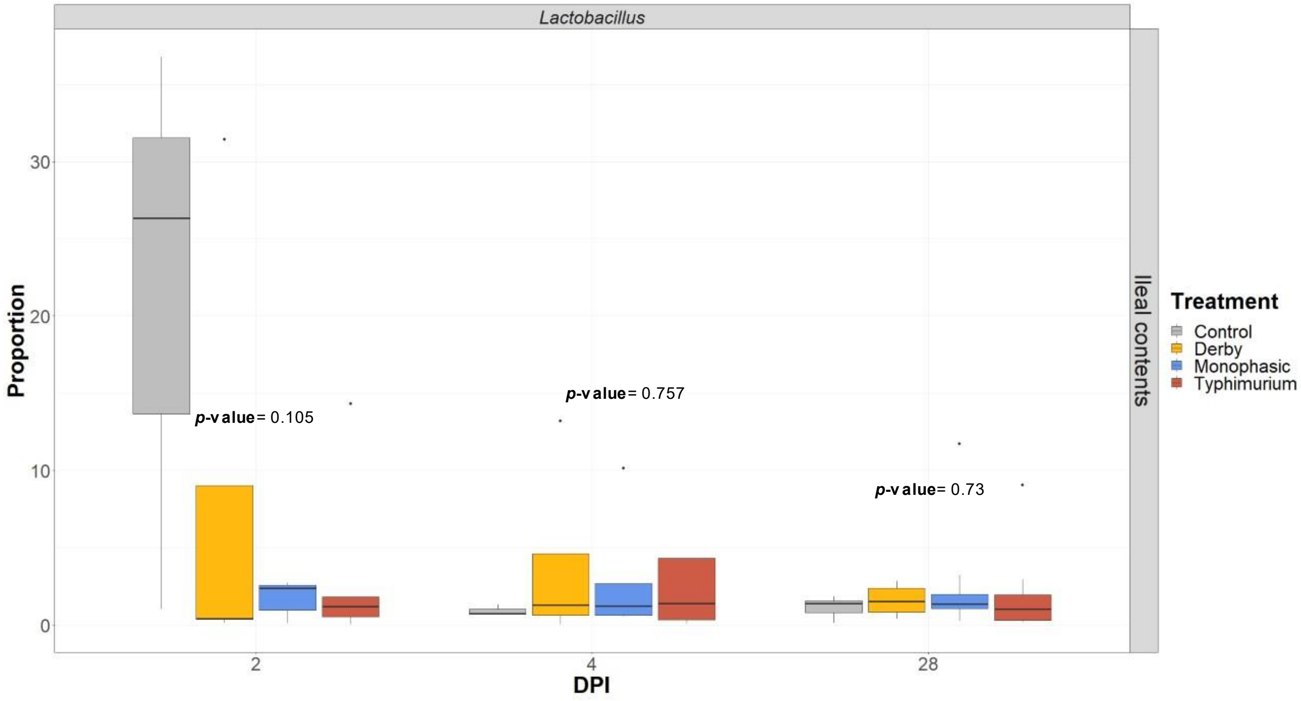


**Supplementary Figure 70 |** *Lactobacillus* proportion across treatments (Control, Derby, Monophasic, and Typhimurium) and DPI for ileal contents. Statistical analysis was done using an ANOVA followed by a pairwise T-test ($p < 0.05$). Different superscript letters indicate significant differences between treatments. Only animals that had microbiome samples passed through the bioinformatic cut-off for quality control were included in this analysis.

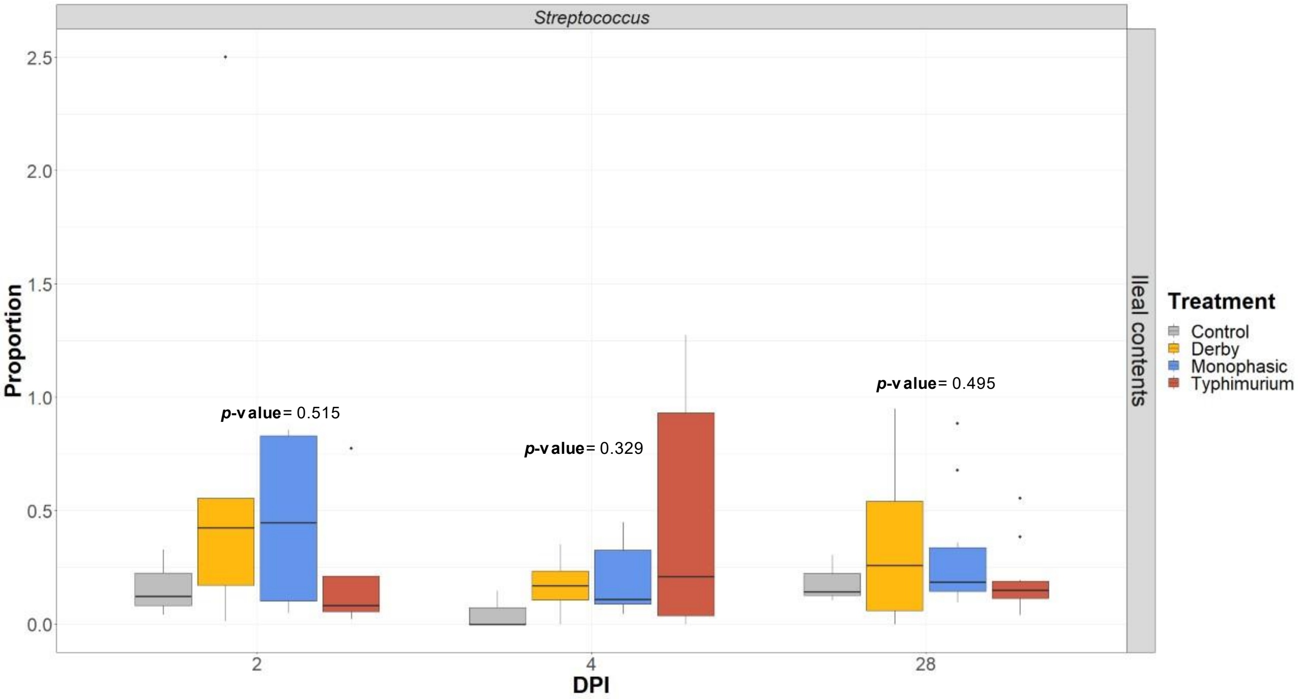


**Supplementary Figure 71 |** *Streptococcus* proportion across treatments (Control, Derby, Monophasic, and Typhimurium) and DPI for ileal contents. Statistical analysis was done using an ANOVA followed by a pairwise T-test ($p < 0.05$). Different superscript letters indicate significant differences between treatments. Only animals that had microbiome samples passed through the bioinformatic cut-off for quality control were included in this analysis.

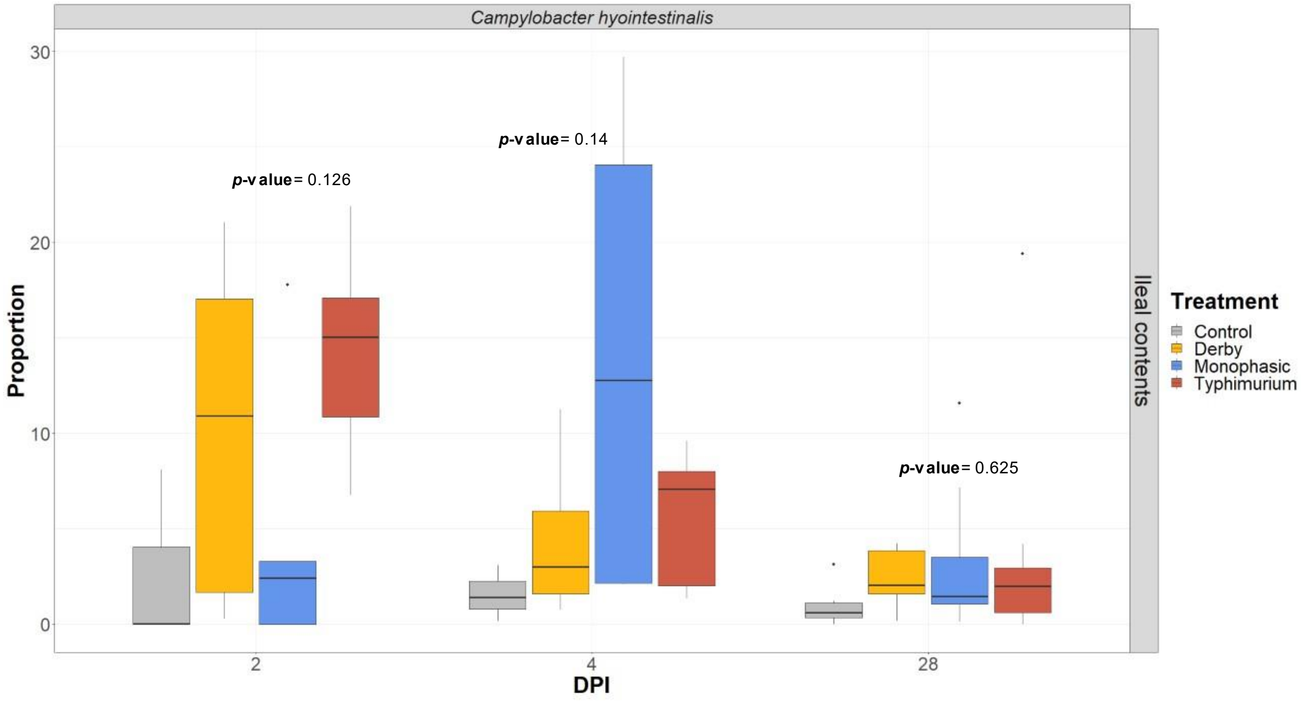


**Supplementary Figure 72 |** *Campylobacter hyointestinalis* proportion across treatments (Control, Derby, Monophasic, and Typhimurium) and DPI for ileal contents. Statistical analysis was done using an ANOVA followed by a pairwise T-test ($p < 0.05$). Different superscript letters indicate significant differences between treatments. Only animals that had microbiome samples passed through the bioinformatic cut-off for quality control were included in this analysis.

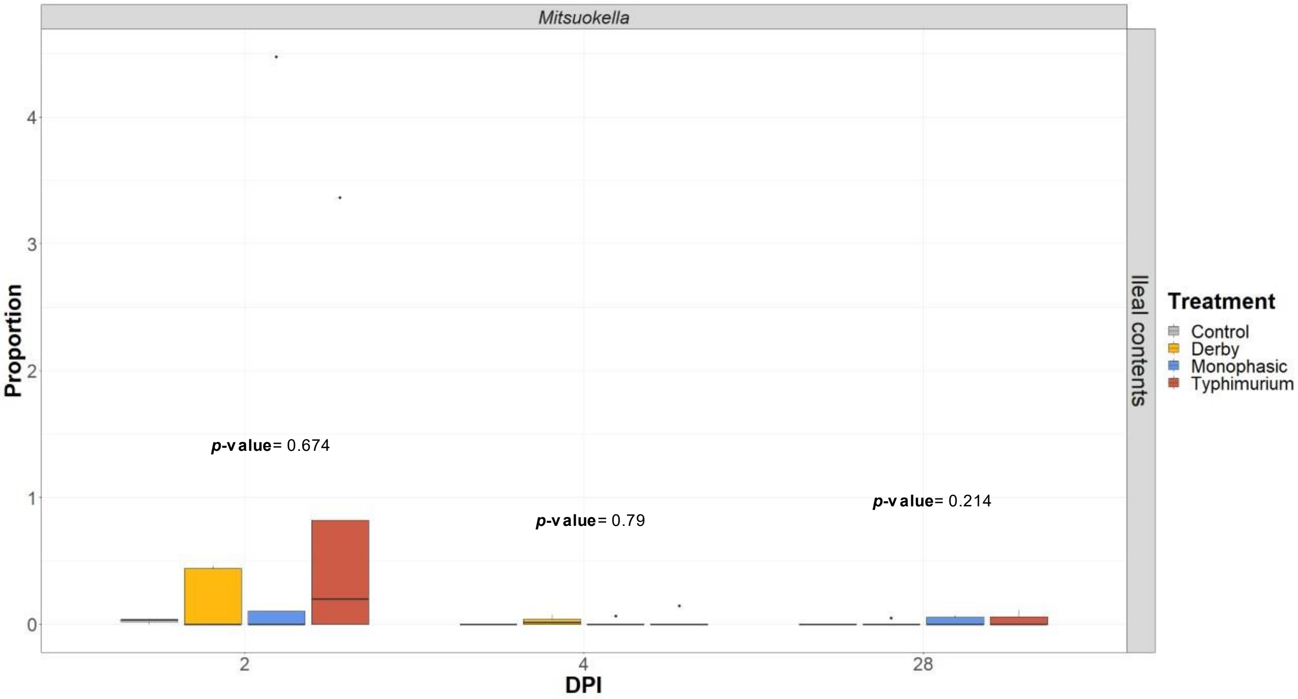


**Supplementary Figure 73 |** *Mitsuokella* proportion across treatments (Control, Derby, Monophasic, and Typhimurium) and DPI for ileal contents. Statistical analysis was done using an ANOVA followed by a pairwise T-test ($p < 0.05$). Different superscript letters indicate significant differences between treatments. Only animals that had microbiome samples passed through the bioinformatic cut-off for quality control were included in this analysis.

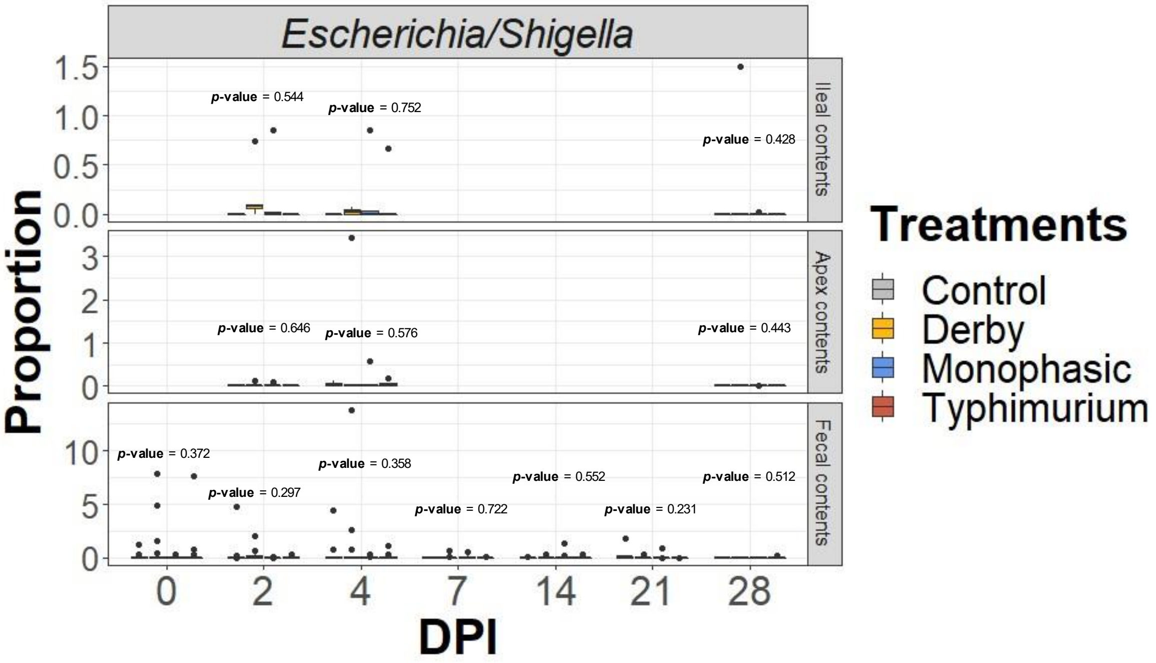


**Supplementary Figure 74 |** Genus *Escherichia/Shigella* proportion across treatments (Control, Derby, Monophasic, and Typhimurium) and DPI for ileal, apex, and fecal contents. Statistical analysis was done using an ANOVA followed by a pairwise T-test ($p < 0.05$). Different superscript letters indicate significant differences between treatments. Only animals that had microbiome samples passed through the bioinformatic cut-off for quality control were included in this analysis.
